# 6th International Symposium on Molecular Allergology (ISMA)

**DOI:** 10.1186/s13601-016-0123-x

**Published:** 2016-10-18

**Authors:** Christiane Hilger, Kyra Swiontek, Jörg Fischer, François Hentges, Christiane Lehners, Martine Morisset, Bernadette Eberlein, Tilo Biedermann, Markus Ollert, Sabrina Wildner, Teresa Stemeseder, Regina Freier, Peter Briza, Roland Lang, Eva Batanero, Mayte Villalba, Jonas Lidholm, Thomas Hawranek, Fatima Ferreira, Hans Brandstetter, Gabriele Gadermaier, Philippe Moingeon, Rachel Groeme, Julien Bouley, Véronique Bordas, Maxime Le Mignon, Laetitia Bussières, Aurélie Lautrette, Laurent Mascarell, Vincent Lombardi, Véronique Baron-Bodo, Henri Chabre, Thierry Batard, Emmanuel Nony, Karine Marafigo De Amicis, Alexandra Sayuri Watanabe, Daniele Danella Figo, José Roberto Aparecido Dos Santos-Pinto, Mario Sergio Palma, Fabio Fernandes Morato Castro, Jorge Kalil, Therese Wohlschlager, Fatima Ferreira-Briza, Keity Souza Santos, Margaretha Faber, Athina Van Gasse, Vito Sabato, Margo M. Hagendorens, Chris H. Bridts, Luc S. De Clerck, Araceli Diaz Perales, Didier Ebo, Petra Zavadakova, Aurélie Buchwalder, Fabien Rebeaud, Iwan Märki, Barbara Gepp, Nina Lengger, Christian Möbs, Wolfgang Pfützner, Christian Radauer, Barbara Bohle, Clovis Eduardo Galvao, Jose Roberto Aparecido Santos-Pinto, Christian Schwager, Skadi Kull, Frauke Schocker, Jochen Behrends, Wolf-Meinhard Becker, Uta Jappe, Carla Mastrorilli, Salvatore Tripodi, Carlo Caffarelli, Riccardo Asero, Arianna Dondi, Giampaolo Ricci, Carlotta Povesi Dascola, Elisabetta Calamelli, Andrea Di Rienzo Businco, Annamaria Bianchi, Tullio Frediani, Carmen Verga, Iride Dello Iacono, Diego Peroni, Giuseppe Pingitore, Roberto Bernardini, Paolo Maria Matricardi, Heidi Hofer, Claudia Asam, Michael Hauser, Martin Himly, Christof Ebner, Pierrick Lemoine, Karine Jain, Kathy Abiteboul, Monica Arvidsson, Sabina Rak, Inês Mota, Filipe Benito Garcia, Angela Gaspar, Cristina Arêde, Susana Piedade, Graça Sampaio, Graça Pires, Luís Miguel Borrego, Cristina Santa-Marta, Mário Morais-Almeida, Florin-Dan Popescu, Mariana Vieru, Florin-Adrian Secureanu, Rosa Anita Rodrigues Fernandes, Isabel Carrapatoso, Raquel Gomes, Celso Pereira, Ana Todo-Bom, María Cecilia Martín Fernández De Basoa, Javier Barrios Regio, Juan De Castro Cordova, Antón Fernández Ferreiro, Olympia Tsilochristou, Serena Perna, Alina Schwarz, Alexander Rohrbach, Antonio Cappella, Laura Hatzler, Carl-Peter Bauer, Ute Hoffmann, Johannes Forster, Fred Zepp, Antje Schuster, Raffael D’amelio, Ulrich Wahn, Thomas Keil, Susanne Lau, Pol André Apoil, Claire Mailhol, Anne Broué-Chabbert, Agnès Juchet, Alain Didier, Elodie Carrer, Thomas Lanot, Antoine Blancher, Almedina Kurtaj, Christoph Hillebrand, Gerda Fichtinger, Martin Danzer, Christian Gabriel, Theresa Thalhamer, Sandra Scheiblhofer, Josef Thalhamer, Richard Weiss, Martin Wolf, Ulrike Pichler, Teresa Twaroch, Hidenori Yokoi, Toshiro Takai, Alain Didierlaurent, Adriano Mari, Heidrun Behrendt, Angela Neubauer, Frank Stolz, Fátima Ferreira, Michael Wallner, Sara Carvalho, Tatiana Lourenço, Joana Cosme, Fátima Cabral Duarte, Amélia Spínola Santos, Ana Célia Costa, Manuel Pereira Barbosa, Eva Klinglmayr, Bettina Schweidler, Lisa Lueftenegger, Stephanie Moser, Patrick Doppler, Gertie J. Oostingh, Arne Bathke, Joerg Zumbach, Petr Panzner, Martina Vachova, Tomas Vlas, Marek Maly, Daniela Posa, Stephanie Hofmaier, Philippe Stock, Linus Grabenhenrich, Kuan-Wei Chen, Yvonne Resch, Susanne Vrtala, Rudolf Valenta, Tamar Abramidze, Nino Lomidze, Maia Gotua, Austeja Dapkeviciute, Ruta Einikyte, Jolita Norkuniene, Laima Skrickiene, Asta Miskiniene, Violeta Kvedariene, Maximilian Schiener, Carmen Moreno-Aguilar, Gunilla Pietsch, Mareike Mc Intyre, Lea Schwarze, Dennis Rußkamp, Edzard Spillner, Ulf Darsow, Carsten Schmidt-Weber, Simon Blank, Cyril Longé, Andrea Brazdova, Jean-Louis Brunet, Claire Schwartz, Bruno Girodet, François Lavaud, Joelle Birnbaum, Nhân Pham Thi, Magalie Duchateau, Julia Chamot-Rooke, Laurence Guilloux, Marie-Ange Selva, Rémy Couderc, Hélène Sénéchal, Jean-Pierre Sutra, Pascal Poncet, Steffen Augustin, Linda Pump, Martin Wald, Thomas Eichhorn, Frank Fischer, Christoph Willers, Michaela Miehe, Melanie Plum, Sara Wolf, Frederic Jabs, Tim Raiber, Frank Bantleon, Henning Seismann, Thilo Jakob, Danijela Apostolovic, Anh Thu Tran, Sara Sanchez-Vidaurre, Tanja Cirkovic Velickovic, Maria Starkhammar, Carl Hamsten, Marianne Van Hage, Pawel Dubiela, Piotr Humeniuk, Sabine Pfeifer, Merima Bublin, Tomasz Borowski, Karin Hoffmann-Sommergruber, Martie C. M. Verschuren, Shanna Bastiaan-Net, Defien Depoortere, Kay Foetisch, Stephan Scheurer, Harry J Wichers, Theo Noij, Nikki M.E. Van Uden, Karel Vandenberghe, Harry J. Wichers, Theo H. M. Noij, Anargyros Roulias, Maria Alejandra Parigiani, Linda Ahammer, Sarina Grutsch, Martin Tollinger, Raquel Moya, Mª Angeles López-Matas, Raquel Reyes, Jerónimo Carnés, Colette Larré, Hélène Rogniaux, Roberta Lupi, Sandra Denery-Papini, Isabel Maria Pablos, Stephanie Eichhorn, Yoan Machado, Jung-Won Park, Naveen Arora, Stefan Vieths, Charlene Tanaka, Florence Pineau, Martine Drouet, Etienne Beaudouin, Susan Altenbach, Hamza Mameri, Chantal Brossard, Jean Charles Gaudin, Denise Anne Moneret-Vautrin, Evelyne Paty, Olivier Tranquet, Stefania Masci, Denise-Anne Moneret-Vautrin, Arnd Petersen, Marisa Böttger, Sandra Rennert, Susanne Krause, Martin Ernst, Thomas Gutsmann, Johann Bauer, Buko Lindner, Stef Koppelman, Shyamali Jayasena, Dion Luykx, Erik Schepens, Govardus De Jong, Tom Isleib, Julie Nordlee, Joe Baumert, Steve Taylor, Soheila Maleki, Chiara Palladino, Sofía Sirvent, Alba Angelina, Thomas Eiwegger, Oscar Palomares, Heimo Breiteneder, Mathilde Claude, Grégory Bouchaud, Marie Bodinier, Robin Korte, Julia Bräcker, Jens Brockmeyer, Rie Satoh, Reiko Teshima, Angelika Tscheppe, Dieter Palmberger, Reingard Grabherr, Marianne Raith, Linda Sonnleitner, Doris Zach, Konrad Woroszylo, Margit Focke-Tejkl, Herbert Wank, Thorsten Graf, Annette Kuehn, Ines Swoboda, Sara Huber, Fabienne Gay-Crosier, Dominika Polak, Birgit Nagl, Claudia Kitzmüller, Nazanin Samadi, Rene Geyeregger, Beatrice Jahn-Schmid, Ariel Gomez, Jaana Haka, Liisa Hattara, Marika Heikkinen, Merja H Niemi, Juha Rouvinen, Petri Saviranta, Pekka Mattila, Kristiina Takkinen, Marja-Leena Laukkanen, Isabel Pablos, Bianca Kastner, Mira Silar, Julij Selb, Rok Kogovsek, Mitja Kosnik, Peter Korosec, Leticia Pestana, Alcinda Campos Melo, Ana Mendes, Maria Elisa Pedro, Maria Conceição Pereira Santos, Françoise Bienvenu, Claire Goursaud, Lorna Garnier, Sandrine Jacquenet, Michaël Degaud, Sébastien Viel, Annick Barre, Pierre Rougé, Jacques Bienvenu, Joana Vitte, Amel Bensalah, Isabelle Cleach, Laurent Mousseau, Chantal Agabriel, Valérie Liabeuf, Joëlle Birnbaum, Jean-Louis Mège, James Gardner, Minal Gandhi, Harsha Kariyawasam, Giuseppina Rotiroti, Frederico Regateiro, Emília Faria, Johannes Martin Schmid, Ronald Dahl, Hans Juergen Hoffmann, Letícia Pestana, Diana Silva, Teresa Vieira, Ana Maria Pereira, André Moreira, Luís Delgado, Sara Prates, Cátia Alves, Elena Finelli, Paula Leiria Pinto, Bárbara Kong Cardoso, Cíntia Cruz, Filipa Semedo, Elza Tomaz, Filipe Inácio, Santanu Maity, Ivona Baricevic-Jones, Justin T. Marsh, Phil E. Johnson, Anuradha Balasundaram, Anya-May Hope, Aafke Taekema, Angela Simpson, Aida Semic-Jusufagic, E. N. Clare Mills, Gourdon Dubois Nelly, Sellam Laetitia, Pereira Bruno, Michaud Elodie, Messaoudi Khaled, Evrard Bertrand, Fauquert Jean-Luc, Richard E. Goodman, Elena Rodríguez Plata, Luis Amaral, Borja Bartolomé, Alice Coimbra, Jose L Placido, Carmen Saviana Ganea, Carol Ann Costello, Martin Sorensen, Clare Mills, Adrian Rogers, Aage Otherhals, Tanja Kalic, Isabella Ellinger, Eva Waltl, Verena Niederberger-Leppin, Dawid Szczepankiewicz, Ewa Pruszynska-Oszmalek, Marek Skrzypski, Krzysztof W. Nowak, Aleksandra Szczepankiewicz, Gwang-Cheon Jang, Iva Markovic, Andreas Borowski, Tina Vetter, Andreas Wohlmann, Michael Kuepper, Karlheinz Friedrich, Ibon Eguiluz Gracia, Anthony Bosco, Ralph Dollner, Guro Reinholt Melum, Anya C Jones, Maria Lexberg, Patrick G Holt, Espen Sønderaal Bækkevold, Frode Lars Jahnsen, Paulina Sobkowiak, Marta Rachel, Beata Narozna, Dorota Jenerowicz, Witold Swiatowy, Anna Breborowicz, Reinhard Nestelbacher, Hiroyuki Fukui

**Affiliations:** 1 Luxembourg Institute of Health, Esch-Sur-Alzette, Luxembourg; 2 Eberhard Karls Universität, Tübingen, Germany; 3 Centre Hospitalier, Luxembourg, Luxembourg; 4 Technische Universität, München, Germany; 5 University of Salzburg, Christian Doppler Laboratory for Innovative Tools for Biosimilar Characterization, Salzburg, Austria; 6 Department of Molecular Biology, University of Salzburg, Salzburg, Austria; 7 Department of Dermatology, Paracelsus Medical University Salzburg, Salzburg, Austria; 8 Biochemistry and Molecular Biology I Department, Complutense University of Madrid, Madrid, Spain; 9 Thermo Fisher Scientific Inc., ImmunoDiagnostics Division, Uppsala, Sweden; 10 STALLERGENES, Antony, France; 11 University of Sao Paulo, Sao Paulo, Brazil; 12 University of Sao Paulo State (UNESP), Rio Claro, Brazil; 13 University of Salzburg, Salzburg, Austria; 14 University of Antwerp, Antwerp, Belgium; 15 Plant Biotechnology Institute, Madrid, Spain; 16 Abionic SA, Lausanne, Switzerland; 17 Department of Pathophysiology and Allergy Research, Medical University of Vienna, Vienna, Austria; 18 Department of Dermatology and Allergology, Philipps University Marburg, Marburg, Germany; 19 Department of Pathophysiology and Allergy Research & Christian Doppler Laboratory for Immunomodulation, Medical University of Vienna, Vienna, Austria; 20 Laboratory of Clinical Immunology and Allergy-LIM60, Division of Clinical Immunology and Allergy, Department of Medicine, School of Medicinea, University of Sao Paulo, Sao Paulo, Brazil; 21 Division of Allergy, Clinics Hospital of School of Medicine, University of Sao Paulo, Sao Paulo, Brazil; 22 Laboratory of Clinical Immunology and Allergy-LIM60, Division of Clinical Immunology and Allergy, Department of Medicine, School of Medicine, University of Sao Paulo, Sao Paulo, Brazil; 23 Institute of Biosciences of Rio Claro, University of Sao Paulo State (UNESP), Rio Claro, Brazil; 24 Division of Allergy, Clinics Hospital of School of Medicine, University of Sao Pauloa, Sao Paulo, Brazil; 25 Laboratory of Immunology, Heart Institute (InCor), LIM1 University of Sao Paulo, School of Medicine, Sao Paulo, Brazil; 26 Department of Molecular Biology, Division of Allergy and Immunology, University of Salzburg, Sao Paulo, Austria; 27 Department of Molecular Biology, Division of Allergy and Immunology, University of Salzburg, Salzburg, Brazil; 28 Laboratory of Clinical Immunology and Allergy-LIM60, Division of Clinical Immunology and Allergy, Department of Medicine, School of Medicine, University of Sao Paulo, Rio Claro, Brazil; 29 Division of Clinical and Molecular Allergology, Research Center Borstel, Airway Research Center North (ARCN), Member of the German Center for Lung Research (DZL), Borstel, Germany; 30 Core Facility Fluorescence Cytometry, Research Center Borstel, Borstel, Germany; 31 Interdisciplinary Allergy Outpatient Clinic, Department of Pneumology, University of Luebeck, Luebeck, Germany; 32 Pediatric Dept, Unit of Allergy and Immunology in Evolutive Age, Department of Clinical and Experimental Medicine, University of Parma, Parma, Italy; 33 Pediatric Dept and Pediatric Allergology Unit, Sandro Pertini Hospital, Rome, Italy; 34 Allergology Service, San Carlo Clinic, Paderno Dugnano, Italy; 35 Pediatric Unit, Dept. for Mother and Child, Ramazzini Hospital, Carpi, Italy; 36 Pediatric Unit, Department of Medical and Surgical Sciences, University of Bologna, Bologna, Italy; 37 Operative Complex Unit of Pediatrics and Neonatal Patology, Mazzoni Hospital, Ascoli Piceno, Italy; 38 Pediatric Department, La Sapienza University, Rome, Italy; 39 Azienda Sanitaria Locale Salerno, Salerno, Italy; 40 Pediatric Unit, Fatebenefratelli Hospital, Benevento, Italy; 41 Pediatric Section, Dept of Life and Reproduction Sciences, University of Verona, Verona, Italy; 42 Pediatric Unit, Grassi Hospital, Rome, Italy; 43 Pediatric Unit, San Giuseppe Hospital, Empoli, Italy; 44 Department of Pediatric Pneumology and Immunology, Charité Medical University, Berlin, Germany; 45 Allergieambulatorium Reumannplatz, Vienna, Austria; 46 Stallergenes SAS, Antony, France; 47 Sahlgrenska University Hospital, Goteborg, Sweden; 48 Immunoallergy Department, CUF Descobertas Hospital, Lisbon, Portugal; 49 Carol Davila University of Medicine and Pharmacy, Bucharest, Romania; 50 Nicolae Malaxa Clinical Hospital, Bucharest, Romania; 51 CHUC, Coimbra, Portugal; 52 University Hospital Nuestra Señora de Candelaria, Santa Cruz De Tenerife, Spain; 53 Clinical Hospital Nicolae Malaxa, Bucharest, Romania; 54 Department of Pediatric Pneumology & Immunology, Charité-Universitätsmedizin Berlin, Berlin, Germany; 55 Department of Clinical and Molecular Medicine, Sapienza University of Rome, S. Andrea University Hospital, Rome, Italy; 56 Department of Pediatrics, Technical University of Munich, Munich, Germany; 57 Department of Pediatrics St Hedwig, St Josefs Hospital, Freiburg, Germany; 58 Department of Pediatrics and Adolescent Medicine, University Medicine Mainz, Mainz, Germany; 59 Department of Pediatrics, University of Düsseldorf, Düsseldorf, Germany; 60 Department of Clinical and Molecular Medicine, Sapienza University of Rome, S. Andrea University Hospitalaa, Rome, Italy; 61 Institute of Social Medicine, Epidemiology and Health Economics, Charité-Universitätsmedizin Berlin, Berlin, Germany; 62 Laboratoire d’Immunologie, hôpital Rangueil, Toulouse, France; 63 Pneumo-Allergologie, hôpital Larrey, Toulouse, France; 64 Pneumo-Allergologie, hôpital des enfants, Toulouse, France; 65 Red Cross Blood Transfusion Service, Linz, Austria; 66 Biomay AG, Vienna, Austria; 67 Allergieambulatorium am Reumanplatz, Vienna, Austria; 68 School of Medicine, Kyorin University, Tokyo, Japan; 69 Graduate School of Medicine, Juntendo University, Tokyo, Japan; 70 Stallergens S.A., Antony, France; 71 Associated Centers for Molecular Allergology, Rome, Italy; 72 ZAUM, Center for Allergy and Environment, Munich, Germany; 73 Immunoallergology Department, Hospital Santa Maria - Centro Hospitalar Lisboa Norte, Lisboa, Portugal; 74 Salzburg University of Applied Sciences, Biomedical Sciences, Salzburg, Austria; 75 School of Education, University of Salzburg, Salzburg, Austria; 76 Department of Mathematics, University of Salzburg, Salzburg, Austria; 77 Medical Faculty, Charles University in Prague, Pilsen, Czech Republic; 78 National Institute of Public Health, Prague, Czech Republic; 79 Department of Paediatric Pneumology & Immunology, Charité-Universitätsmedizin Berlin, Berlin, Germany; 80 Department of Pediatrics, Technical University of Municha, Munich, Germany; 81 Department of Pediatrics and Adolescent Medicine, University Medicine Mainze, Mainz, Germany; 82 Department of Pediatrics, Heinrich-Heine-University, Düsseldorf, Germany; 83 Department of Paediatric Pneumology & Immunology, Altonaer Kinderkrankenhaus, Hamburg, Germany; 84 Institute for Social Medicine, Epidemiology and Health Economics, Charité - Universitätsmedizin Berlin, Berlin, Germany; 85 Division of Immunopathology, Department of Pathophysiology and Allergy Research, Center of Pathophysiology, Infectiology and Immunology, Medical University of Vienna, Vienna, Austria; 86 Immunoallergy Department, Hospital de Santa Maria – Centro Hospitalar Lisboa Norte, Lisbon, Portugal; 87 Faculdade de Medicina de Lisboa, Lisbon, Portugal; 88 Center of Allergy & Immunology, Tbilisi, Georgia; 89 Faculty of Medicine, Vilnius University, Vilnius, Lithuania; 90 Department of Mathematical Statistics, Vilnius Gediminas Technical University, Vilnius, Lithuania; 91 Public institution „Centro poliklinika“, Vilnius, Lithuania; 92 JSC „Imunodiagnostika“, Didzioji Riese, Lithuania; 93 Clinic of Pulmonology and Allergology, Vilnius University, Vilnius, Lithuania; 94 Public Institution, Vilnius, Lithuania; 95 Clinic of Pulmonology and Allergology, Vilnius University, Didzioji Riese, Lithuania; 96 Center of Allergy and Environment (ZAUM), Technical University of Munich and Helmholtz Center Munich, Munich, Germany; 97 Department of Dermatology and Allergy Biederstein, Technical University of Munich, Munich, Germany; 98 Hospital Universitario Reina Sofia, Córdoba, Córda, Spain; 99 Institute of Biochemistry and Molecular Biology, University of Hamburg, Hamburg, Germany; 100 Immunological Engineering, Department of Engineering, Aarhus University, Aarhus, Denmark; 101 Department of Infection and Immunity, Luxembourg Institute of Health (LIH), Esch-Sur-Alzette, Luxembourg; 102 Hopital Trousseau, Paris, France; 103 SFA hymenoptera task force, Lyon, France; 104 SFA hymenoptera task force, Toulouse, France; 105 SFA hymenoptera task force, Reims, France; 106 SFA hymenoptera task force, Aix-En-Provence, France; 107 Institut Pasteur, Paris, France; 108 Biomnis, Lyon, France; 109 Allergopharma GmbH & Co KG, Reinbek, Germany; 110 Merck KGaA, Darmstadt, Germany; 111 Aarhus University, Aarhus, Denmark; 112 University of Hamburg, Hamburg, Germany; 113 Euroimmun AG, Lübeck, Germany; 114 University Medical Center Freiburg, Freiburg, Germany; 115 Faculty of Chemistry, University of Belgrade, Belgrade, Serbia; 116 Karolinska Institutet, Stockholm, Sweden; 117 Faculty of Chemistry, University of Belgradea, Belgrade, Serbia; 118 Department of Internal Medicine, Södersjukhuset, Stockholm, Sweden; 119 Medical University of Vienna, Vienna, Austria; 120 The Jerzy Haber Institute of Catalysis and Surface Chemistry of the Polish Academy of Sciences, Krakow, Poland; 121 Avans University of Applied Science, School of Life Science and Environmental Technologies, Research group Analysis Techniques in Life Science, Breda, The Netherlands; 122 Wageningen UR Food & Biobased Research, Wageningen, The Netherlands; 123 Division of Allergology, Paul-Ehrlich Institut, Langen, Germany; 124 Allergieambulatorium, Vienna, Austria; 125 Institute of Organic Chemistry, Innsbruck, Austria; 126 Laboratorios Leti S.L.U., Madrid, Spain; 127 INRA UR1268 Biopolymers Interactions Assemblies, Nantes, France; 128 Allergy Clinic Reumannplatz, Vienna, Austria; 129 Yonsei University College of Medicine, Seoul, Korea; 130 Stallergenes S.A, Antony, France; 131 CSIR-Institute of Genomic and Integrative Biology, Delhi, India; 132 Paul-Ehrlich-Institut, Langen, Germany; 133 UR1268 BIA, INRA, Nantes, France; 134 USDA-ARS Western Regional Research Center, Albany, USA; 135 Unité d’Allergologie Générale et de Pneumologie, CHU d’Angers, Angers, France; 136 Service d’Allergologie, CH Epinal, Epinal, France; 137 Immunologie-Allergologie, CH de Luxembourg, Luxembourg, Luxembourg; 138 UR1268 BIA, Nantes, France; 139 CH Epinal, Epinal, France; 140 Hopital Necker, Paris, France; 141 CHU Angers, Angers, France; 142 INRA UR1268 BIA, Nantes, France; 143 University of Tuscia, Viterbo, Italy; 144 Faculté de Médecine de Nancy, Service d’Allergologie, Epinal, France; 145 Division of Immune Cell Analytics, Research Center Borstel, Borstel, Germany; 146 Division of Biophysics, Research Center Borstel, Borstel, Germany; 147 Chair of Animal Hygiene, Technische Universität München, Freising-Weihenstephan, Germany; 148 Division of Bioanalytical Chemistry, Research Center Borstel, Borstel, Germany; 149 Division of Clinical and Molecular Allergology, Research Center Borstel, Airway Research Center North (ARCN), Member of the German Center for Lung Research (DZL),Interdisciplinary Allergy Outpatient Clinic, MK III, University of Lübeck, Borstel/Lübeck, Germany; 150 University of Nebraska, Lincoln, NE USA; 151 HAL Allergy, Leiden, The Netherlands; 152 University of Belgrade, Belgrade, Serbia; 153 TNO, Zeist, The Netherlands; 154 North Carolina State University, Raleigh, NC USA; 155 USDA ARS, New Orleans, LA USA; 156 Department of Biochemistry and Molecular Biology, School of Chemistry, Complutense University of Madrid, Madrid, Spain; 157 Division of Clinical Immunology and Allergy, The Hospital for Sick Children, Toronto, Canada; 158 INRA UR 1268 BIA (Biopolymers, Interactions, Assemblies), Nantes, France; 159 University of Münster, Münster, Germany; 160 National Food Research Institute, National Agriculture and Food Research Organization, Tsukuba, Ibaraki, Japan; 161 National Institute of Health Sciences, Setagaya, Tokyo, Japan; 162 Department of Biotechnology, University of Natural Resources and Life Sciences, Vienna, Austria; 163 Molecular Biotechnology Section, University of Applied Sciences, FH Campus Wien, Campus Vienna Biocenter, Vienna, Austria; 164 Department of Biomedical Analytics, University of Applied Sciences Wiener Neustadt, Wiener Neustadt, Austria; 165 Division of Immunopathology, Department of Pathophysiology and Allergy Research, Center for Pathophysiology, Infectiology and Immunology, Medical University of Vienna, Vienna, Austria; 166 Department of Infection and Immunity, Luxembourg Institute of Health, Esch-Sur-Alzette, Luxembourg; 167 Department of Molecular Biologya, University of Salzburg, Salzburg, Austria; 168 Department of Dermatology, Paracelsus Private Medical University Salzburg, Salzburg, Austria; 169 Private practice, Carouge, Switzerland; 170 Children Cancer Research, Vienna, Austria; 171 Desentum Oy, Espoo, Finland; 172 VTT Technical Research Centre of Finland Ltd, Espoo, Finland; 173 Department of Chemistry, University of Eastern Finland, Joensuu, Finland; 174 Department of Molecular Biology, Division of Allergy and Immunology, University of Salzburg, Salzburg, Austria; 175 Christian Doppler Laboratory for Innovative Tools for the Characterization of Biosimilars, Salzburg, Austria; 176 Department of Internal Medicine, Division of Allergy and Immunology, Yonsei University College of Medicine, Seoul, Korea; 177 CSIR-Institute of Genomic and Integrative Biology, Allergy and Immunology Section, New Delhi, India; 178 Division of Allergology, Paul-Ehrlich-Institut, Langen, Germany; 179 University Clinic for Respiratory and Allergic Diseases Golnik, Golnik, Slovenia; 180 Immunoallergology’s Department, Hospital de Santa Maria, CHLN, E.P.E., Lisboa, Portugal; 181 Laboratory of Clinical Immunology, Faculdade de Medicina, Universidade de Lisboa. /Instituto de Medicina Molecular, Lisboa, Portugal; 182 Immunoallergology’s Department, Hospital de Santa Maria, CHLN, E.P.E, Lisboa, Portugal; 183 Immunology Laboratory, Centre Hospitalier Lyon Sud, Hospices Civils de Lyon, Pierre-Benite, France; 184 Genclis SA, Vandoeuvre-Lès-Nancy, France; 185 UMR152 IRD-UPS, Toulouse, France; 186 Immunology Laboratory, Hôpital de la Conception, Assistance Publique-Hôpitaux de Marseille, Marseille, France; 187 Metropolitan Hôpital de la Conception, Laboratoire d’Immunologie, Marseille, France; 188 Metropolitan Galenus Regeneratio, Tours, France; 189 Metropolitan, Hôpital de la Timone, Service de Pédiatrie Multidisciplinaire, Marseille, France; 190 Metropolitan, Service de Dermatologie-Vénéréologie, Hôpital de la Timone, Marseille, France; 191 Great North Children’s Hospital, Newcastle Upon Tyne, UK; 192 Royal Free Hospital, London, UK; 193 Royal National Throat Nose and Ear Hospital, London, UK; 194 Royal National Throat, Nose and Ear Hospital, London, UK; 195 Immunoallergy Department, Coimbra University Hospital Centre, Coimbra, Portugal; 196 Aarhus University Hospital, Aarhus, Denmark; 197 Odense University Hospital, Odense, Denmark; 198 Serviço de Imunoalergologia, Centro Hospitalar São João, Porto, Portugal; 199 Allergy and Clinical Immunology Department, Unidade Local de Saúde do Alto Minho, Viana Do Castelo, Portugal; 200 Immunology Lab, Department of Clinical Pathology, Centro Hospitalar São João, Porto, Portugal; 201 Laboratory of Immunology, Basic and Clinical Immunology Unit, Faculty of Medicine, Porto University, Porto, Portugal; 202 Hospital Dona Estefânia - Centro Hospitalar Lisboa Central, Lisbon, Portugal; 203 Centro Hospitalar de Setúbal, Setúbal, Portugal; 204 Great North Children’s Hospital, Newcastle, UK; 205 Institute of Inflammation and Repair, Manchester, UK; 206 University Hospital of South Manchester, Manchester, UK; 207 CHU, Clermont-Ferrand, France; 208 CH, Montlucon, France; 209 Dept. of Food Science & Technology, University of Nebraska-Lincoln, Lincoln, USA; 210 Bial Aristegui, Bilbao, Spain; 211 University of Manchester, Manchester, UK; 212 University Hospital of North Norway, Tromsoe, Norway; 213 Romer Labs, Runcorn, UK; 214 Nofima, Tromsoe, Norway; 215 Department of Otorhinolaryngology, Medical University of Vienna, Vienna, Austria; 216 Poznan University of Life Sciences, Poznan, Poland; 217 Poznan University of Medical Sciences, Poznan, Poland; 218 NHIS ILSAN Hospital, Ilsan, Korea; 219 Institute for Biochemistry II, University Hospital Jena, Jena, Germany; 220 Department of Pneumology, University of Rostock, Rostock, Germany; 221 Department of Pathology and Center for Immune Regulation, Oslo University Hospital-Rikshospitalet and University of Oslo, Oslo, Norway; 222 Telethon Kids Institute, The University of Western Australia, Perth, WA Australia; 223 Department of Otorhinolaringology, Head and Neck Surgery, Oslo University Hospital-Rikshospitalet, Oslo, Norway; 224 Department of Allergy, Provincial Hospital No Rzeszów, Rzeszów, Poland; 225 DNA-Consult Sciencetainment, Ostermiething, Austria; 226 Tokushima University, Tokushima, Japan

## Abstract

ORAL ABSTRACTS

Symposium 1: Biochemistry, structure and environment of the allergen: what makes a protein an allergen?

O1 Two cell-membrane peptidases carrying galactose-alpha-1,3-galactose are implicated in delayed anaphylactic reactions upon pork kidney ingestion in patients with IgE-antibodies to alpha-Gal

Christiane Hilger, Kyra Swiontek, Jörg Fischer, François Hentges, Christiane Lehners, Martine Morisset, Bernadette Eberlein, Tilo Biedermann, Markus Ollert

O2 Structure solution of Pla l 1 suggests similar folding of Ole e 1-like family members but distinct immunological properties

Sabrina Wildner, Teresa Stemeseder, Regina Freier, Peter Briza, Roland Lang, Eva Batanero, Mayte Villalba, Jonas Lidholm, Thomas Hawranek, Fatima Ferreira, Hans Brandstetter, Gabriele Gadermaier

Symposium 2: New allergen molecules in the spotlight

O3 Identification of the cysteine protease Amb a 11 as a novel major allergen from short ragweed (*Ambrosia artemisiifolia*)

Philippe Moingeon, Rachel Groeme, Julien Bouley, Véronique Bordas, Maxime Le Mignon, Laetitia Bussières, Aurélie Lautrette, Laurent Mascarell, Vincent Lombardi, Véronique Baron-Bodo, Henri Chabre, Thierry Batard, Emmanuel Nony

O4 Production and characterization of polybia paulista recombinant antigen 5: a valuable diagnostic tool

Karine Marafigo De Amicis, Alexandra Sayuri Watanabe, Daniele Danella Figo, José Roberto Aparecido Dos Santos-Pinto, Mario Sergio Palma, Fabio Fernandes Morato Castro, Jorge Kalil, Therese Wohlschlager, Peter Briza, Sabrina Wildner, Fatima Ferreira-Briza, Gabriele Gadermaier, Keity Souza Santos

Symposium 3: Progress in molecular and cellular diagnosis

O5 Basophil activation test with recombinant Pru p 3; identifying genuine peach allergic patients

Margaretha Faber, Athina Van Gasse, Vito Sabato, Margo M. Hagendorens, Chris H. Bridts, Luc S. De Clerck, Araceli Diaz Perales, Didier Ebo

O6 Nanofluidic technology enables rapid, near-patient quantification of allergen-specific IgE

Petra Zavadakova, Aurélie Buchwalder, Fabien Rebeaud, Iwan Märki

Symposium 4: Relevance of molecular diagnostics for intervention and treatment

O7 Longitudinal analysis of Bet v 1-specific epitope repertoires during birch pollen immunotherapy

Barbara Gepp, Nina Lengger, Christian Möbs, Wolfgang Pfützner, Christian Radauer, Barbara Bohle

O8 A natural CCD-free tool: is polistes sp. venom suitable for polybia paulista diagnosis and therapy?

Karine Marafigo De Amicis, Alexandra Sayuri Watanabe, Clovis Eduardo Galvao, Daniele Danella Figo, Jose Roberto Aparecido Santos-Pinto, Mario Sergio Palma, Fabio Fernandes Morato Castro, Jorge Kalil, Fatima Ferreira, Gabriele Gadermaier, Keity Souza Santos

Symposium 5: The advent of molecular allergology in epidemiology

O9 Peanut oleosins: from identification to diagnostic testing

Christian Schwager, Skadi Kull, Frauke Schocker, Jochen Behrends, Wolf-Meinhard Becker, Uta Jappe

O10 Endotypes of oral allergy syndrome in childhood: a molecular diagnostic approach

Carla Mastrorilli, Salvatore Tripodi, Carlo Caffarelli, Riccardo Asero, Arianna Dondi, Giampaolo Ricci, Carlotta Povesi Dascola, Elisabetta Calamelli, Andrea Di Rienzo Businco, Annamaria Bianchi, Tullio Frediani, Carmen Verga, Iride Dello Iacono, Diego Peroni, Giuseppe Pingitore, Roberto Bernardini, Paolo Maria Matricardi

Symposium 6: Molecular AIT: which approaches will make it to market?

O11 Mbc4: an innovative molecule to tackle birch pollen and concomitant food allergies

Heidi Hofer, Claudia Asam, Michael Hauser, Peter Briza, Martin Himly, Christof Ebner, Fatima Ferreira

O12 Challenges and solutions associated with the production of recombinant Bet v 1 allergen as a therapeutic protein

Emmanuel Nony, Maxime Le Mignon, Pierrick Lemoine, Karine Jain, Kathy Abiteboul, Monica Arvidsson, Sabina Rak, Philippe Moingeon

Clinical Cases: Breakthroughs and headaches from CRD: interactive session

CC1 Anaphylaxis caused by lipid transfer proteins: a complex clinical pattern syndrome

Inês Mota, Filipe Benito Garcia, Angela Gaspar, Cristina Arêde, Susana Piedade, Graça Sampaio, Graça Pires, Luís Miguel Borrego, Cristina Santa-Marta, Mário Morais-Almeida

CC2 IgE sensitization profile in a patient with asteraceae pollen-exotic fruits association

Florin-Dan Popescu, Mariana Vieru, Florin-Adrian Secureanu

CC3 Food-dependent: exercise induced anaphylaxis. Which component to blame?

Rosa Anita Rodrigues Fernandes, Isabel Carrapatoso, Raquel Gomes, Celso Pereira, Ana Todo-Bom

CC4 Anaphylaxis to intravenous iron preparations in a patient that tolerates oral administration

María Cecilia Martín Fernández De Basoa, Javier Barrios Regio, Juan De Castro Cordova, Antón Fernández Ferreiro

CC5 IgE sensitization pattern in an adult patient with oral allergy syndrome to peanuts and pollinosis from southern Romania

Florin-Dan Popescu, Mariana Vieru, Florin-Adrian Secureanu

CC6 Evidence of specific IgE to plant-derived cross-reactive carbohydrate determinant in a patient with delayed anaphylaxis to red meat

Mariana Vieru, Florin-Dan Popescu, Florin-Adrian Secureanu

POSTER PRESENTATIONS

Poster Session 1: Molecular allergology and epidemiology

P1 Atopic children produce stronger and more frequent IgG responses than non-atopic children: longitudinal data from the German MAS birth cohort

Olympia Tsilochristou, Serena Perna, Alina Schwarz, Alexander Rohrbach, Antonio Cappella, Laura Hatzler, Carl-Peter Bauer, Ute Hoffmann, Johannes Forster, Fred Zepp, Antje Schuster, Raffael D’amelio, Ulrich Wahn, Thomas Keil, Susanne Lau, Paolo Maria Matricardi

P2 The IgG sensitization profiles against 112 allergenic components support the absence of a protective role of IgG in allergic individuals, outside of the context of SIT

Pol André Apoil, Claire Mailhol, Anne Broué-Chabbert, Agnès Juchet, Alain Didier, Elodie Carrer, Thomas Lanot, Antoine Blancher

P3 The immune response against the timothy grass pollen allergen Phl p 5 in non-allergic humans

Almedina Kurtaj, Christoph Hillebrand, Gerda Fichtinger, Martin Danzer, Christian Gabriel, Theresa Thalhamer, Sandra Scheiblhofer, Josef Thalhamer, Richard Weiss

P4 Analyzing the cross-reactivity profile of the major ragweed allergen Amb a 1

Martin Wolf, Michael Hauser, Ulrike Pichler, Teresa Twaroch, Gabriele Gadermaier, Christof Ebner, Hidenori Yokoi, Toshiro Takai, Alain Didierlaurent, Adriano Mari, Peter Briza, Heidrun Behrendt, Angela Neubauer, Frank Stolz, Fátima Ferreira, Michael Wallner

P5 LTP (Pru p 3) sensitisation in skin prick test: which means in clinical practice?

Sara Carvalho, Tatiana Lourenço, Joana Cosme, Fátima Cabral Duarte, Amélia Spínola Santos, Ana Célia Costa, Manuel Pereira Barbosa

P6 IgE profiles, allergen exposure and lifestyle of 501 Austrian pupils: investigation of influences on the development of allergic sensitizations

Teresa Stemeseder, Eva Klinglmayr, Bettina Schweidler, Lisa Lueftenegger, Stephanie Moser, Patrick Doppler, Roland Lang, Martin Himly, Gertie J. Oostingh, Arne Bathke, Joerg Zumbach, Thomas Hawranek, Gabriele Gadermaier

P7 Molecular profiles of sensitization to perennial inhalant allergens in a middle European region

Petr Panzner, Martina Vachova, Tomas Vlas, Marek Maly

P8 Evolution of the IgE response to house dust mite allergen molecules in childhood

Daniela Posa, Serena Perna, Stephanie Hofmaier, Laura Hatzler, Alexander Rohrbach, Carl-Peter Bauer, Ute Hoffmann, Johannes Forster, Fred Zepp, Antje Schuster, Philippe Stock, Ulrich Wahn, Linus Grabenhenrich, Thomas Keil, Susanne Lau, Kuan-Wei Chen, Yvonne Resch, Susanne Vrtala, Rudolf Valenta, Paolo Maria Matricardi

P9 Tropomyosin (Pen a1): to include or not to include in skin prick testing?

Joana Cosme, Sara Carvalho, Tatiana Lourenço, Amélia Spínola Santos, Manuel Pereira Barbosa

Immunoallergy Department - Hospital de Santa Maria – Centro Hospitalar Lisboa Norte, Lisbon, Portugal, Lisbon, Portugal; Immunoallergy Department - Hospital de Santa Maria – Centro Hospitalar Lisboa Norte, Lisbon, Portugal; Faculdade de Medicina de Lisboa, Lisbon, Portugal

P10 Component-resolved IgE profiles in Georgian patients

Tamar Abramidze, Nino Lomidze, Maia Gotua

P11 Cross reactivity between food and pollen allergens in Lithuania according to spIgE evaluation

Austeja Dapkeviciute, Ruta Einikyte, Jolita Norkuniene, Laima Skrickiene, Asta Miskiniene, Violeta Kvedariene

P12 Distribution of inhalant allergy in the population of Lithuania

Ruta Einikyte, Austeja Dapkeviciute, Jolita Norkuniene, Laima Skrickiene, Asta Miskiniene, Violeta Kvedariene

Poster Session 2: Allergen molecules: identification, characterization, structure and function

P13 Interference of antigen 5-based cross-reactivity in the diagnosis of hymenoptera venom allergy

Maximilian Schiener, Bernadette Eberlein, Carmen Moreno-Aguilar, Gunilla Pietsch, Mareike Mc Intyre, Lea Schwarze, Dennis Rußkamp, Tilo Biedermann, Edzard Spillner, Ulf Darsow, Carsten Schmidt-Weber, Markus Ollert, Simon Blank

P14 IgE cross-reactivity between European Hymenoptera and Asian hornet (*Vespa velutina*) venom allergens

Cyril Longé, Andrea Brazdova, Jean-Louis Brunet, Claire Schwartz, Bruno Girodet, François Lavaud, Joelle Birnbaum, Nhân Pham Thi, Magalie Duchateau, Julia Chamot-Rooke, Laurence Guilloux, Marie-Ange Selva, Rémy Couderc, Hélène Sénéchal, Jean-Pierre Sutra, Pascal Poncet

P15 Carbohydrate composition of house dust mite extracts and major group 1 and group 2 allergens

Steffen Augustin, Linda Pump, Martin Wald, Thomas Eichhorn, Frank Fischer, Christoph Willers

P16 Specificity of monoclonal antibodies against cross-reactive carbohydrate determinants

Michaela Miehe, Melanie Plum, Sara Wolf, Frederic Jabs, Tim Raiber, Frank Bantleon, Henning Seismann, Thilo Jakob, Edzard Spillner

P17 Red meat allergic patients have a selective IgE response to the a-Gal glycan

Danijela Apostolovic, Anh Thu Tran, Sara Sanchez-Vidaurre, Tanja Cirkovic Velickovic, Maria Starkhammar, Carl Hamsten, Marianne Van Hage

P18 Specificity of non-specific lipid transfer proteins and influence of the ligands on their three-dimensional structure

Pawel Dubiela, Piotr Humeniuk, Sabine Pfeifer, Merima Bublin, Tomasz Borowski, Karin Hoffmann-Sommergruber

P19 Real-time PCR analysis of Pru av 1 and Pru av 3 allergens

Martie C.M. Verschuren, Shanna Bastiaan-Net, Defien Depoortere, Kay Foetisch, Stephan Scheurer, Harry J Wichers, Theo Noij

P20 Specificity of anti-Pru av 1 antibodies for the detection of Pru av 1 isoallergens

Martie C.M. Verschuren, Shanna Bastiaan-Net, Nikki M.E. Van Uden, Karel Vandenberghe, Kay Foetisch, Stephan Scheurer, Harry J. Wichers H.J., Theo H.M. Noij

P21 Enhancing recombinant production yield of Bet v 1 through codon usage harmonization

Anargyros Roulias, Maria Alejandra Parigiani, Heidi Hofer, Claudia Asam, Christof Ebner, Fátima Ferreira

P22 Structural and dynamic insights into the world of PR-10 allergens

Linda Ahammer, Sarina Grutsch, Martin Tollinger

Poster Session 3: Allergen molecules: identification, characterization, structure and function

P23 Purification of polcalcin from different pollen allergenic sources by antibody-affinity chromatography

Raquel Moya, Mª Angeles López-Matas, Raquel Reyes, Jerónimo Carnés

P24 Variations of wheat allergens in cultivars measured through a targeted quantitative mass spectrometry approach

Colette Larré, Hélène Rogniaux, Roberta Lupi, Sandra Denery-Papini

P25 Art v 1, Amb a 4 and Par h 1 defensin-like proteins share similar structural features but distinct immunological and allergenic properties

Isabel Maria Pablos, Stephanie Eichhorn, Yoan Machado, Peter Briza, Christof Ebner, Jung-Won Park, Alain Didierlaurent, Naveen Arora, Stefan Vieths, Gabriele Gadermaier, Fatima Ferreira

P26 Homogeneity or diversity of IgE-binding proteins in wheat dependant exercise induced anaphylaxis?

Sandra Denery-Papini, Charlene Tanaka, Florence Pineau, Roberta Lupi, Martine Drouet, Etienne Beaudouin, Martine Morisset, Susan Altenbach

P27 Deciphering the role of disulfide bonds and of repetitive epitopes in immunoglobulin E binding to wheat gliadins

Sandra Denery-Papini, Hamza Mameri, Chantal Brossard, Roberta Lupi, Florence Pineau, Jean Charles Gaudin, Denise Anne Moneret-Vautrin, Etienne Beaudouin, Evelyne Paty, Martine Drouet, Olivier Tranquet, Colette Larré

P28 Assessment of the allergenicity of soluble fractions from bread and durum wheats genotypes

Roberta Lupi, Stefania Masci, Olivier Tranquet, Denise-Anne Moneret-Vautrin, Sandra Denery-Papini, Colette Larré

P29 Isolation and characterization of Ara h 12 and Ara h 13: defensins, a novel class of peanut allergens

Skadi Kull, Arnd Petersen, Marisa Böttger, Sandra Rennert, Wolf-Meinhard Becker, Susanne Krause, Martin Ernst, Thomas Gutsmann, Johann Bauer, Buko Lindner, Uta Jappe

P30 Allergenicity attributes of different peanut market types

Stef Koppelman, Shyamali Jayasena, Dion Luykx, Erik Schepens, Danijela Apostolovic, Govardus De Jong, Tom Isleib, Julie Nordlee, Joe Baumert, Steve Taylor, Soheila Maleki

P31 The impact of peanut lipids on Ara h 1-induced immune responses in monocytes-derived dendritic cells

Chiara Palladino, Barbara Gepp, Sofía Sirvent, Alba Angelina, Merima Bublin, Christian Radauer, Nina Lengger, Thomas Eiwegger, Oscar Palomares, Heimo Breiteneder

P32 Compared allergenicity of native and thermally aggregated ovalbumin as large agglomerated particles

Mathilde Claude, Roberta Lupi, Grégory Bouchaud, Marie Bodinier, Chantal Brossard, Sandra Denery-Papini

P33 Simulation of the gastrointestinal digestion of the hazelnut allergens Cor a 9 and Cor a 11 by an in-vitro model and characterisation of peptidic products including epitopes by HPLC-MS/MS

Robin Korte, Julia Bräcker, Jens Brockmeyer

P34 Analysis of distribution of rice allergens in brown rice grain and allergenicity of the products containing rice bran

Rie Satoh, Reiko Teshima

Poster Session 4: Molecular approaches in AIT

P35 Production of a recombinant hypoallergenic variant of the major peanut allergen Ara h 2 for allergen-specific immunotherapy

Angelika Tscheppe, Dieter Palmberger, Merima Bublin, Christian Radauer, Chiara Palladino, Barbara Gepp, Nina Lengger, Reingard Grabherr, Heimo Breiteneder

P36 Mutagenesis of amino acids critical for calcium-binding leads to the generation of a hypoallergenic Phl p 7 variant

Marianne Raith, Linda Sonnleitner, Doris Zach, Konrad Woroszylo, Margit Focke-Tejkl, Herbert Wank, Thorsten Graf, Annette Kuehn, Ines Swoboda

P37 Are birch pollen allergen immunotherapy induced blocking antibodies protective for cross-reactive allergens?

Claudia Asam, Sara Huber, Heidi Hofer, Roland Lang, Thomas Hawranek, Fátima Ferreira, Michael Wallner

P38 High success of 58 subcutaneous immunotherapy for pets allergy in a polyallergic cohort of patients: a component resolved individually adapted treatment (CRIAT)

Fabienne Gay-Crosier

P39 Neutrophils are potential antigen presenting cells in IgE- mediated allergy

Dominika Polak, Birgit Nagl, Claudia Kitzmüller, Barbara Bohle

P40 Characterization of allergen-specific CD8+ T cells in type I allergy

Nazanin Samadi, Claudia Kitzmüller, Rene Geyeregger, Barbara Bohle, Beatrice Jahn-Schmid

Poster Session 5: Molecular and cellular diagnostic tests

P41 Nanofluidic-based biosensors allow quantification of total circulating IgE from a drop of blood in 5 minutes

Aurélie Buchwalder, Ariel Gomez, Fabien Rebeaud, Iwan Märki

P42 Allergen microarray for the analysis of serum IgE binding profile and allergenic activity

Jaana Haka, Liisa Hattara, Marika Heikkinen, Merja H Niemi, Juha Rouvinen, Petri Saviranta, Pekka Mattila, Kristiina Takkinen, Marja-Leena Laukkanen

P43 Generation of a well-characterized panel of periplaneta americana allergens for component resolved diagnosis

Stephanie Eichhorn, Isabel Pablos, Bianca Kastner, Bettina Schweidler, Sabrina Wildner, Peter Briza, Jung-Won Park, Naveen Arora, Stefan Vieths, Gabriele Gadermaier, Fatima Ferreira

P44 Improved diagnostic sensitivity of recombinant Api m 1 and Ves v 5 in diagnosis of Hymenoptera venom allergy

Mira Silar, Julij Selb, Rok Kogovsek, Mitja Kosnik, Peter Korosec

P45 Added value of biomarkers of primary sensitization and cross-reactivity in patients with hymenoptera venom allergy

Leticia Pestana, Alcinda Campos Melo, Ana Mendes, Maria Elisa Pedro, Manuel Pereira Barbosa, Maria Conceição Pereira Santos

P46 Cosensitization to Alt a 1 and Act d 2: more than a fortuitous association?

Françoise Bienvenu, Claire Goursaud, Lorna Garnier, Sandrine Jacquenet, Michaël Degaud, Sébastien Viel, Annick Barre, Pierre Rougé, Jacques Bienvenu, Joana Vitte

P47 Molecular diagnosis for peanut allergy: ALFA method performs as well as established methods for Ara h 1, Ara h 2, Ara h 6, Ara h 9 and CCD

Amel Bensalah, Isabelle Cleach, Laurent Mousseau, Chantal Agabriel, Valérie Liabeuf, Joëlle Birnbaum, Jean-Louis Mège, Joana Vitte

P48 Evaluation of a food challenge service in relation to specific IgE to molecular components in children with suspected peanut allergy

James Gardner, Minal Gandhi, Harsha Kariyawasam, Giuseppina Rotiroti

P49 Component resolved diagnosis in cereal allergy

Isabel Carrapatoso, Celso Pereira, Frederico Regateiro, Emília Faria, Ana Todo-Bom

Poster Session 6: Molecular diagnosis in prevention and therapy

P50 Pretreatment molecular sensitizations determine the sIgG4 induction during the updosing of SCIT and may be useful to identify clinically relevant additional sensitizations

Johannes Martin Schmid, Ronald Dahl, Hans Juergen Hoffmann

P51 Usefulness of recombinant latex allergens in immunotherapy’s decision and follow-up

Inês Mota, Filipe Benito Garcia, Angela Gaspar, Mário Morais-Almeida

P52 Omega-5-gliadin in the diagnosis of wheat-dependent anaphylaxis induced by ibuprofen but not by exercise

Joana Cosme, Letícia Pestana, Amélia Spínola Santos, Manuel Pereira Barbosa

P53 Food dependent exercise-induced anaphylaxis: a component-resolved and in vitro depletion approach to access IgE cross-reactivity

Diana Silva, Teresa Vieira, Ana Maria Pereira, André Moreira, Luís Delgado

P54 Olive pollen allergens: what are we missing?

Sara Prates, Cátia Alves, Elena Finelli, Paula Leiria Pinto

P55 Purified Alt a 1 extract in *Alternaria alternata* allergy diagnosis

Bárbara Kong Cardoso, Cíntia Cruz, Filipa Semedo, Elza Tomaz, Filipe Inácio

P56 Use of specific IgE Bos d8 (casein) to aid early introduction of dietary baked milk in children with cows’ milk allergy

James Gardner, Santanu Maity, Giuseppina Rotiroti, Minal Gandhi

P57 Molecular characterisation and immunoreactivity of a peanut ingredient for use in oral food challenges

Ivona Baricevic-Jones, Justin T. Marsh, Phil E. Johnson, Anuradha Balasundaram, Anya-May Hope, Aafke Taekema, Angela Simpson, Aida Semic-Jusufagic, E.N. Clare Mills

P58 Specific IgE to recombinant allergens of hazelnut and oral food challenge in children

Gourdon Dubois Nelly, Sellam Laetitia, Pereira Bruno, Michaud Elodie, Messaoudi Khaled, Evrard Bertrand, Fauquert Jean-Luc

Poster session 7/8: miscellaneous

P59 What defines a protein as an allergen? A discussion of sources and sufficiency

Richard E. Goodman

P60 Cat allergy: relationship between clinical and molecular diagnostic

María Cecilia Martín Fernández De Basoa, Antón Fernández Ferreiro, Elena Rodríguez Plata

P61 Anaphylaxis to rabbit: the cat came in last

Luis Amaral, Borja Bartolomé, Alice Coimbra, Jose L Placido

P62 Dog allergy: relationship between clinical and molecular diagnostic

María Cecilia Martín Fernández De Basoa, Antón Fernández Ferreiro, Elena Rodríguez Plata

P63 Correlation of serum timothy grass-pollen specific IgE levels determined by two immunoblot test systems

Mariana Vieru, Florin-Dan Popescu, Florin-Adrian Secureanu, Carmen Saviana Ganea

P64 Development of oral food challenge formulations for diagnosis of fish allergy using powdered fish ingredients

Carol Ann Costello, Ivona Baricevic-Jones, Martin Sorensen, Clare Mills, Adrian Rogers, Aage Otherhals

P65 Fish and peanut allergens interact with plasma membranes of intestinal and bronchial epithelial cells and induce differential gene expression of cytokines and chemokines

Tanja Kalic, Isabella Ellinger, Chiara Palladino, Barbara Gepp, Eva Waltl, Verena Niederberger-Leppin, Heimo Breiteneder

P66 Interleukin 4 affects fat tissue metabolism and expression of pro-inflammatory factors in isolated rat adipocytes

Dawid Szczepankiewicz, Ewa Pruszynska-Oszmalek, Marek Skrzypski, Krzysztof W. Nowak, Aleksandra Szczepankiewicz

P67 Ozone induced airway hyperreactivity in PD-L2−/− mice model

Gwang-Cheon Jang

P68 Thymic stromal lymphopoietin (TSLP) and its receptor as targets for the development of anti-inflammatory inhibitory agents

Iva Markovic, Andreas Borowski, Tina Vetter, Andreas Wohlmann, Michael Kuepper, Karlheinz Friedrich

P69 The mononuclear phagocyte system in experimentally-induced allergic rhinitis

Ibon Eguiluz Gracia, Anthony Bosco, Ralph Dollner, Guro Reinholt Melum, Anya C Jones, Maria Lexberg, Patrick G Holt, Espen Sønderaal Bækkevold, Frode Lars Jahnsen

P70 Expression of histamine metabolizing enzymes is increased in allergic children

Aleksandra Szczepankiewicz, Paulina Sobkowiak, Marta Rachel, Beata Narozna, Dorota Jenerowicz, Witold Swiatowy, Anna Breborowicz

P71 Modifying the glycosylation of human IgE towards oligomannosidic structures does not affect its biological activity

Melanie Plum, Sara Wolf, Frank Bantleon, Henning Seismann, Frederic Jabs, Michaela Miehe, Thilo Jakob, Edzard Spillner

P72 Flying Labs: an educational initiative to transfer allergy research into high-school settings

Michael Wallner, Heidi Hofer, Fatima Ferreira, Reinhard Nestelbacher

P73 Clinical significance of antihistamines and Kujin, an anti-allergic Kampo medicine

Hiroyuki Fukui

## ORAL ABSTRACTS

### Symposium 1: Biochemistry, structure and environment of the allergen: what makes a protein an allergen?

#### O1 Two cell-membrane peptidases carrying galactose-alpha-1,3-galactose are implicated in delayed anaphylactic reactions upon pork kidney ingestion in patients with IgE-antibodies to alpha-Gal

##### Christiane Hilger^1^, Kyra Swiontek^1^, Jörg Fischer^2^, François Hentges^3^, Christiane Lehners^3^, Martine Morisset^3^, Bernadette Eberlein^4^, Tilo Biedermann^4^, Markus Ollert^1^

###### ^1^Luxembourg Institute of Health, Esch-Sur-Alzette, Luxembourg; ^2^Eberhard Karls Universität, Tübingen, Germany; ^3^Centre Hospitalier, Luxembourg, Luxembourg; ^4^Technische Universität, München, Germany

**Correspondence**: Christiane Hilger

*Clinical and Translational Allergy* 2016, **6(Suppl 2)**:O1

**Background**: Delayed food anaphylaxis upon consumption of red meat is attributed to specific IgE-antibodies directed to galactose-α-1,3-galactose (α-Gal). Anaphylactic reactions may occur after ingestion of meat from different mammals, mainly beef and pork, but reactions to lamb, rabbit or horse have also been reported. In particular, pork kidney has been shown to trigger symptoms that were more severe and occurred within a shorter delay.

The objective of the present study was the identification and characterization of pork kidney proteins carrying α-Gal carbohydrates and mediating delayed allergic reactions through specific IgE to α-Gal.

**Materials and methods**: A cohort of 59 patients with specific IgE to α-Gal was screened by immunoblot for IgE-reactive proteins in pork kidney extract. Proteins were purified by affinity chromatography and identified by Edman sequencing and peptide mass fingerprinting. Isolated proteins were used in immunoassays using patient sera and α-Gal specific antibodies. Allergenicity was assayed in basophil activation and skin prick test.

**Results**: Multiple IgE-binding proteins were detected in protein extracts of pork kidney by immunoblot using patient sera and an anti-α-Gal antibody. Reactive bands were located in the high molecular weight range of 100 to ≥200 kDa. Two major IgE-binding proteins were identified as porcine angiotensin I converting enzyme (ACE I) and aminopeptidase N (AP-N). IgE-binding to both proteins was lost by periodate treatment, resulting in oxidation of carbohydrates. Addition of α-Gal inhibited IgE-reactivity to both peptidases. Allergenicity was confirmed by activation of patient basophils and positive skin prick tests.

**Conclusions**: Two IgE-reactive cell membrane peptidases carrying α-Gal epitopes were identified in pork kidney, a tissue which is known as potent inducer of red meat-induced anaphylaxis. Allergenicity and clinical relevance of these proteins were confirmed in patients with delayed anaphylaxis to red meat by skin prick test and basophil activation.

#### O2 Structure solution of Pla l 1 suggests similar folding of Ole e 1-like family members but distinct immunological properties

##### Sabrina Wildner^1^, Teresa Stemeseder^2^, Regina Freier^2^, Peter Briza^2^, Roland Lang^3^, Eva Batanero^4^, Mayte Villalba^4^, Jonas Lidholm^5^, Thomas Hawranek^3^, Fatima Ferreira^2^, Hans Brandstetter^2^, Gabriele Gadermaier^1^

###### ^1^University of Salzburg, Christian Doppler Laboratory for Innovative Tools for Biosimilar Characterization, Salzburg, Austria; ^2^University of Salzburg, Department of Molecular Biology, Salzburg, Austria; ^3^Paracelsus Medical University Salzburg, Department of Dermatology, Salzburg, Austria; ^4^Complutense University of Madrid, Biochemistry and Molecular Biology I Department, Madrid, Spain; ^5^Thermo Fisher Scientific Inc., ImmunoDiagnostics Division, Uppsala, Sweden

**Correspondence**: Sabrina Wildner

*Clinical and Translational Allergy* 2016, **6(Suppl 2)**: O2

**Background**: Pollen of English plantain (*Plantago lanceolata*) has been associated with hay fever and asthma from late spring to autumn in temperate regions of the Northern hemisphere. Pla l 1, the major allergen from plantain, is a glycoprotein which belongs to the Ole e 1-like protein family. Therefore, we set out to solve the structure of Pla l 1 and compare its immunological properties with other allergenic Ole e 1-like proteins.

**Method**: Recombinant Pla l 1.0101 was expressed as a non-glycosylated and non-tagged protein in *E. coli* Rosetta-gami B pLysS. The protein was purified using cation exchange and size exclusion chromatographies. Physico-chemical properties of the purified recombinant protein were analyzed using gel electrophoresis, circular dichroism (CD), dynamic light scattering (DLS) and Fourier transform infrared spectroscopy (FTIR). The 3-dimensional (3-D) structure was solved by X-ray crystallography. IgE-binding activity of Pla l 1 was investigated in ELISA and IgE cross-inhibition with Ole e 1, Fra e 1, Phl p 11, Che a 1, and Sal k 5 using sera from Austrian plantain pollen allergic patients.

**Results**: Pla l 1 was purified to a purity of >95 % and a yield of 24 mg per liter of culture. DLS revealed a hydrodynamic radius R_h_ of 2.04 nm, indicating that Pla l 1 is monomeric. CD spectrometry showed an unusual spectrum with two negative minima and upon thermal denaturation a T_m_ of 68 °C was determined. FTIR analysis predicted a beta strand content of 40 %. X-ray crystallography revealed a barrel-like structure shaped by six antiparallel beta strands which is stabilized by three disulfide bonds. Structural modelling of all Ole e 1-like homologues was consistent with a common fold, whereby highest structural similarity was observed with a cell wall surface anchor protein. Besides sensitization to Pla l 1, Austrian patients’ sera were also IgE-reactive to Fra e 1/Ole e 1 and Phl p 11. Notably, none of the homologs was able to substantially inhibit IgE binding to solid-phase Pla l 1.

**Conclusions**: Within this study we were able to solve the 3-D structure of Pla l 1 unravelling for the first time the Ole e 1-like fold. Although Ole e 1-like proteins share similar tertiary structures, IgE cross-reactivity among non-phylogenetically related members was limited and consequently Pla l 1 should be regarded as marker allergen for primary *Plantago* sensitization.

Supported by the Austrian BMWFW, the NF RTD, and by a Start-up Grant of the Province of Salzburg.

### Symposium 2: New allergen molecules in the spotlight

#### O3 Identification of the cysteine protease Amb a 11 as a novel major allergen from short ragweed (*Ambrosia artemisiifolia*)

##### Philippe Moingeon, Rachel Groeme, Julien Bouley, Véronique Bordas, Maxime Le Mignon, Laetitia Bussières, Aurélie Lautrette, Laurent Mascarell, Vincent Lombardi, Véronique Baron-Bodo, Henri Chabre, Thierry Batard, Emmanuel Nony

###### STALLERGENES, Antony, France

**Correspondence**: Philippe Moingeon

*Clinical and Translational Allergy* 2016, **6(Suppl 2)**:O3

**Background**: Allergy to pollen from short ragweed (*Ambrosia artemisiifolia*) is a severe and expanding health problem in Europe and Northern America. Amb a 1 has been so far the only major allergen characterized in ragweed pollen. Herein, we report on the identification of Amb a 11 as a new major allergen, likely important for diagnosis and therapeutic purposes.

**Materials and methods**: Ragweed pollen proteins were submitted to high-resolution 2D gel electrophoresis and tested for IgE-reactivity using sera from 92 American or European ragweed-allergic donors. Pollen transcriptome sequencing, mass spectrometry (MS) and recombinant DNA technologies were applied to characterize new IgE-binding proteins.

**Results**: High-resolution IgE immunoblotting experiments revealed that 50 out of 92 ragweed-allergic patients were sensitized to a 37 kDa-allergen distinct from Amb a 1. The full-length cDNA sequence for this molecule was obtained by PCR cloning, following MS sequencing combined with ragweed pollen RNA-Seq. This new allergen, termed Amb a 11 is a 262-amino acid thiol protease of the papain family, expressed as a combination of isoforms and glycoforms, following proteolytic removal of N- and C-terminal pro-peptides from a pro-form. X-ray crystallography confirmed a high structural homology with known cysteine proteases, such as the mite Der p 1 allergen. The protease activity of Amb a 11 as well as its capacity to activate basophils from ragweed allergic patients were documented. The recombinant pro-Amb a 11 zymogen was refolded *in vitro* from *E. coli* inclusion bodies and matured at pH 5. This non glycosylated recombinant molecule was recognized by serum IgEs from 60 % of ragweed pollen-allergic patients.

**Conclusions**: We identified the cysteine protease Amb a 11 as a novel major allergen from ragweed pollen. Based on IgE reactivity documented in a majority of American and European patients, and given its cysteine protease activity, Amb a 11 should be considered as an essential component for diagnosis and specific immunotherapy of common ragweed allergy.

**Keywords**: Ragweed-pollen; Cysteine protease; New allergen

#### O4 Production and characterization of polybia paulista recombinant antigen 5: a valuable diagnostic tool

##### Karine Marafigo De Amicis^1^, Alexandra Sayuri Watanabe^1^, Daniele Danella Figo^1^, José Roberto Aparecido Dos Santos-Pinto^2^, Mario Sergio Palma^2^, Fabio Fernandes Morato Castro^1^, Jorge Kalil^1^, Therese Wohlschlager^3^, Peter Briza^3^, Sabrina Wildner^3^, Fatima Ferreira-Briza^3^, Gabriele Gadermaier^3^, Keity Souza Santos^1^

###### ^1^University of Sao Paulo, Sao Paulo, Brazil; ^2^University of Sao Paulo State (UNESP), Rio Claro, Brazil; ^3^University of Salzburg, Salzburg, Austria

**Correspondence**: Karine Marafigo De Amicis

*Clinical and Translational Allergy* 2016, **6(Suppl 2)**:O4

**Background**: *Polybia paulista* is an aggressive social wasp found in Brazil. Many cases of allergy to *Polybia paulista* venom are reported but there is neither a diagnostic nor a treatment tool commercially available. Antigen 5 is shown to be a major allergen in other hymenoptera species playing an important role in cross-reactivity. Recombinant protein production of allergens allows interference-free diagnosis and treatment of patients as well as large scale production for commercial purposes.

**Materials and methods**: Mass spectrometry (MS) of natural venom was used for identification of antigen 5 from *Polybia paulista*. A gene optimized for *E. coli* expression was designed based on the amino acid sequence and a recombinant protein was produced in strain Rosetta-gami™ 2. Recombinant antigen 5 (rAg5) was purified by cation exchange chromatography. The primary sequence identity was analyzed by MS. Circular dichroism analysis was used to determine secondary structure composition and thermal denaturation. Molecular modeling was based on the crystal structure of the antigen 5 from *Vespula vulgaris* (PDB: 1QNX) using Expasy Swiss model tool. To test the stability, the protein was exposed to different temperatures ranging from 25 to −80 °C up to 96 h. IgE reactivity was assessed using sera from twenty-two Brazilian patients allergic to wasp venom.

**Results**: A novel isoform of antigen 5 from *Polybia paulista* was identified by MS. A recombinant non-tagged folded Ag5 was expressed in *E. coli* in the soluble fraction. The intact mass was 23,192.41, with a delta mass of −8 corresponding to the disulphide bonds formation. The rAg5 CD spectrum is similar to antigen 5 homologs in other hymenoptera species being a mixture of alpha helix and beta sheets. The protein sequence of rAg5 is 59.11 % identical to Ves v 5 but at structure level the identity is 60.89 %. The rAg5 is very stable, not being degraded when exposed a 25 °C temperature. The allergen was recognized by IgE from 11 of 22 *Polybia paulista* venom allergic patients (50 %).

**Conclusions**: This is the first time that an *E. coli* expression system used for recombinant antigen 5 production resulted in properly folded and stable allergen. Recombinant *Polybia paulista* antigen 5 is a valuable diagnostic tool this allergy.

**Keywords**: *Polybia paulista*; Antigen 5; Hymenoptera venom; Venom allergy

### Symposium 3: Progress in molecular and cellular diagnosis

#### O5 Basophil activation test with recombinant Pru p 3; identifying genuine peach allergic patients

##### Margaretha Faber^1^, Athina Van Gasse^1^, Vito Sabato^1^, Margo M. Hagendorens^1^, Chris H. Bridts^1^, Luc S. De Clerck^1^, Araceli Diaz Perales^2^, Didier Ebo^1^

###### ^1^University of Antwerp, Antwerp, Belgium; ^2^Plant Biotechnology Institute, Madrid, Spain

**Correspondence**: Margaretha Faber

*Clinical and Translational Allergy* 2016, **6(Suppl 2)**:O5

**Background**: Non-specific lipid transfer proteins (ns-LTPs) are an important cause of plant food allergy and symptoms range from mild to anaphylactic reactions. Pru p 3 (*Prunus persica*) is frequently described as the most recognized ns-LTP allergen. Diagnosis of ns-LTP related allergy is difficult, as patients frequently show an asymptomatic ns-LTP sensitization.

**Materials and methods**: We included 2 peach allergic patients and 7 peach tolerant patients, all of these patients showed sIgE reactivity to rPru p 3 (FEIA ImmunoCAP, Thermofisher Scientific, Sweden). Besides that a group of pollen allergic patients (n = 9) without Pru p 3 sensitization and a healthy control group (n = 7) was included. Sera of all individuals were tested for sIgE reactivity to rPru p 3, rBet v 1, rArt v 1, rPhl p 1 and rPhl p 5b and a basophil activation test (BAT) with 4 concentrations of rPru p 3 was performed.

**Results**: Definite peach allergic patients showed a sIgE to rPru p 3 of 6.11 and 0.44 kUa/L. The peach tolerant group with sIgE reactivity to the ns-LTP of peach demonstrated a median of 1.2 kUa/L (0.13–4.75). No sIgE antibodies to rPru p 3 were detected in the pollen allergic patients and the healthy control individuals. BAT with rPru p 3 was most discriminative at a concentration of 1 µg/ml. For this concentration, both peach allergic patients showed a clear up-regulation of CD63. None of the Pru p 3 sensitized but peach tolerant individuals showed activation of their basophils. Neither the atopic patients without Pru p 3 sensitization and control individuals showed an up-regulation of CD63 on their basophils.

**Conclusions**: BAT with rPru p 3 might be a sensitive and specific test to document Pru p 3 induced peach allergy. Patient groups should be enlarged in order to further validate the basophil activation test with rPru p 3.

**Keywords**: Basophil activation test; Diagnosis; LTP; Peach

#### O6 Nanofluidic technology enables rapid, near-patient quantification of allergen-specific IgE

##### Petra Zavadakova, Aurélie Buchwalder, Fabien Rebeaud, Iwan Märki

###### ^1^Abionic SA, Lausanne, Switzerland

**Correspondence**: Fabien Rebeaud

*Clinical and Translational Allergy* 2016, **6(Suppl 2)**:O6

**Background**: Quantification of allergen-specific IgE is challenging due to the low analytical sensitivity required and the need for accurate and reliable results. Today, this is mainly performed in diagnostic laboratories, and therefore the delivery of results to allergists and patients takes often days. We have developed a nanofluidic-based biosensor containing a nanochannel that accelerates molecular interactions and thereby drastically reduces incubation time from hours to a few minutes. Several biosensors are assembled into one capsule which allows a multiple allergen components-based in vitro diagnosis to be performed within one test. Capsules are then analyzed in the abioSCOPE, a miniaturized automated fluorescence microscope. Complexes of fluorescently labelled antibodies and allergen-specific IgE from a patient sample are specifically captured on the sensing area of the biosensors and, upon excitation, emit fluorescence that is proportional to the analyte concentration.

**Materials and methods**: Capsules containing allergen-specific IgE tests were analyzed in the abioSCOPE to determine the analytical specificity of the tests and the potential effect of high total circulating IgE content on test results. Allergen-specific IgE levels from poly-sensitized serum samples have been compared to concentrations measured in Phadia 250.

**Results**: High analytical specificity is indeed achieved as illustrated in a competitive inhibition study with Bet v 1 and Phl p 5a. Moreover, the test results are not influenced by high levels of total circulating IgE. Serum samples from poly-sensitized patients were analyzed in capsules containing five key inhaled allergens of Central Europe (Bet v 1, Phl p 5a, Der p 1, Fel d 1, Can f 1). A good agreement was observed between allergen-specific IgE values measured in Phadia 250 and in the abioSCOPE.

**Conclusions**: Together, these results highlight the great potential of nanofluidic technology as a rapid, robust and easy-to-use method for the evaluation of patients suspected of having an allergic disease. Combining a component-based approach with patient history and in vivo skin tests allows allergists to provide their patients with a comprehensive diagnosis that supports the best medical decision within a single consultation.

**Keywords**: Components based diagnosis; Specific IgE; Quantification; Near-patient diagnostics; Nanofluidics

### Symposium 4: Relevance of molecular diagnostics for intervention and treatment

#### O7 Longitudinal analysis of Bet v 1-specific epitope repertoires during birch pollen immunotherapy

##### Barbara Gepp^1^, Nina Lengger^1^, Christian Möbs^2^, Wolfgang Pfützner^2^, Christian Radauer^1^, Barbara Bohle^3^

###### ^1^Department of Pathophysiology and Allergy Research, Medical University of Vienna, Vienna, Austria; ^2^Department of Dermatology and Allergology, Philipps University Marburg, Marburg, Germany; ^3^Department of Pathophysiology and Allergy Research & Christian Doppler Laboratory for Immunomodulation, Medical University of Vienna, Vienna, Austria

**Correspondence**: Barbara Gepp

*Clinical and Translational Allergy* 2016, **6(Suppl 2)**:O7

**Background**: Allergen-specific immunotherapy (AIT) is the only curative therapy for allergy. However, little is known about the epitope diversity of antibody repertoires during AIT. Therefore, we aimed to monitor the development of the Bet v 1-specific IgE, IgG1 and IgG4 repertoires in narrow time intervals during 3 years of subcutaneous birch pollen immunotherapy.

**Materials and methods**: In order to study antibody binding to conformational epitopes of the major birch pollen allergen, Bet v 1, four non-overlapping contiguous Bet v 1-specific surface areas were grafted onto Api g 1, the Bet v 1-homologue from celeriac. Sera from 11 birch pollen-allergic patients with improved symptom and medication scores were collected before (time point 0) and after 1, 3, 6, 12, 18, 24, 30 and 36 months of AIT. Antibody binding to Bet v 1, the four Bet v 1-specific areas on the chimeras and Api g 1 (scaffold protein) was assessed by ELISA.

**Results**: In the majority of the patients, Bet v 1-specific IgE-levels increased during the early phase of treatment followed by a gradual decrease. 7/11 patients (64 %) displayed slightly lower IgE-levels after 36 months of AIT compared to before therapy. Bet v 1-specific IgG4 was induced in all patients, whereas Bet v 1-specific IgG1 was induced in 7/11 patients. The IgG1 response increased earlier than IgG4 (*P* = 0.016). The patterns of IgE-recognition of the four Bet v 1-specific areas on the chimeras did not change over time in most patients. Similarly, once induced, the patterns of IgG1- and IgG4-binding to the chimeras remained unchanged during AIT in 4/7 and 8/11 patients, respectively. Furthermore, epitope profiles of IgE, IgG1 and IgG4 differed among each other in the patients. IgE-binding to all four chimeras was observed in 7/11 (64 %) patients. In contrast, only two and three patients showed IgG1- or IgG4-binding to all chimeras, respectively.

**Conclusions**: Taken together, the repertoire of Bet v 1-specific IgE and induced IgG1 and IgG4 remained unchanged during AIT with IgE showing a higher diversity than IgG1 or IgG4. Therefore, not all IgE-binding epitopes can be blocked by IgG4 or IgG1 due to direct epitope competition.

This study was supported by the Austrian Science Fund (FWF) grants SFB F4608 and F4610 and by the Christian Doppler Laboratory for Immunomodulation.

**Keywords**: Bet V 1; Immunotherapy; Epitope recognition profiles

#### O8 A natural CCD-free tool: is polistes sp. venom suitable for polybia paulista diagnosis and therapy?

##### Karine Marafigo De Amicis^1^, Alexandra Sayuri Watanabe^2^, Clovis Eduardo Galvao^3^, Daniele Danella Figo^4^, Jose Roberto Aparecido Santos-Pinto^5^, Mario Sergio Palma^5^, Fabio Fernandes Morato Castro^6^, Jorge Kalil^7^, Fatima Ferreira^8^, Gabriele Gadermaier^9^, Keity Souza Santos^10^

###### ^1^Laboratory of Clinical Immunology and Allergy-LIM60, Division of Clinical Immunology and Allergy, Department of Medicine, School of Medicine, University of Sao Paulo,, Sao Paulo, Brazil; ^2^Division of Allergy, Clinics Hospital of School of Medicine, University of Sao Paulo,, Brazil; ^3^Division of Allergy, Clinics Hospital of School of Medicine, University of Sao Paulo,, Sao Paulo, Brazil; ^4^Laboratory of Clinical Immunology and Allergy-LIM60, Division of Clinical Immunology and Allergy, Department of Medicine, School of Medicine, University of Sao Paulo, Sao Paulo, Brazil; ^5^Institute of Biosciences of Rio Claro, University of Sao Paulo State (UNESP), Rio Claro, Brazil; ^6^Division of Allergy, Clinics Hospital of School of Medicine, University of Sao Paulo, Sao Paulo, Brazil; ^7^Laboratory of Immunology, Heart Institute (InCor), LIM19, University of Sao Paulo, School of Medicine, Brazil; ^8^Department of Molecular Biology, Division of Allergy and Immunology, University of Salzburg, Sao Paulo, Austria; ^9^Department of Molecular Biology, Division of Allergy and Immunology, University of Salzburg, Brazil; ^10^Laboratory of Clinical Immunology and Allergy-LIM60, Division of Clinical Immunology and Allergy, Department of Medicine, School of Medicine, University of Sao Paulo, Rio Claro, Brazil

**Correspondence**: Keity Souza Santos

*Clinical and Translational Allergy* 2016, **6(Suppl 2)**:O8

**Background**: *Polybia paulista* is an aggressive social wasp, causing many accidents. Besides the importance of *P. paulista* allergy in Brazil, there is neither a diagnostic nor a treatment tool commercially available. Hence *Polistes* sp. venom extract is used for skin tests and immunotherapy of Brazilian wasp venom allergic patients. The proximity of physicians and researchers in our group allows the use of ELISA and western blot analysis as a diagnosis support tool. *Polistes* venom is devoided of cross-reactive carbohydrate determinants (CCDs) allowing CCD-free differential diagnosis of *P. paulista* allergy. The question regarding protection of *Polybia* allergic patients treated with *Polistes* venom extract in the immunotherapy remains unanswered.

**Materials and methods**: Twenty patients with clinical history of anaphylaxis to wasp venom were included and tested by ImmunoCAP^®^ to *Polistes*(i4) and MUXF3 (o214). Skin prick (SPT) and intradermal test (ID) was performed using commercial *Polistes* venom extract. ELISA and western blot (WB) was made using commercial *Polistes* venom extract and *Polybia* venom extract produced by our group besides HRP ELISA for IgE anti-CCD detection.

**Results**: Patients presented clinical manifestations of anaphylaxis with symptoms that included urticaria, angioedema, diarrhea, respiratory symptoms and loss of consciousness. SPT was negative and the allergy was confirmed by ID test. In the IgE WB, all patients were positive to *Polybia* and *Polistes* recognizing multiple IgE-reactive bands different in each venom extract. In spite of this in IgE ELISA where the magnitude of IgE response to *Polybia* is 7 times higher than to *Polistes* only 5 patients are positive for both venoms from those only 2 are positive for CCD. Results from ELISA, WB and CAP for *Polistes* are not coincident. Patients positive in CAP for *Polistes* are many times negative in ELISA.

**Conclusions**: *Polistes* venom extract is not suitable for diagnosis and treatment of *Polybia* patients as the sensitization pattern showed some differences. Despite the already reported cross-reactivity with homologs, possible new allergens unique in each venom were observed presenting distinct molecular masses not yet described. Conflicting results using extract goes along with previously reported problems in standardization of extracts used for diagnosis and treatment. Recombinant production and characterization of the *Polybia* venom allergens would help to elucidate the diagnoses and allow proper therapy for *P. paulista* venom allergic patients.

### Symposium 5: The advent of molecular allergology in epidemiology

#### O9 Peanut oleosins: from identification to diagnostic testing

##### Christian Schwager^1^, Skadi Kull^1^, Frauke Schocker^1^, Jochen Behrends^2^, Wolf-Meinhard Becker^1^, Uta Jappe^3^

###### ^1^Division of Clinical and Molecular Allergology, Research Center Borstel, Airway Research Center North (ARCN), Member of the German Center for Lung Research (DZL), Borstel, Germany; ^2^Core Facility Fluorescence Cytometry, Research Center Borstel, Borstel, Germany; ^3^Division of Clinical and Molecular Allergology, Research Center Borstel, Airway Research Center North (ARCN), Member of the German Center for Lung Research (DZL), Borstel, Germany; Interdisciplinary Allergy Outpatient Clinic, Department of Pneumology, University of Luebeck, Luebeck, Germany

**Correspondence**: Uta Jappe

*Clinical and Translational Allergy* 2016, **6(Suppl 2)**:O9

**Background**: A rising prevalence of peanut allergy has been observed within the past decades. Routine diagnostic measures applying aqueous extracts (e.g. for skin prick test or IgE-detection measures) can lead to false negative results due to a lack of potential lipophilic allergens. Oleosins, a class of oil body proteins, have been found to be triggers of severe allergic reactions to hazelnut and sesame. Since data for peanut oleosins are scarce, the aims of the study were the isolation, the molecular characterization and the assessment of the oleosin allergenicity. Moreover, we searched for a specific diagnostic approach to close the existing detection gap in the established peanut allergy diagnostics.

**Materials and methods**: A comprehensive oleosin isolation procedure was established which comprises extraction and subsequent step by step purification of oil bodies along with preparative electrophoresis. Protein identification was achieved by N-terminal sequencing, peptide mass fingerprinting and homology search against databases. The IgE-binding capacity of oleosins was evaluated in western blot experiments with sera of peanut-allergic, tolerant and non-allergic individuals. A flow cytometric basophil activation test was used for the diagnosis of oleosin-allergic patients.

**Results**: Oleosins were isolated and purified from the complex lipophilic matrix of peanut. Mass spectrometry analysis identified all known eight peanut oleosins, ranging from 15.5 to 17.5 kDa as well as further oil body related proteins (steroleosins and caleosins). IgE-binding to purified oleosins was observed in 20 of 39 sera from peanut-allergic patients by means of immunoblotting. Thus, oleosins meet the criteria to be classified as new allergens and isoallergens according to the WHO/IUIS allergen nomenclature subcommittee, and were accepted and designated as Ara h 14 and Ara h 15 in May 2015. Positive immunoblot results were observed solely in patients suffering from severe allergic reactions. IgE-dependent basophil activation was induced *in vitro* in a dose-dependent manner in peanut-allergic patients, but not in controls.

**Conclusions**: A novel strategy for the simultaneous isolation of the lipophilic peanut allergens oleosins was successfully established. The allergenicity of the distinct oleosins was illustrated by IgE-detection in immunoblot and confirmed by a functional cellular assay, the basophil activation test. Oleosins proved to be relevant allergens that may cause more severe allergic reactions.

**Keywords**: Oleosins; Allergens; Isolation; Diagnosis

#### O10 Endotypes of oral allergy syndrome in childhood: a molecular diagnostic approach

##### Carla Mastrorilli^1^, Salvatore Tripodi^2^, Carlo Caffarelli^1^, Riccardo Asero^3^, Arianna Dondi^4^, Giampaolo Ricci^5^, Carlotta Povesi Dascola^1^, Elisabetta Calamelli^5^, Andrea Di Rienzo Businco^2^, Annamaria Bianchi^6^, Tullio Frediani^7^, Carmen Verga^8^, Iride Dello Iacono^9^, Diego Peroni^10^, Giuseppe Pingitore^11^, Roberto Bernardini^12^, Paolo Maria Matricardi^13^

###### ^1^Pediatric Dept, Unit of Allergy and Immunology in Evolutive Age, Department of Clinical and Experimental Medicine, University of Parma, Parma, Italy; ^2^Pediatric Dept and Pediatric Allergology Unit, Sandro Pertini Hospital, Rome, Italy; ^3^Allergology Service, San Carlo Clinic, Paderno Dugnano, Italy; ^4^Pediatric Unit, Dept. for Mother and Child, Ramazzini Hospital, Carpi, Italy; ^5^Pediatric Unit, Department of Medical and Surgical Sciences, University of Bologna, Bologna, Italy; ^6^Operative Complex Unit of Pediatrics and Neonatal Patology, Mazzoni Hospital, Ascoli Piceno, Italy; ^7^Pediatric Department, La Sapienza University, Rome, Italy; ^8^Azienda Sanitaria Locale Salerno, Salerno, Italy; ^9^Pediatric Unit, Fatebenefratelli Hospital, Benevento, Italy; ^10^Pediatric section, Dept of Life and Reproduction Sciences, University of Verona, Verona, Italy; ^11^Pediatric Unit, Grassi Hospital, Rome, Italy; ^12^Pediatric Unit, San Giuseppe Hospital, Empoli, Italy; ^13^Department of Pediatric Pneumology and Immunology, Charité Medical University, Berlin, Germany

**Correspondence**: Carla Mastrorilli

*Clinical and Translational Allergy* 2016, **6(Suppl 2)**:O10

**Background**: Oral allergy syndrome (OAS) is a common adverse reaction to the ingestion of plant foods in patients with pollen-related allergic rhinoconjunctivitis. There is limited epidemiological information on childhood OAS in regions with highly complex pollen exposure. We aimed the present study to investigate OAS, its risk factors and underlying IgE sensitization patterns in children living in a Mediterranean country.

**Materials and methods**: This cross-sectional study assessed 1271 children (age 4–17 years) with pollen-related seasonal allergic rhinoconjunctivitis (SAR), enrolled by 16 Italian outpatient clinics. Data about OAS symptoms and their food triggers were acquired by a standardized questionnaire. Skin prick tests (SPTs) were performed with commercial pollen and food extracts. Specific IgE to the panallergens Phl p 12 (profilin), Bet v 1 (pathogenesis related protein class 10, PR-10) and Pru p 3 (non-specific lipid transfer protein, nsLTP) were tested by ImmunoCAP FEIA.

**Results**: Oral allergy syndrome was observed in 300/1271 (23.6 %) children with a prevalence increasing from age 5 year (24 %) to age 16 year (37 %). Risk factors for OAS were a female gender (p < 0.001), maternal OAS (p < 0.001), living in the Northern Italy (p < 0.05), and passive exposure to tobacco smoke (p < 0.01). OAS was strongly associated with other SAR comorbidities: asthma (p < 0.001), anaphylaxis (p < 0.005), urticaria and/or angioedema (p < 0.001), atopic dermatitis (p < 0.001), and gastrointestinal symptoms (p < 0.001). IgE to one or more panallergens were found in 229/300 (76.3 %) of OAS affected children. Cucurbitaceae (melon and watermelon) were associated with IgE sensitization to Phl p 12; Rosaceae (apple, peach, pear) were strongly associated with IgE sensitization to Bet v 1; peanuts and tree nuts (hazelnut and walnut) were associated with IgE sensitization to Pru p 3. Kiwi’s allergy was the most frequent cause of OAS in children not sensitized to any of the three examined panallergens.

**Conclusions**: In a geographic area with complex pollen exposure, OAS is a highly frequent complication of childhood seasonal allergic rhinitis, even at preschool age. IgE sensitization to profilin, PR-10 and nsLTP explains most but not all of OAS childhood morbidity and kiwi allergy is the most important trigger. These results call for the inclusion of early diagnosis and OAS treatment in guidelines of pollen-related seasonal allergic rhinitis in childhood.

**Keywords**: Oral allergy syndrome; Panallergens; Profilin; Lipid-transfer protein; PR-10

### Symposium 6: Molecular AIT: which approaches will make it to market?

#### O11 Mbc4: an innovative molecule to tackle birch pollen and concomitant food allergies

##### Heidi Hofer^1^, Claudia Asam^1^, Michael Hauser^1^, Peter Briza^1^, Martin Himly^1^, Christof Ebner^2^, Fatima Ferreira^1^

###### ^1^University of Salzburg, Salzburg, Austria; ^2^Allergieambulatorium Reumannplatz, Vienna, Austria

**Correspondence**: Heidi Hofer

*Clinical and Translational Allergy* 2016, **6(Suppl 2)**:O11

**Background**: Amongst tree pollen allergies, birch is one of the main causes of winter and spring pollinosis in the temperate climate zone of the Northern hemisphere. This is caused by sensitization of patients towards Bet v 1, the major birch pollen allergen. Most of these patients not only suffer from rhinitis and asthma, but also develop adverse reactions towards various fruits, nuts, and vegetables—most frequently against apple and hazelnut. These reactions are limited to the oral cavity and triggered by food proteins structurally related to Bet v 1, as these allergens are also able to cross-link Bet v 1 specific IgE on mast cells and basophils. Therefore, we designed a hybrid molecule (MBC4) by assembling parts of Bet v 1 from birch, Mal d 1 from apple and Cor a 1 from hazelnut. For an increased safety profile, we introduced a mutation into the backbone of MBC4 to reduce its IgE binding capacity.

**Materials and methods**: Parental allergens, as well as MBC4 were expressed in *E. coli*, purified to homogeneity and characterized physico-chemically. Sera from clinically diagnosed patients with birch pollen allergy and concomitant pollen-food syndrome towards apple and hazelnut were analyzed by IgE ELSIA and mediator release assay to determine the IgE binding capacity of MBC4. Additionally, we monitored the immunological behavior *in vivo* in a mouse model.

**Results**: Significantly reduced IgE binding and allergenic effector function of MBC4 was observed by ELISA and mediator release assay, respectively. Analyses of patients´ sera revealed that the hybrid molecule showed a hypoallergenic factor of 10000 when compared to Bet v 1. Although IgE binding was reduced, MBC4 was able to induce cross-reactive IgG antibodies to parental allergens *in vivo*, whereas murine IgE antibodies revealed a very limited cross-reactivity. Additionally, when splenocytes from mice where re-stimulated with either parental allergen or MBC4, a cross-reactive T cell response was observed in ELISpot assays.

**Conclusions**: As MBC4 revealed reduced IgE binding and allergic function in mice and man, and further was able to induce a cross-reactive T cell response as well as cross-reactive IgG antibodies, it presents itself as a suitable vaccine candidate to treat birch pollen and associated food allergies towards apple and hazelnut.

This work was supported by FWF L688 and ÖNB 12533 grants.

**Keywords**: Birch pollen allergy; Oral allergy syndrome; Allergen immunotherapy; Hybrid molecule

#### O12 Challenges and solutions associated with the production of recombinant Bet v 1 allergen as a therapeutic protein

##### Emmanuel Nony^1^, Maxime Le Mignon^1^, Pierrick Lemoine^1^, Karine Jain^1^, Kathy Abiteboul^1^, Monica Arvidsson^2^, Sabina Rak^2^, Philippe Moingeon^1^

###### ^1^Stallergenes SAS, Antony, France; ^2^Sahlgrenska University Hospital, Goteborg, Sweden

**Correspondence**: Emmanuel Nony

*Clinical and Translational Allergy* 2016, **6(Suppl 2)**:O12

**Background**: Up to 90 % of birch pollen allergic patients exhibit serum IgEs against Bet v 1, among which a majority reacts solely to this relevant allergen. Consequently, we produced a pharmaceutical grade recombinant Bet v 1.0101 molecule (rBet v 1) that was tested in patients via the sublingual route. Herein, we present some of the challenges faced during both the upstream and downstream pharmaceutical and clinical developments of this molecule.

**Materials and methods**: Recombinant Bet v 1 was expressed in *Escherichia coli* at the 1500 L scale and further purified by multiple chromatographic steps. Characterization methods including electrophoresis, liquid chromatography (LC), mass spectrometry (MS) or X-ray crystallography were decisive in order to identify and circumvent several issues related to the quality of the expressed or purified molecule. Safety and efficacy of rBet v 1 were assessed in a double-blind, placebo-controlled phase II study conducted in 483 european patients with allergic rhinoconjunctivitis to birch pollen.

**Results**: The LC-MS characterization confirmed the expected amino acid sequence with low levels of both deamidation and oxidation. Evidence for partially matured product was also documented. Moreover, in depth MS analysis revealed the presence of various amino acid substitutions. Both of those issues were solved by successive modifications of cell growth conditions. Despite multiple chromatographic steps and the absence of detection of host cell proteins (HCPs) by ELISA, MS characterization evidenced various HCPs that were removed through additional modifications of the downstream process. The later was further optimized since analyses showed the co-purification of a small molecule entrapped within Bet v 1 hydrophobic cavity, as confirmed by X-ray crystallography. Bet v 1 storage was modified in order to prevent its oxidation. Recombinant Bet v 1 tablets were evaluated in the first clinical study performed via the sublingual route with a recombinant molecule. Results confirmed that it is well tolerated by patients, with a level of efficacy consistent with the one observed with a birch pollen extract.

**Conclusions**: Even though Bet v 1 is a fairly simple molecule, its production as a recombinant biopharmaceutical product required extensive and iterative structural characterization as well as process optimizations in order to obtain GMP grade rBet v 1 for patients with birch pollen allergic rhinoconjunctivitis.

**Keywords**: Bet V 1; Birch pollen; Recombinant allergen; Sublingual immunotherapy

### Clinical Cases: Breakthroughs and headaches from CRD: interactive session

#### CC1 Anaphylaxis caused by lipid transfer proteins: a complex clinical pattern syndrome

##### Inês Mota, Filipe Benito Garcia, Angela Gaspar, Cristina Arêde, Susana Piedade, Graça Sampaio, Graça Pires, Luís Miguel Borrego, Cristina Santa-Marta, Mário Morais-Almeida

###### Immunoallergy Department, CUF Descobertas Hospital, Lisbon, Portugal

**Correspondence**: Inês Mota

*Clinical and Translational Allergy* 2016, **6(Suppl 2)**:CC1

**Background**: Lipid transfer proteins (LTP) are panallergens resistant to heat and pepsin digestion, found in many plant-foods, as fruits, vegetables, grains, peanut, tree nuts and also in pollens. It is a common cause of food-induced anaphylaxis (FIA) in adults living in Mediterranean area. LTP has also been proposed as a main cause of food-dependent exercise-induced anaphylaxis (FDEIA). We aimed to describe clinical characteristics and allergen sensitization profiles in patients with FIA related to LTP.

**Materials and methods**: Twenty eight patients were included [mean age 27.1 (SD ± 12.8) years, 29 % <18 years and 50 % male) with clinical history of FIA, whose allergological work-up confirmed sensitization to LTP. Patients were tested with a multiple plant-food and pollen panel and specific IgE to LTP allergens. LTP sensitization was assessed by *in vivo* (Pru p 3, LTP extract, Bial-Aristegui^®^) or by *in vitro* tests (specific IgE, ImmunoCAP / ISAC, ThermoFisher^®^).

**Results**: Median age of first anaphylactic episode was 26.5 [2-51] yrs; 46 % had asthma; 68 % were atopic and 54 % had pollinosis (mugwort, wall pellitory, plane tree, olive and cypress). Co-sensitization to profilins was found in 14 %. Overall in our center, LTP-induced anaphylaxis represents 15.7 % of all causes of FIA. Foods implicated in anaphylactic reactions were: *Rosaceae* fruits-36 % (peach and apple), tree nuts-29 % (walnut, cashew nut and hazelnut), seeds-25 % (sesame, sunflower seed and flaxseed) and other vegetables-21 % (peanut, green bean, goji berry, tomato and maize). In three cases the food implicated remained unidentified. Three patients had FDEIA (green bean, tree nuts, tomato and maize). Clinical manifestations were: mucocutaneous 100 %, respiratory 86 %, cardiovascular 25 % and gastrointestinal 21 %. In 75 % the reaction occurred within the first 30 minutes after food ingestion; 29 % had ≥3 FIA before etiologic diagnosis.

**Conclusions**: LTP are important allergens of FIA in Portugal. LTP sensitization is responsible for a heterogeneous group of offending plant-foods, taxonomically unrelated, which can induce recurrent reactions. It is a useful risk marker in patients with severe reactions due to an unknown cause. The association of LTP-induced anaphylaxis with pollinosis is relevant in our country. The unpredictable clinical expression depends on the effect of cofactors such as exercise. The management of avoidance plans can be particularly challenging due to LTP be a widely cross-reacting allergens in plant-foods.

**Keywords**: Lipid transfer proteins; Panallergens; Anaphylaxis; Food allergy; Food-dependent exercise-induced anaphylaxis

#### CC2 IgE sensitization profile in a patient with asteraceae pollen-exotic fruits association

##### Florin-Dan Popescu^1^, Mariana Vieru^1^, Florin-Adrian Secureanu^2^

###### ^1^Carol Davila University of Medicine and Pharmacy, Bucharest, Romania; ^2^Nicolae Malaxa Clinical Hospital, Bucharest, Romania

**Correspondence**: Florin-Dan Popescu

*Clinical and Translational Allergy* 2016, **6(Suppl 2)**:CC2

**Background**: There are few reports of allergic reactions to exotic fruits belonging to the *Sapindaceae* or *Anacardiaceae* plant families.

**Materials and methods**: We evaluated IgE sensitization profile in a 22-year-old woman with seasonal allergic rhinoconjunctivitis and history of food-dependent exercise-induced anaphylaxis to pistachio, anaphylaxis to lychee, mango and polyfloral bee polen dietary supplement. Skin prick testing (SPT) was performed with commercial extracts, prick-prick testing with edible foods, serum specific IgE levels were determined by immunoblot test systems and fluorescent enzyme immunoassay (FEIA). Oral challenge tests were not performed due to clear history and ethical reasons.

**Results**: SPT revealed positive wheal reactions to *Asteraceae* pollen extracts of *Ambrosia elatior* (10 mm), *Artemisia vulgaris* (4 mm) and *Helianthus annuus* (5 mm). SPT with liquid extract of lychee (5 % purée of *Litchi chinensis,* fruit of the *Sapindaceae* family) was also positive (4 mm). Prick-to-prick tests with both *Anacardiaceae* native fruits were positive: mango (*Mangifera indica*) fruit pulp (5 mm) and pistachio (*Pistacia vera*) drupe seed (3 mm). Values of serum pollen-specific IgE by immunoblot were high for ragweed (79 kU/L, EAST class 5) and mugwort (4.6 kU/L, EAST class 3) pollen. Specific IgE to mango (<0.35 kU/L, FEIA class 0) and lychee (<0.35 kU/L, EAST class 0) fruits were not detected. Molecular allergy assessment revealed involvement of profilin in this particular phenotype of pollen-food allergy association, specific IgE to rBet v2 (3.2 kU/L, EAST class 2) being considered a profilin sensitization biomarker, homologous (hm) with mugwort Art v 4, ragweed Amb a 8, lychee Lit c 4 and mango Man i 3 profilins. IgE sensitization to other tested allergen components was not found (<0.35 kU/L): rBet v 6 (isoflavone reductase hm with lychee Lit c IFR), rBet v 1 (protein hm with mango Man i 14 kD), rAra h 1 (7S vicilin hm with pistachio Pis v 3), rAra h 2 (2S albumin hm with pistachio Pis v 1), rAra h 3 (11S globulin hm with pistachio Pis v 2 and Pis v 5) and plant cross-reactive carbohydrate determinant (CCD).

**Conclusions**: In regions such as Southern Romania, where *Asteraceae* pollen sensitization is important, allergists must be aware of the potential role of cross-reactivity between mugwort and/or ragweed pollen and several exotic fruits, allergy risk of bee pollen supplements, and the role of *in vivo* and *in vitro* allergy testing, including component-resolved diagnosis.

**Consent**: Written informed consent was obtained from the patient for publication of this abstract and any accompanying images.

#### CC3 Food-dependent: exercise induced anaphylaxis. Which component to blame?

##### Rosa Anita Rodrigues Fernandes, Isabel Carrapatoso, Raquel Gomes, Celso Pereira, Ana Todo-Bom

###### CHUC, Coimbra, Portugal

**Correspondence**: Rosa Anita Rodrigues Fernandes

*Clinical and Translational Allergy* 2016, **6(Suppl 2)**:CC3

**Background**: We report the case of a 27-year-old man that started suffering from recurrent episodes of anaphylaxis, following the ingestion of meals containing mixed foods. The first crisis occurred when he was 20 years old, with generalized urticaria and angioedema, hypotension and blurred vision after ingestion of pizza.

Six months later another episode occurred after ingestion of meatballs and spaghetti. One year later, the same symptoms occurred with mixed food. Interestingly, all the systemic episodes developed 30-60 minutes after exercise. Self-administered adrenaline was prescribed and the patient was advised not to exercise at least 2 hours after eating. Between the ages of 21 and 26 no more anaphylactic episodes were reported. This patient lost follow up. Two years later he was readmitted in our outpatient department after treatment in the emergency room for exercise induced anaphylaxis. He also has complains of intermittent respiratory symptoms predominantly in the spring, since the age of 26.

**Materials and methods**: Skin prick tests (SPT) to commercial extracts of aeroallergens, foods allergens, Pru p 3 and profilin were carried out. Prick-to-prick tests (PP) and serum specific IgE determinations (sIgE) to airborne allergens and some plant foods were also performed, according to case history. Molecular diagnosis with ImunoCAP-ISAC and specific IgE determinations to some components were also performed. Oral provocation tests (OPT) were performed to some of the suspected foods.

**Results**: Sensitisation to grass pollens, mugwort and multiple foods was demonstrated as shown in the Table 1 below. OPT to peach and pizza crust with exercise was negative.

**Table 1:** Results of the investigation performed

	Prick (mm)	Prick-to-prick (mm)	sIgE (kU/L)	ImunoCap ISAC (103) (ISU)
Histamine	4			
*Phleum pratense*	4		5.91	
*Secale cereale*			3.13	
*Artemisia vulgaris*	6		17.2	
Peach	Negative	Pulp-5	20.9	
		Peel-6		
Tomato	4		14.8	
Wheat	Negative		4.05	
Gliadin	5			
Gluten	3		0.21	
Pru p 3	4		27.5	2.1
rTri a 14			19.9	
rTri a 19			0.04	
rPhl p 1			4.07	3.5

**Conclusions**: In this patient with recurrent food-dependent exercise anaphylaxis the culprit allergen seems to be a non-specificLTP, as sIgE to Pru p 3 and Tri a 14 were positive and sIgE to ω-5-gliadin negative. Non-specific LTPs have been associated with cofactor-enhanced food-dependent anaphylaxis in southern Europe. The variability of anaphylactic reactions occurred in this patient could be explained by the type of exercise, food with different wheat processing and the season of the year.

**Keywords**: Anaphylaxis; Tri A 14; Wheat; Exercise

**Consent**: Written informed consent was obtained from the patient for publication of this abstract and any accompanying images.

#### CC4 Anaphylaxis to intravenous iron preparations in a patient that tolerates oral administration

##### María Cecilia Martín Fernández De Basoa, Javier Barrios Regio, Juan De Castro Cordova, Antón Fernández Ferreiro

###### University Hospital Nuestra Señora de Candelaria, Santa Cruz De Tenerife, Spain

**Correspondence**: María Cecilia Martín Fernández De Basoa

*Clinical and Translational Allergy* 2016, **6(Suppl 2)**:CC4

**Background**: Oral administration of iron salts for iron deficiency treatment is usually well tolerated. Adverse gastrointestinal side effects are the most common. Exanthematous eruptions or anaphylactic reactions following a parenteral iron dextran injection or intravenous sucrose preparations are very uncommon

We report a case of anaphylactic reaction caused by intravenous administration of iron salts. We also present the results of the allergy study

**Materials and methods:**

**Case Description**

A 71-years-old woman with a personal history of type 2 diabetes, dyslipidemia and iron deficiency anemia was referred to our department to be tested for a possible allergic reaction to iron salts. The patient reported that after starting the infusion immediately presents generalized micropapular urticarial reaction associated with intense feeling of heat and hypotension, so the infusion was stopped and treated with intramuscular methylprednisolone and dexchlorpheniramine. However she tolerate oral iron in ferrous form

**Results**: Skin tests were performed with FERIV^®^ 20 mg/mL [sucrose and hydroxide iron (III)] and with excipients of the commercial formulations. Positive responses were obtained in intradermal tests with FERIV^®^ (1/1000 and 1/10,000). The excipients tested proved negative

Basophil activation test (BAT) were performed with FERIV^®^ and FERROCUR 40 mg [40 mg Fe^3+^] oral solution (tolerated by the patient). Positive responses were obtained in BAT with FERIV^®^ (concentrations: 10^−1^ and 10^−2^) and FERROCUR (concentration: 10^−2^). The excipients tested proved negative. BAT performed with FERIV^®^ and FERROCUR in 2 healthy controls were also negative

We could not perform an intradermal tests with FERROCUR because the patient had a multiple organ dysfunction syndrome and died

**Conclusions**: Iron salts are seldom responsible for allergic reactions. A few cases of eruptive dermatosis have been reported However, very few studies examine mild anaphylactic reactions or severe anaphylactic reactions after intravenous administration.

The pathogenesis of anaphylactic reactions to iron dextran has not yet been elucidated. In most reports, these episodes occur after the first contact with the drug. This should support an anaphylactoid origin rather than a truly anaphylactic reaction. In our case, both the positive skin tests and the negative results in the control subjects tested corroborate the specificity of the response and demonstrate the existence of an immediate, possibly IgE-mediated hypersensitivity mechanism

**Keywords**: Intravenous iron salts; Anaphylaxis; Basophil activation test

**Consent**: Written informed consent was obtained from the patient for publication of this abstract and any accompanying images.

#### CC5 IgE sensitization pattern in an adult patient with oral allergy syndrome to peanuts and pollinosis from southern Romania

##### Florin-Dan Popescu^1^, Mariana Vieru^1^, Florin-Adrian Secureanu^2^

###### ^1^Carol Davila University of Medicine and Pharmacy, Bucharest, Romania; ^2^Nicolae Malaxa Clinical Hospital, Bucharest, Romania

**Correspondence**: Florin-Dan Popescu

*Clinical and Translational Allergy* 2016, **6(Suppl 2)**:CC5

**Background**: Many IgE-mediated food allergies in adults are caused by cross-reactivity between aeroallergens and food allergens. Peanuts are not frequently involved in these pollen-food syndromes or associations.

**Materials and methods**: From adult patients with pollen-induced allergic rhinitis referred to our allergy clinic in one year-period, screened for suggestive history of allergy to *Arachis hypogaea* legume, we found only one 34-year-old female with moderate-severe persistent allergic rhinoconjunctivitis, positive skin prick tests to five grass mix (6 mm wheal) and *Artemisia vulgaris* (5 mm wheal) pollen extracts, and convincing history of oral allergy syndrome to peanuts. Specific IgE sensitization pattern was assessed using multi-parameter immunoblot test system for defined partial allergen diagnosis.

**Results**: Serum specific IgE levels were found increased for Thimothy grass pollen (99 kU/L) and mugwort pollen (2.5 kU/L). IgE sensitization to single purified allergen components revealed genuine sensitization to *Pooideae* grass pollen: Phl p 1 (55 kU/L) and Phl p 5 (17.5 kU/L). Serum IgE to cross-reactive carbohydrate determinant marker was absent (<0.35 kU/L), important *in vitro* assessment for excluding false positive IgE to peanuts in patients with grass pollen allergy. Specific IgE against plant cross-reactive allergen components, such as grass polcalcin (Phl p 7 cross-reactive with weed Art v 5) and profilin (Phl p 12 cross-reactive with weed Art v 4), were not found. Low antibody serum titer of specific IgE to whole peanut extract was detected (0.7 kU/L). Serum specific IgE against peanut allergen components involved in early sensitization and systemic reactions, the storage proteins rAra h 1 (7S vicilin), rAra h 2 (2S albumin) and rAra h 3 (glycinin legumin), and the nonspecific lipid transfer protein (LTP) involved in oral and/or systemic reactions rAra h 9, were not detected (<0.35 kU/L). IgE sensitization to defensins Art v 1, Ara h 12, Ara h 13 may explain cross-reactivity. In addition, specific IgE to Ara h 5 (profilin), Ara h 8 (PR-10 Bet v 1-like protein), Ara h 10 and Ara h 11 (oleosins) were not available for component resolved diagnosis, but IgE to profilin biomarker rBet v 2 and PR-10 biomarker rBet v 1 were not detected (<0.35 kU/L).

**Conclusions**: A multiplex immunoblot assessment of single purified allergen peanut components can be used to determine the risk of systemic reactions in selected patients with pollinosis and IgE sensitization to peanuts.

**Consent**: Written informed consent was obtained from the patient for publication of this abstract and any accompanying images.

#### CC6 Evidence of specific IgE to plant-derived cross-reactive carbohydrate determinant in a patient with delayed anaphylaxis to red meat

##### Mariana Vieru^1^, Florin-Dan Popescu^1^, Florin-Adrian Secureanu^2^

###### ^1^Carol Davila University of Medicine and Pharmacy, Bucharest, Romania; ^2^Clinical Hospital Nicolae Malaxa, Bucharest, Romania

**Correspondence**: Mariana Vieru

*Clinical and Translational Allergy* 2016, **6(Suppl 2)**:CC6

**Background**: It was recently reported that red meat allergic patients have selective IgE responses to galactose-alpha-1,3-galactose (alpha-gal) found in edible non-primate mammalian meat, and other common glycans reactive in allergic disease are not targets of such IgE.

**Materials and methods**: The serum of an adult male with recurrent episodes of delayed anaphylaxis and urticaria after ingestion of red meat (pork, beef and lamb), history of multiple tick bites and other arthropod exposure in a forest habitat from the Romanian Plain, with no history of rhinitis and/or asthma, assessed *in vitro* by ELISA for specific IgE to pork, beef and mutton meats, was evaluated for serum specific IgE against *Pooideae* subfamily grass pollen and *Betulaceae* family trees/shrubs pollen using an immunoblot system with the test kit containing strips coated with parallel lines of different pollen allergen extracts, and for serum specific IgE against single purified allergen components using a multiparameter immunoblot system for defined partial allergen diagnostics, both test kits assessing also IgE to cross-reactive carbohydrate determinant (CCD) marker of plant origin. Bromelain is a glycoprotein used for checking the cross-reactivity between a glycan and other glycoproteins since its MUXF3 carbohydrate chain is found in many plant proteins.

**Results**: This patient with delayed anaphylaxis to red meat and elevated serum specific IgE levels against beef, pork and sheep meat, had negative skin prick tests to *Betula* and *Pooideae* pollen extracts, but elevated serum levels of specific IgE to pollen of sweet vernal grass (45 kU/L, EAST class 4), orchard grass (35 kU/L, EAST class 4), Timothy grass (41 kU/L, EAST class 4), cultivated rye (41 kU/L, EAST class 4), and birch (11.5 kU/L, EAST class 3). High levels of serum IgE to plant-based CCD were evidenced (13.5 kU/L, EAST class 3), but serum IgE to recombinant non-glycosylated pollen specific allergen components: beta-expansin rPhl p 1, ribonuclease rPhl p 5, PR-10 protein rBet v 1, recombinant non-glycosylated pollen cross-reactive allergen components: polcalcins rBet v 4 and rPhl p 7, profilins rBet v 2 and rPhl p12, and isoflavone reductase rBet v 6, were not detected (<0.35 kU/L, EAST class 0).

**Conclusions**: This is an isolated case of adult-onset delayed food allergy to red meat, in a patient with significant arthropod exposure, associated with serum IgE to plant-derived CCD.

**Consent**: Written informed consent was obtained from the patient for publication of this abstract and any accompanying images.

## POSTER PRESENTATIONS

### Poster Session 1: Molecular allergology and epidemiology

#### P1 Atopic children produce stronger and more frequent IgG responses than non-atopic children: longitudinal data from the German MAS birth cohort

##### Olympia Tsilochristou^1^, Serena Perna^1^, Alina Schwarz^1^, Alexander Rohrbach^1^, Antonio Cappella^2^, Laura Hatzler^1^, Carl-Peter Bauer^3^, Ute Hoffmann^3^, Johannes Forster^4^, Fred Zepp^5^, Antje Schuster^6^, Raffael D’amelio^7^, Ulrich Wahn^1^, Thomas Keil^8^, Susanne Lau^1^, Paolo Maria Matricardi^1^

###### ^1^Department of Pediatric Pneumology & Immunology, Charité-Universitätsmedizin Berlin, Berlin, Germany; ^2^Department of Clinical and Molecular Medicine, Sapienza University of Rome, S. Andrea University Hospital, Rome, Italy; ^3^Department of Pediatrics, Technical University of Munich, Munich, Germany; ^4^Department of Pediatrics St Hedwig, St Josefs Hospital, Freiburg, Germany; ^5^Department of Pediatrics and Adolescent Medicine, University Medicine Mainz, Mainz, Germany; ^6^Department of Pediatrics, University of Düsseldorf, Düsseldorf, Germany; ^7^Department of Clinical and Molecular Medicine, Sapienza University of Rome, S. Andrea University Hospital, Rome, Italy; ^8^Institute of Social Medicine, Epidemiology and Health Economics, Charité-Universitätsmedizin Berlin, Berlin, Germany

**Correspondence**: Olympia Tsilochristou

*Clinical and Translational Allergy* 2016, **6(Suppl 2)**:P1

**Background**: The immunological differences between atopic and non-atopic IgE-responses have been gathering increasing interest. Many researchers support that related research questions should also focus on other antibody isotypes and especially the IgG production. Our group has been studying IgG responses in atopics versus non-atopics and preliminary results indicate that 2 year-old atopic children tend to develop slightly more frequently IgG responses to most environmental allergenic molecules and sources. The aim of the study is to investigate the longitudinal trend of this immunological difference in IgG production observed in atopic and non-atopic children at 2 years of age.

**Materials and methods**: Children of the German Multicenter Allergy Study (MAS) were included in the present analysis if they had provided: (1) ≥1 serum sample at age 1–3 years, (2) ≥2 serum samples at ages 5–7 years, (3) a serum sample at age 10 years. IgG (cut-off ≥0.1 ISU/L) and IgE (cut-off ≥0.3 ISU/L) to 91 molecules were tested by microarray (ISAC, TFS). Sera with undetectable IgE (<0.35 kU/L) against a panel of nine common foodborne and airborne allergenic extracts (ImmunoCAP, TFS) were considered negative for IgE against the panel of 91 molecules and were tested only for IgG. Atopy was defined as at least one positive IgE result against the panel of nine extracts.

**Results**: The mean prevalence of IgG production in non-atopics versus atopics was 41 and 47 % respectively (p value <0.001). Atopic children produced higher titers of IgG antibodies in comparison to non-atopic considering the geometric mean concentration (1.4 versus 1.1 kU/l, p = 0.013). This difference in the levels of IgG production was evident from the first year of life and remained up to the age of 10 years.

**Conclusions**: Atopic children produce longitudinally stronger and more frequent IgG responses in comparison to non-atopic in the MAS. Other factors such as nutritional aspects, the microbiome and/or parental history of atopy need to be taken into consideration to indicate reasons explaining these differences.

**Keywords**: IgG; IgE; Components; Molecular; MAS

#### P2 The IgG sensitization profiles against 112 allergenic components support the absence of a protective role of IgG in allergic individuals, outside of the context of SIT

##### Pol André Apoil^1^, Claire Mailhol^2^, Anne Broué-Chabbert^3^, Agnès Juchet^3^, Alain Didier^2^, Elodie Carrer^1^, Thomas Lanot^1^, Antoine Blancher^1^

###### ^1^Laboratoire d’Immunologie, hôpital Rangueil, Toulouse, France; ^2^Pneumo-Allergologie, hôpital Larrey, Toulouse, France; ^3^Pneumo-Allergologie, hôpital des enfants, Toulouse, France

**Correspondence**: Claire Mailhol

*Clinical and Translational Allergy* 2016, **6(Suppl 2)**:P2

**Background**: A significant increase of IgG (especially IgG_4_) anti-allergen antibodies is significantly associated with the success of SIT. However, in non-allergic subjects, the presence of IgG specific for allergens is well documented, and their role is controversial.

**Materials and methods**: We studied the IgG anti-allergen antibodies in 142 allergic patients with food and/or respiratory allergy symptoms and 53 control subjects, clinically asymptomatic and free of IgE sensitization, all living in the Southwest of France. After having determined the IgE sensitization profile by means of the ImmunoCAP ISAC™, we re-incubated the biochips with anti-human IgG polyclonal antibodies (Fc-specific) coupled to the AlexaFluor 647. The raw fluorescence values were converted to arbitrary units of IgG by means of a calibration serum having IgG reactivity toward 5 allergen components (nGal d3, rBla g 2, nArt v 3, nBos d lactoferrin, nGal d 1).

**Results**: We found that more than 80 % of individuals, allergic or not, have some IgG directed against egg white (nGal d1, d2), cow’s milk (nBos d5, d8), or one component from olive tree pollen (rOle e 9). For 32 components, allergic patients have concentrations of specific IgG significantly higher than non-allergic individuals (p < 0.001). Among the allergen components derived from the same organism, some are able to induce more frequently an IgE-sensitization, while other components mainly induce an IgG response, regardless of the glycosylation of these components.

**Conclusions**: Our results demonstrate that, in allergic patients, IgG sensitization is mostly directed against the same targets as that of IgE sensitization. This implies that, out of the context of SIT, IgG are most probably not able to protect allergic patients from IgE-mediated symptoms. In addition, we found that at least for some allergens (e.g. pollens from hazelnut, timothy-grass and olive tree), the selection of the immunoglobulin isotype during anti-allergen humoral responses is dependent on the allergen molecule itself (Table 2).

**Table 2:** Compared to non-allergic individuals, allergic patients have higher concentrations of specific IgG against 32 different allergen components

Allergen source	Type	Allergen component	Mean IgG (allergy) ^(a)(c)^	Mean IgG (no allergy)^(b)(c)^	*P* ^(d)^ (IgG)
Egg white	Food	nGal d 2	19.54	11.25	<0.0001
Egg white	Food	nGal d 3	5.83	2.03	<0.0001
Cow’s milk	Food	nBos d 5	14.78	6.69	0.00019
Fish	Food	rGad c 1	3.35	0.12	<0.0001
Hazelnut	Food	nCor a 9	4.85	2.22	0.00011
Nut	Food	nJug r 2	6.51	1.61	<0.0001
Peanut	Food	rAra h 2	2.99	0.28	<0.0001
Peanut	Food	nAra h 6	3.61	0.15	<0.0001
Soy	Food	nGly m 5	3.22	0.57	<0.0001
Wheat	Food	rTri a 14	5.43	1.38	<0.0001
Wheat	Food	nTri a aA_TI	6.61	2.22	<0.0001
Bermuda	Grass pollen	nCyn d 1	5.07	0.60	<0.0001
Timothy	Grass pollen	rPhl p1	3.75	1.32	<0.0001
Timothy	Grass pollen	rPhl p 4	4.52	0.62	<0.0001
Cedar	Tree pollen	nCry j 1	2.76	0.39	<0.0001
Cypress	Tree pollen	nCup a 1	4.27	0.51	<0.0001
Plane tree	Tree pollen	nPla a 2	3.61	0.95	<0.0001
Cat	Animal dander	rFel d 1	9.13	3.51	<0.0001
Dog	Animal dander	rCan f 1	2.96	0.34	0.00027
*D. farinae*	Mites	nDer f 1	2.44	0.78	<0.0001
*D. farinae*	Mites	rDer f 2	4.87	0.06	<0.0001
*D. pteron.*	Mites	nDer p 1	1.10	0.08	0.00021
*D. pteron.*	Mites	rDer p 2	1.80	0.02	<0.0001
Dog	Animal albumin	nCan f 3	3.51	1.13	0.00014
Peach	LTP (food)	rPru p 3	8.48	2.87	<0.0001
Mugwort	LTP (weed poll)	nArt v 3	2.97	0.33	<0.0001
Plane tree	LTP (tree poll)	rPla a 3	3.97	0.30	<0.0001
Birch	PR-10 (tree poll)	rBet v 1	6.37	0.26	<0.0001
Hazelnut	PR-10 (food)	rCor a 1.0401	2.94	0.37	<0.0001
Apple	PR-10 (food)	rMal d 1	4.42	0.44	<0.0001
Peach	PR-10 (food)	rPru p 1	2.37	0.10	<0.0001
Latex	Profilin	rHev b 8	4.11	0.63	0.0003

Undiluted sera from 195 individuals were studied by using the ImmunoCAP ISAC™ biochip. After having been used to measure specific IgE concentrations, the same biochips were then re-incubated with polyclonal anti-human IgG antibodies conjugated to alexa fluor 647. For the determination of specific IgG concentrations, the raw fluorescence data were converted to arbitrary IgG units by using calibration curves established from the IgG reactivity of a control serum against 5 components. Only allergen components for which IgG sensitization levels were significantly different between the sensitized/allergic and non-sensitized groups are shown. (**a**): group of 142 sensitized/allergic patients (sum of IgE ISAC Specific Units against ImmunoCAP ISAC allergens is above 5 ISU-E) recruited for food and/or respiratory allergy; mean age 20 (range 1–56), sex ratio 0.79. (**b**): group of 53 non-sensitized individuals, devoid of IgE-sensitization against any of the 112 components present on the ImmunoCAP ISAC biochip; mean age 30 (range 1–82), sex ratio 0.65. (**c**): IgG sensitization levels are expressed in arbitrary units (range 0–100 units). (**d**): p was calculated by the Mann–Whitney non-parametric test.

**Keywords**: Specific IgG; Specific IgE; Allergen components; Biochip

#### P3 The immune response against the timothy grass pollen allergen Phl p 5 in non-allergic humans

##### Almedina Kurtaj^1^, Christoph Hillebrand^1^, Gerda Fichtinger^1^, Martin Danzer^2^, Christian Gabriel^2^, Theresa Thalhamer^1^, Sandra Scheiblhofer^1^, Josef Thalhamer^1^, Richard Weiss^1^

###### ^1^University of Salzburg, Salzburg, Austria; ^2^Red Cross Blood Transfusion Service, Linz, Austria

**Correspondence**: Almedina Kurtaj

*Clinical and Translational Allergy* 2016, **6(Suppl 2)**:P3

**Background**: The current paradigm claims that tolerance induced via regulatory T cells is the major mechanism to maintain a non-allergic status in healthy individuals. Here, we examine a new hypothesis, which postulates that also non-allergic individuals mount antigen-specific immune responses against environmental antigens and that different immune response types exist and maintain this healthy condition. Furthermore, we hypothesize that depending on the living environment, non-allergic immune responses can be different. Hence, we assessed the immune status of non-allergic people living in a farming environment, who are regularly exposed to the major grass pollen allergen Phl p 5 in the context of a diverse microbial environment (animal sheds, haylofts, harvesting activities) and non-allergic people living in an urban environment, who lack such microbial exposure and diversity.

**Materials and methods**: Because of the low frequency of antigen-specific memory T cells in non-allergic donors, peripheral blood mononuclear cells were expanded antigen-specifically with rPhl p 5. After enrichment of antigen-specific memory T cells, proliferation markers, transcription factors, and cytokine secretion allowed identification of different T helper subsets like TH1, TH2, Treg, and TH17. Moreover, antigen-specific IgE, IgG1, IgG4, and IgA antibody levels in non-allergic humans were measured by ELISA.

**Results**: We could show that IFN-γ is the dominant cytokine after rPhl p 5 restimulation in non-allergic townspeople pointing to a TH1 biased immune response and we could also confirm this finding by staining of TH1 associated transcription factor T-bet. On the other hand, farmers displayed similar numbers of IL-10 and IFN-γ producing cells and as well a balanced expression of transcription factors FoxP3 and T-bet. Additionally, we detected significantly higher Phl p 5-specific IgG1 titers than IgG4 titers in both non-allergic groups. Interestingly, townspeople showed significantly increased Phl p 5-specific IgG4 titers and IgG seroconversion compared to famers.

**Conclusions**: In summary, it can be stated that tolerance induction is not the only mechanism to maintain a non-allergic state but rather that diverse antigen-specific immune response types are common in non-allergic individuals. Multiple mechanisms of naturally acquired protection exist and depending on the living environment different immune response types can establish and maintain a healthy non-allergic status.

**Keywords**: Grass pollen allergen; Environment; Antigen-specific immune response; Non-allergic immune response

#### P4 Analyzing the cross-reactivity profile of the major ragweed allergen Amb a 1

##### Martin Wolf^1^, Michael Hauser^1^, Ulrike Pichler^1^, Teresa Twaroch^2^, Gabriele Gadermaier^1^, Christof Ebner^3^, Hidenori Yokoi^4^, Toshiro Takai^5^, Alain Didierlaurent^6^, Adriano Mari^7^, Peter Briza^1^, Heidrun Behrendt^8^, Angela Neubauer^2^, Frank Stolz^2^, Fátima Ferreira^1^, Michael Wallner^1^

###### ^1^University of Salzburg, Salzburg, Austria; ^2^Biomay AG, Vienna, Austria; ^3^Allergieambulatorium am Reumanplatz, Vienna, Austria; ^4^Kyorin University, School of Medicine, Tokyo, Japan; ^5^Juntendo University, Graduate School of Medicine, Tokyo, Japan; ^6^Stallergens S.A., Antony, France; ^7^Associated Centers for Molecular Allergology, Rome, Italy; ^8^ZAUM, Center for Allergy and Environment, Munich, Germany

**Correspondence**: Michael Wallner

*Clinical and Translational Allergy* 2016, **6(Suppl 2)**:P4

**Background**: The major ragweed pollen allergen Amb a 1 has been classified as member of the pectate lyase allergen family. To date, five different Amb a 1 isoforms have been officially acknowledged by the IUIS allergen nomenclature subcommittee. Moreover, pectate lyases have been identified as major allergens within the pollen of mugwort, a weed species which together with ragweed belongs to the botanical order of Asteraceae, as well as within several trees belonging to the Cupressaceae order. Thus, we thought to investigate cross-reactivity pattern as well as sensitization profiles of Amb a 1 isoforms as well as related allergens from different sources.

**Materials and methods**: Pectate lyase pollen allergens from short ragweed (Amb a 1), mugwort (Art v 6), cypress (Cup a 1), mountain cedar (Jun a 1), and Japanese cedar (Cry j 1) were purified from aqueous pollen extracts. Moreover, three Amb a 1 isoforms (Amb a 1.01, 02, and 03, respectively) were either purified from pollen extracts or produced as recombinant proteins in *P. pastoris*. The allergens were characterized physico-chemically and thereafter IgE binding was assayed by immunoblot, ELISA, cross-inhibition, and mediator release assays.

**Results**: For cross-reactivity profiling of Amb a 1 with homologous pectate lyases we found that each of the four cohorts included in the study showed a distinct sensitization fingerprint, which reflected the natural allergen exposure of the patients. Moreover, we analyzed the IgE binding to different Amb a 1 isoforms using sera of Amb a 1 sensitized individuals from Central Europe. Within this cohort, we found that all three tested Amb a 1 isoforms were recognized by IgE to a similar extent.

**Conclusions**: Our data suggests that according to sensitization profiles pectate lyase allergens can be clustered into four categories, which are Amb a 1, Art v 6, Cup a 1/Jun a 1, and Cry j 1. Whereas cross-reactivity between Asteraceae and Cupressaceae allergens was limited, we found considerable cross-reactivity within each order. Moreover, there was no significant difference in IgE reactivity to the Amb a 1 isoforms 01, 02, and 03.

The work was supported by FWF project L688, CK-CARE AG Individual Project 2009-02, Biomay AG, the Christian Doppler Research Association, and Sparkling Science SPA 05-193.

**Keywords**: Ragweed; Pectate lyase allergen; Amb A 1; Cross-reactivity; Isoforms

#### P5 LTP (Pru p 3) sensitisation in skin prick test: which means in clinical practice?

##### Sara Carvalho, Tatiana Lourenço, Joana Cosme, Fátima Cabral Duarte, Amélia Spínola Santos, Ana Célia Costa, Manuel Pereira Barbosa

###### Immunoallergology Department, Hospital Santa Maria - Centro Hospitalar Lisboa Norte, Lisboa, Portugal

**Correspondence**: Fátima Cabral Duarte

*Clinical and Translational Allergy* 2016, **6(Suppl 2)**:P5

**Background**: Lipid Transfer Proteins (LTP) are present in plant foods and aeroallergens. They cause sensitisation mostly by gastrointestinal system and besides being associated with Oral Allergy Syndrome (OAS), LTP sensitisation is mainly related with severe and systemic reactions.

Our objective was evaluate the frequency of LTP(*Pru p 3*) sensitization in patients referenced to our Immunoallergology Department and correlate it with the pollens, latex and foods sensitisation and with the eventual relation with the severity of the allergic reactions.

**Materials and methods**: Retrospective observational study of 959 patients referred to the Immunoallergology Department during a period of 9 months, which were submitted to a standard battery of Skin Prick Tests (SPT). The population was characterized according to demographic data, average SPT wheal diameter and type of clinic presented. Statistical analysis: SPSS v20.

**Results**: From the 959 patients (36 % Male, 64 % Female, Median Age: 38 ± 18 years); 47 (5 %) presented LTP sensitisation. The patients were divided into two categories according to LTP sensitisation and characterized (see Table 3). In the SPT, the average LTP wheal diameter in patients that presented allergic reaction to rosacea was 8.7 mm (OAS), 7 mm (OAS and Systemic Symptoms) and 8.7 mm (Systemic Symptoms). We confirmed a statistically significate (*p* < 0.01) association between LTP and Pollen sensitisations, namely Grass, *Cynodon dactylon*, *Phleum pratense*, Plane Tree, *Parietaria judaica*, Olive Tree, *Artemisia vulgaris*, *Plantago lanceolata* and Latex. We found that given a greater number of pollen sensitisation, the greater the amount of patients with LTP sensitisations (from 0 to 6 pollens: 1.8, 4.0, 4.1, 11.3, 12.2, 22.7 and 41.7 % of sensitisation, respectively). There was no significant association (*p* = 0.015, trust interval of 99 %) between LTP and Profilin(*Pho d 2*) sensitisations.

**Table 3:** Characterization of the study population

Population	SPT—Positive LTP 5 % (n = 47)	SPT—Negative LTP 95 % (n = 912)
Gender		
Female	64 % (30)	64 % (587)
Median age	34 ± 11 years	38 ± 18 years
Medical history		
Rhinitis	78.7 % (37)	74.3 % (678)
Asthma	21.2 % (10)	28.3 % (259)
Eczema	14.8 % (7)	11.5 % (105)
Chronic Urticaria	14.8 % (7)	12.6 % (115)
Symptoms with latex	6.3 % (3)	0.5 % (5)
Food allergy		
Fresh Fruits—Rosacea	31.9 % (15)	1.8 % (17)
Fresh Fruits—Others	19.1 % (9)	2.3 % (21)
Peanut	10.6 % (5)	0.6 % (6)
Dry fruits	17.0 % (8)	0.9 % (9)
Clinic of Food Allergy to rosacea		
OAS	46.6 % (7)	58.8 % (10)
OAS + Systemic Symptoms	20 % (3)	5.8 % (1)
Systemic Symptoms	33.3 % (5)	35.2 % (6)
Pollen sensitization		
Wild Grasses	72.3 % (34)	32.6 % (298)
Grasses Grown	70.2 % (33)	28.9 % (264)
*Cynodon dactylon*	42.5 % (20)	14.5 % (133)
*Phleum pretense*	63.8 % (30)	25.6 % (234)
Plane tree	36.1 % (17)	5.5 % (51)
*Parietaria judaica*	31.9 % (15)	8.4 % (77)
Olive tree	57.4 % (27)	24.8 % (227)
*Artemisia vulgaris*	29.7 % (14)	6.3 % (58)
*Plantago lanceolata*	51.0 % (24)	19.7 % (180)
Latex sensitization	14.8 % (7)	2.4 % (22)
Profilin sensitization	10.6 % (5)	2.8 % (26)

**Conclusions**: In the LTP sensitised patients, we verified a greater sensitization to latex and foods, which occurred with a significantly greater frequency associated to rosacea fresh fruits. The LTP SPT wheal diameter wasn’t higher in patients with a more severe symptomatology; the gravity of reactions was similar between both LTP sensitised and non-sensitised patients. We also found a correlation between LTP and Pollen sensitisation (increase in the frequency of LTP sensitisation when the number of Pollen sensitisation is greater).

#### P6 IgE profiles, allergen exposure and lifestyle of 501 Austrian pupils: investigation of influences on the development of allergic sensitizations

##### Teresa Stemeseder^1^, Eva Klinglmayr^1^, Bettina Schweidler^1^, Lisa Lueftenegger^2^, Stephanie Moser^3^, Patrick Doppler^4^, Roland Lang^5^, Martin Himly^1^, Gertie J. Oostingh^2^, Arne Bathke^4^, Joerg Zumbach^3^, Thomas Hawranek^5^, Gabriele Gadermaier^1^

###### ^1^University of Salzburg, Department of Molecular Biology, Salzburg, Austria; ^2^Salzburg University of Applied Sciences, Biomedical Sciences, Salzburg, Austria; ^3^University of Salzburg, School of Education, Salzburg, Austria; ^4^University of Salzburg, Department of Mathematics, Salzburg, Austria; ^5^Paracelsus Medical University Salzburg, Department of Dermatology, Salzburg, Austria

**Correspondence**: Teresa Stemeseder

*Clinical and Translational Allergy* 2016, **6(Suppl 2)**:P6

**Background**: The reasons for the increasing number of patients suffering from allergic diseases are still not clear. In this study, we analyzed IgE-sensitization profiles, allergen exposure and lifestyle of 501 Austrian pupils aged 13–21 in order to shed light on influencing factors in the development of allergic sensitizations.

**Materials and methods**: A randomized cohort of 501 Austrian pupils from three geographical regions (alpine, urban, rural) donated capillary blood samples which were analyzed for IgE sensitization to 112 single allergens using the ImmunoCAP ISAC. To examine the exposure to indoor allergens from house dust mites, cats, dogs and molds, house dust samples were analyzed using a multiplex array. Demographic data, self-reported health status including allergies and other lifestyle conditions were collected using a detailed questionnaire.

**Results**: Fifty-seven percent of subjects declared to suffer from allergies including self-reported adverse reactions, while 21 % stated to have clinically confirmed allergies. IgE reactivity to any of the 112 molecules on the ISAC chip was observed in 53 % of subjects. Highest sensitizations were found to allergens from grass pollen (Phl p 1: 27 %), house dust mites (Der p 2/Der f 2: 18 %), tree pollen (Bet v 1: 16 %) and animal hair (Fel d 1: 14 %). The majority of subjects showed a complex sensitization profile, while exclusive mono-sensitizations to insect venom, mites or grass pollen were found in 35 % of sensitized subjects. IgE reactivity to pollen allergens is mainly driven by sensitization to grass pollen allergens in this region. Fel d 1 and Can f 1 were the most abundant allergens in the collected house dust samples. The sensitization rate to house dust mite was significantly higher in urban regions, whereas pupils in alpine regions were found to be less exposed to mite allergens which translated into lower IgE sensitization. Despite elevated levels of mite allergens found on farms, a decreased sensitization rate to Der p 2 was found in pupils living on farms compared to those living in flats. Lifestyle factors, such as smoking and cat ownership, as well as having allergic family members, showed a significant influence on the overall sensitization of individuals.

**Conclusions**: Distinct external triggers, such as living environment, dwelling form or allergen exposure, contribute to IgE development against distinct allergens and also to the onset of allergic diseases.

Supported by Sparkling Science, Federal Ministry of Science, Research and Economy, Austria.

**Keywords**: Sensitization profile; Allergen exposure; Lifestyle

#### P7 Molecular profiles of sensitization to perennial inhalant allergens in a middle European region

##### Petr Panzner^1^, Martina Vachova^1^, Tomas Vlas^1^, Marek Maly^2^

###### ^1^Charles University in Prague, Medical Faculty, Pilsen, Czech Republic; ^2^National Institute of Public Health, Prague, Czech Republic

**Correspondence**: Petr Panzner

*Clinical and Translational Allergy* 2016, **6(Suppl 2)**:P7

**Background**: The aim of our study was to assess the sensitization patterns to perennial inhalant allergens by means of molecular diagnostic approach in the region of Pilsen, Czech Republic.

**Materials and methods**: The microarray system ImmunoCAP ISAC has been used for specific IgE detection to 112 different molecules. Sera from 779 patients sensitized to at least one perennial inhaled allergen molecule were subject of analysis. These patients suffered from at least one of the following diagnoses: chronic rhinitis (73 %), bronchial asthma (41 %), atopic dermatitis (34 %), urticaria or angioedema (19 %) and/or anaphylaxis (11 %). Patient age ranged from 2 to 68 years, with a mean age of 25 years. The sex ratio was 45 % men to 55 % women.

**Results**: The most frequent sensitization rate was observed to cat-derived molecules (53.4 % overall), the most frequent being Fel d 1 (50.6 %). Sensitization to dog-derived molecules was slightly lower (38.4 % overall, Can f 5 26.0 % and Can f 1 22.1 %). The frequency of sensitization to at least an animal serum albumin (Fel d 2, Can f 3, Equ c 3, Bos d 6) was 6.6 %. This sensitization without sensitization to an animal species specific molecule was present only in 1.8 % of cases.

Almost the same sensitization rate as for cat was observed for mite-derived molecules (52.7 % overall, group 2 43.2 %, group 1 32.4 %). Isolated sensitizations to molecules derived from storage mites Lep d 2 and/or Blo t 5 without sensitization to other mite-derived molecules were observed only exceptionally (0.9 %). Similarly, sensitization to at least one cockroach specific molecule (Bla g 1, 2, 5) was very rare (in 1.0 %). Sensitization to a tropomyosin (Der p 10 and/or Bla g 7) was observed in 3.6 %. Co-sensitization of Der p 10 with other mite-derived molecules was observed in 1.2 %.

The frequency of sensitization to mold-derived molecules was 33.4 %, the most frequent being Alt a 1 (26.8 %). Sensitization to Aspergillus derived molecules (Asp f 1 and/or Asp f 3 and/or Asp f 6) was 6.9 %.

**Conclusions**: Molecular diagnosis of allergy gives a more precise and comprehensive insight into perennial inhalant allergen sensitization patterns than extract-based testing, allowing for better understanding of the sensitization process and regional differences. The data presented may help to improve the diagnostic and treatment procedures, especially focusing the need to quantify the content of the most frequent sensitizing molecules in the diagnostic and therapeutic allergen extracts used in the respective region.

**Keywords**: Sensitization; Epidemiology; Perennial inhalant allergens; Microarray; Molecular diagnostics

#### P8 Evolution of the IgE response to house dust mite allergen molecules in childhood

##### Daniela Posa^1^, Serena Perna^1^, Stephanie Hofmaier^1^, Laura Hatzler^1^, Alexander Rohrbach^1^, Carl-Peter Bauer^2^, Ute Hoffmann^2^, Johannes Forster^3^, Fred Zepp^4^, Antje Schuster^5^, Philippe Stock^6^, Ulrich Wahn^1^, Linus Grabenhenrich^7^, Thomas Keil^7^, Susanne Lau^1^, Kuan-Wei Chen^8^, Yvonne Resch^8^, Susanne Vrtala^8^, Rudolf Valenta^8^, Paolo Maria Matricardi^1^

###### ^1^Department of Paediatric Pneumology & Immunology, Charité-Universitätsmedizin Berlin, Berlin, Germany; ^2^Department of Pediatrics, Technical University of Munich, Munich, Germany; ^3^Department of Pediatrics St Hedwig, St Josefs Hospital, Freiburg, Germany; ^4^Department of Pediatrics and Adolescent Medicine, University Medicine Mainze, Mainz, Germany; ^5^Department of Pediatrics, Heinrich-Heine-University, Düsseldorf, Germany; ^6^Department of Paediatric Pneumology & Immunology, Altonaer Kinderkrankenhaus, Hamburg, Germany; ^7^Institute for Social Medicine, Epidemiology and Health Economics, Charité - Universitätsmedizin Berlin, Berlin, Germany; ^8^Division of Immunopathology, Department of Pathophysiology and Allergy Research, Center of Pathophysiology, Infectiology and Immunology, Medical University of Vienna, Vienna, Austria

**Correspondence**: Daniela Posa

*Clinical and Translational Allergy* 2016, **6(Suppl 2)**:P8

**Background**: Recent studies have shown that the allergic IgE response to grass pollen starts years before disease onset as a weak monosensitization or oligosensitization phenomenon and increase in serum concentration and complexity through a “molecular spreading” process during preclinical and early clinical disease stages. We aimed this study to investigate whether the development of the IgE response to house dust mite allergen molecules follows similar rules.

**Materials and methods**: We have taken advantage of the data of Multicenter Allergy Study, a prospective long-term observational birth cohort study, started in 1990 and recruiting 1314 infants born in 5 German cities. Blood samples were collected at 1, 2, 3, 5, 6, 7, 10, 13 and 20 years of age. Sera with IgE antibodies to an extract of *Dermatophagoides pteronyssinus* were further tested for the presence of IgE to Der p 1, Der p 2, Der p 4, Der p 5, Der p 7, Der p 11, Der p 14, Der p 15, Der p 18, Der p 21, Der p 23, Clone 16.

**Results**: Overall, 191 subjects with a IgE response to D.pt. at least once in life could be examined. The molecules most frequently recognized by IgE were Der p 2, Der p 1 and Der p 23, in 147, 117 and 97 subjects, respectively. The mean age at first detection of a positive response was inversely related to the frequency of detection to each individual molecule: respectively 8.7 (±0.70) (Der p 2), 8.9 (±0.85) (Der p 1) and 9.6 (±0.98) (Der p 23). All the other allergens were detected in less than 50 % of the cases. The profile of IgE sensitization to all the 12 examined allergenic molecules was extremely heterogeneous in the study population. Accordingly, 41 children responded to only one molecule during the whole observation period (monomolecular response), 67 responded to 2–4 molecules (oligomolecular), and 60 responded to ≥5 molecules (polymolecular). The maximum number of molecules recognized by IgE was inversely related to the age at first detection of an IgE response to D.pt.

**Conclusions**: Der p 1, Der p 2 and Der p 23 are the major allergenic molecules in the MAS birth cohort study. A molecular spreading in the IgE response was observed in most but not all mite-sensitized children, and the IgE response to mites is greatly heterogeneous at molecular level. Early sensitization is associated with polymolecular sensitization. The hypothesis that different sensitization profile and trends underlies different responses to AIT deserves to be tested.

**Keywords**: IgE antibodies; Childhood; Dermatophagoides pteronyssinus

#### P9 Tropomyosin (Pen a1): to include or not to include in skin prick testing?

##### Joana Cosme^1^, Sara Carvalho^1^, Tatiana Lourenço^1^, Amélia Spínola Santos^1^, Manuel Pereira Barbosa^2^

###### ^1^Immunoallergy Department - Hospital de Santa Maria – Centro Hospitalar Lisboa Norte, Lisbon, Portugal, Lisbon, Portugal; ^2^Immunoallergy Department - Hospital de Santa Maria – Centro Hospitalar Lisboa Norte, Lisbon, Portugal; Faculdade de Medicina de Lisboa, Lisbon, Portugal

**Correspondence**: Joana Cosme

*Clinical and Translational Allergy* 2016, **6(Suppl 2)**:P9

**Background**: Tropomyosin (*Pen a 1*) is a pan-allergen. The homology between tropomyosins from mites, crustaceans, molluscs, insects and nematodes is responsible for the cross-reactivity between them. Although *Pen a 1* in vitro sensitization has been described, its in vivo sensitization has been poorly characterized.

**Objectives**: Study the prevalence of *Pen a 1* sensitization by skin prick test (SPT) and its relation with a precise history of crustacean and/or molluscs allergy, in a population of patients with respiratory allergy sensitized to mites.

**Materials and methods**: Data regarding the 1030 patients followed during 9 months in an Allergy outpatient clinic were retrospectively analysed. Demographic and clinical information and the results of SPT—hospital standard panel for inhalants, shrimp extract and *Pen a 1* (Leti^®^) were collected. Positive result defined as a wheal diameter ≥3 mm. Data analysis using STATA 13.

**Results**: There were included 450 patients with respiratory allergy (56 % rhinitis, 8 % asthma, 35 % both). All patients had sensitization to mites and were skin tested with a shrimp and *Pen a 1* extracts. All the comparisons were made between patients with or without *Pen a 1* and shrimp extract sensitization. *Pen a 1* SPT has higher specificity (96.67 %) and negative predictive value (41.67 %) in the confirmation of clinical crustacean and/or molluscs allergy. On the other hand, shrimp extract showed a higher *sensitivity* (53.33 %). With regard to the degree of concordance between shrimp and *Pen a 1* sensitization, the percentage of agreement were 90 % (majority of discordant pairs: shrimp+/tropomyosin−), *Cohen’s kappa* coefficient was 0.39 (moderate concordance).

**Conclusions**: Although the percentage of *Pen a 1* sensitized patients is small, these patients have higher cutaneous reactivity to mites (wheals with higher diameter). *Pen a 1* sensitized patients have clinical history of crustacean and/or molluscs allergy more frequently than patients without *Pen a 1* sensitization or with shrimp sensitization. The higher specificity of *Pen a 1* SPT makes it an important tool for diagnostic confirmation of clinical allergy to crustacean and/or molluscs, in patients with respiratory allergy sensitized to mites.

**Keywords**: Tropomyosin; Skin prick test; Sensitization

#### P10 Component-resolved IgE profiles in Georgian patients

##### Tamar Abramidze, Nino Lomidze, Maia Gotua

###### Center of Allergy & Immunology, Tbilisi, Georgia

**Correspondence**: Nino Lomidze

*Clinical and Translational Allergy* 2016, **6(Suppl 2)**:P10

**Background**: Component-resolved diagnostics (CRD) is becoming of growing importance in clinical investigation of IgE-mediated allergies, which allows a comprehensive analysis of individual sensitization profiles with multiplexed purified and recombinant allergens. We studied the sensitization pattern in Georgian allergic patients.

**Materials and methods**: Allergic patient’s sera samples were tested by a microarray technology (ImmunoCAP ISAC, Thermo Fisher Scientific, ImmunoDiagnostics, Uppsala, Sweden) and specific IgE levels against 112 different allergen components were detected.

**Results**: The study population, comprising 81 allergic patients, included 26 adults (mean age 36.2 ± 10.9) and 55 children (mean age 6.1 ± 4.58). Polysensitization was seen in 84.6 % of subjects. Among investigated mainly species-specific food components IgE reactivity to nGal d 1, nGal d 2 and nBos d 8 was more prevalent and represented 11.3, 10.1 and 10.1 % correspondently. Concerning pollens, specific IgE antibodies against following aeroallergen components were detected more frequently: 1) grass pollen—nCyn d 1 (35.4 %), rPhl p 1 (35.4 %) and nPhl p 4 (34.1 %); 2) tree pollen—nCup a 1 (26.5 %); 3) weed pollen—nAmb a 1 (21.5 %). Main allergen component from the mold group was rAlt a 1 (10.1 %) and animal group was rFel d 1 (12.6 %). Among house dust allergen components sIgE to rDer f 2, nDer p 1 and rDer p 2 showed higher positive rates. PR-10 reactivity was detected in 56 patients (45 %), Profilin reactivity in 53 (43 %) and LTP in 36 (29 %).

**Conclusions**: The sensitization pattern specific for Georgian population was defined by CRD, which helps to identify potential disease-eliciting molecules, predict cross-reactivity, severity of reactions and the probability of the development of tolerance.

**Keywords**: Sensitization; Diagnostics; Allergen; Component

#### P11 Cross reactivity between food and pollen allergens in Lithuania according to spIgE evaluation

##### Austeja Dapkeviciute^1^, Ruta Einikyte^1^, Jolita Norkuniene^2^, Laima Skrickiene^3^, Asta Miskiniene^4^, Violeta Kvedariene^5^

###### ^1^Faculty of Medicine, Vilnius University, Vilnius, Lithuania; ^2^Department of Mathematical Statistics, Vilnius Gediminas Technical University, Vilnius, Lithuania; ^3^Public institution “Centro poliklinika”, Vilnius, Lithuania; ^4^JSC “Imunodiagnostika”, Didzioji Riese, Lithuania; ^5^Clinic of Pulmonology and Allergology, Vilnius University, Vilnius, Lithuania

**Correspondence**: Austeja Dapkeviciute

*Clinical and Translational Allergy* 2016, **6(Suppl 2)**:P11

**Background**: Cross reactivity syndrome is an increasing problem among allergic patients. Most commonly cross-reactivity appears between food and pollen. The aim of our study was to determine the most common food and pollen allergen cross reactivity in Lithuanian population.

**Materials and methods**: A retrospective study was conducted between 2013 and 2014 in public institution “Centro Poliklinika”. Data of 510 patients with suspicion of food or pollen allergy was analysed. We included 82 adults, age median 32 years [26–39], and 428 children, age median 5 years [4–8]. 262 (51.4 %) patients were men and 248 (48.6 %) women. All patients were tested with OPTIGEN^®^ mix 36 panel (Hitachi Chemical Diagnostics, Inc. U.S.A) detecting *allergen*-*specific immunoglobulin E* (*IgE*) *for 36 food and inhalant allergens.* A patient was considered allergic if allergen specific immunoglobulin E (spIgE) was more than 2 class.

**Results**: We found a strong correlation between having food and pollen allergy (correlation coefficient 0.538, p < 0.001). Very strong correlation was between nettle and orange (correlation coefficient 0.914, p < 0.001) and rape and tomato (correlation coefficient 0.738, p < 0.001). Strong correlation was found between mugwort and barley (correlation coefficient 0.662, p = 0.005), hazelnut and nettle (correlation coefficient 0.638, p = 0.019) and between birch and apple (correlation coefficient 0.636, p = <0.001).

**Conclusions**: We found very strong correlation between nettle and orange and rape and tomato in Lithuanian population. Also strong correlation was found between mugwort and barley, hazelnut and nettle and well known correlation between birch and apple.

**Keywords**: Cross reactivity; Food; Pollen

#### P12 Distribution of inhalant allergy in the population of Lithuania

##### Ruta Einikyte^1^, Austeja Dapkeviciute^1^, Jolita Norkuniene^2^, Laima Skrickiene^3^, Asta Miskiniene^4^, Violeta Kvedariene^5^

###### ^1^Faculty of Medicine, Vilnius University, Vilnius, Lithuania; ^2^Department of Mathematical Statistics, Vilnius Gediminas Technical University, Vilnius, Lithuania; ^3^Public institution, Vilnius, Lithuania; ^4^JSC „Imunodiagnostika“, Didzioji Riese, Lithuania; ^5^Clinic of Pulmonology and Allergology, Vilnius University, Didzioji Riese, Lithuania

**Correspondence**: Ruta Einikyte

*Clinical and Translational Allergy* 2016, **6(Suppl 2)**:P12

**Background**: **S**ensitization to inhalant allergens is an increasing problem around the world as well as in Lithuania. The aim of this study was to evaluate the distribution patterns of allergy to inhalant allergens in the population of Lithuania.

**Materials and methods**: A retrospective study was conducted in 2013–2014 year period in public institution “Centro Poliklinika” involving 664 patients of whom 49.5 % (n = 329) were male and 50.5 % (n = 335)—female. Patients with suspicion of allergy to inhalants were tested for allergen—specific immunoglobulin E (IgE) using OPTIGEN^®^ mix 36 and inhalant 36 panels (Hitachi Chemical Diagnostics, Inc. U.S.A). A patient was considered allergic when spIgE to an allergen was more than class 2.

**Results**: In our study 31.0 % (n = 206) of participants were allergic to at least one inhalant allergen. 76.7 % (n = 158) of them were children, age median was 5.0 [4.0–8.0] years, allergic adults encompassed 23.3 % (n = 48), their age median was: 32.0 [26.0–39.0] years. 15.7 % (n = 104) of allergic patients were allergic to pollen. Among patients allergic to pollen, the most common allergens were timothy grass 59.6 % (n = 62), birch 44.2 % (n = 46), mugwort 29.8 % (n = 31). 16.2 % (n = 111) of all allergic patients were allergic to house dust mites with no statistical differences between *D. farinae* and *D. Pteronyssinus*. The allergy to molds or epidermal allergens were less common.

**Conclusions**: In the population of Lithuania inhalant allergy was confirmed to one third of tested patients. Allergy to house dust mites and pollen were dominant. Timothy grass, mugwort and birch were the most important in pollen allergy.

**Keywords**: Inhalant; Allergens

### Poster Session 2: Allergen molecules: identification, characterization, structure and function

#### P13 Interference of antigen 5-based cross-reactivity in the diagnosis of hymenoptera venom allergy

##### Maximilian Schiener^1^, Bernadette Eberlein^2^, Carmen Moreno-Aguilar^3^, Gunilla Pietsch^2^, Mareike Mc Intyre^2^, Lea Schwarze^4^, Dennis Rußkamp^1^, Tilo Biedermann^2^, Edzard Spillner^5^, Ulf Darsow^2^, Carsten Schmidt-Weber^1^, Markus Ollert^6^, Simon Blank^1^

###### ^1^Center of Allergy and Environment (ZAUM), Technical University of Munich and Helmholtz Center Munich, Munich, Germany; ^2^Department of Dermatology and Allergy Biederstein, Technical University of Munich, Munich, Germany; ^3^Hospital Universitario Reina Sofia, Córdoba, Córda, Spain; ^4^Institute of Biochemistry and Molecular Biology, University of Hamburg, Hamburg, Germany; ^5^Immunological Engineering, Depaartment of Engineering, Aarhus University, Aarhus, Denmark; ^6^Department of Infection and Immunity, Luxembourg Institute of Health (LIH), Esch-Sur-Alzette, Luxembourg

**Correspondence**: Maximilian Schiener

*Clinical and Translational Allergy* 2016, **6(Suppl 2)**:P13

**Background**: Allergies due to the venoms of hymenoptera can cause severe systemic reactions. In spite of the progress of component-resolution in the last years, diagnosis as well as therapy of venom allergy is still challenging. Amongst others this is due to extensive cross-reactivity between different venoms. In this study the antigens 5 of 7 hymenoptera species were recombinantly produced, characterized in detail and their cross-reactivity analyzed.

**Materials and methods**: The antigens 5 Ves v 5, Vesp c 5, Pol d 5, Pol a 5, Dol m 5, Sol i 3 and the potentially hypoallergenic Poly s 5 were recombinantly produced in insect cells. The resulting purified proteins were characterized by immunoblotting und structural models were generated. Moreover, sera of venom allergic patients were assessed for IgE cross-reactivity and additionally basophil activation tests were performed.

**Results**: All antigens 5 were successfully produced in Sf9 insect cells. As expected from sequence alignments structural models reveal identical folding, although surface charges differ between the different molecules. However, due to the use of Sf9 cells as expression host all antigens 5 were devoid of carbohydrate-based cross-reactivity. The analysis of sera from Ves v 5-reactive and Polistes allergic patients revealed extensive cross-reactivity of all antigens 5, independent of glycosylation. Some sera showed distinct reactivity profiles with the diverse antigens 5 and others reacted with all of them. Additionally, basophil activation tests from Ves v 5-reactive patients could reveal the same cross-reactivity with all antigens 5. This indicates the presence of shared as well as of individual IgE epitopes.

**Conclusions**: The comparative analysis of antigens 5 from 7 different hymenoptera species revealed extensive cross-reactivity between all allergens on protein level. This implicates that antigens 5 are inappropriate marker allergens for the diagnostic discrimination between antigen 5-containing venoms. Moreover, detailed analyses on a molecular level can contribute to elucidate the clinical impact in the observed cross-reactivity.

#### P14 IgE cross-reactivity between European Hymenoptera and Asian hornet (*Vespa velutina*) venom allergens

##### Cyril Longé^1^, Andrea Brazdova^1^, Jean-Louis Brunet^2^, Claire Schwartz^3^, Bruno Girodet^2^, François Lavaud^4^, Joelle Birnbaum^5^, Nhân Pham Thi^6^, Magalie Duchateau^6^, Julia Chamot-Rooke^6^, Laurence Guilloux^7^, Marie-Ange Selva^1^, Rémy Couderc^1^, Hélène Sénéchal^1^, Jean-Pierre Sutra^1^, Pascal Poncet^1^

###### ^1^Hopital Trousseau, Paris, France; ^2^SFA hymenoptera task force, Lyon, France; ^3^SFA hymenoptera task force, Toulouse, France; ^4^SFA hymenoptera task force, Reims, France; ^5^SFA hymenoptera task force, Aix-En-Provence, France; ^6^Institut Pasteur, Paris, France; ^7^Biomnis, Lyon, France

**Correspondence**: Pascal Poncet

*Clinical and Translational Allergy* 2016, **6(Suppl 2)**:P14

**Background**: Venoms of hymenoptera are responsible of 1/3 of anaphylactic shocks and 17 allergens have been described in the IUIS data bank in various hymenoptera species including *Apis*, *Vespa*, *Vespula*, *Dolichovespula* and *Polistes*. In 2004 a new sub species of *Vespa*, *Vespa velutina*, the yellow-leg asian hornet, was introduced in south west of France and is now present in more than 75 % of the French territory quickly progressing towards north and east of France. These hornets are bee-killers and build their nest close to human houses. Thus they represent a risk factor for biodiversity as well as for human health because their stings result in a huge loco-regional or systemic reaction. Some anaphylactic shocks have been reported.

**Materials and methods**: In order to unravel the proteins and allergenic repertoire of the venom of asian hornet, an immunoproteomic study was designed.

**Results**: A protein extract from 20 venom sacs was performed and run in 1 or 2-dimensional (1 or 2D) gel electrophoresis. Western blotting using sera from patients diagnosed allergic to the venom of european hymenoptera (bees, yellow jackets or hornets) revealed numerous IgE reactive bands demonstrating that individuals, allergic to european hymenoptera venom, have IgE recognizing asian hornet venom proteins. Mass spectrometry analysis of picked protein spots in 2D experiments, followed by data bank searches, allowed the identification of allergens homologous to already known allergens in european hymenoptera venom (*Vespa magnifica* and *affinis*, *Apis mellifera*, *Dolichovespula maculata,* *Vespula vulgaris* and *germanica* and *Polybia paulista*) such as phospholipase A1, antigen 5, arginine kinase, hyaluronidase and dipeptidyl di peptidase IV.

**Conclusions**: In conclusion the data show that some cross reactivities exist between European hymenoptera and asian hornet venom allergens. Whether specific unique allergens are present in the *Vespa velutina* venom is under investigation. The results will improve the diagnosis of allergy to hymenoptera venom and help in designing the appropriate extract for a specific immunotherapy of patient allergic to asian hornet.

**Keywords**: Hymenoptera asian hornet

#### P15 Carbohydrate composition of house dust mite extracts and major group 1 and group 2 allergens

##### Steffen Augustin^1^, Linda Pump^1^, Martin Wald^1^, Thomas Eichhorn^2^, Frank Fischer^2^, Christoph Willers^1^

###### ^1^Allergopharma GmbH & Co KG, Reinbek, Germany; ^2^Merck KGaA, Darmstadt, Germany

**Correspondence**: Steffen Augustin

*Clinical and Translational Allergy* 2016, **6(Suppl 2)**:P15

**Background**: Glycosylation plays an important role in the recognition and uptake of allergens by antigen presenting cells (APCs) and the modulation of immune responses. In addition, cross-reactive carbohydrate determinants (CCDs) constitute epitopes for human IgE, although their recognition seems not to be associated with clinical symptoms. Thus, elucidation of carbohydrate structures and investigation of their biological function is essential, to understand immunological properties of natural allergens.

**Materials and methods**: *D. pteronyssinus* and *D. farinae* extracts and natural house dust mite major allergens were assessed for the presence and identity of glycans by Periodate-Schiff staining, detection by c-type lectins, carbohydrate-specific antibodies and mass spectrometry.

**Results**: Glycan structures were detected by Periodate-schiff staining to be present on high molecular weight proteins in mite extracts from both species. Binding of the lectins GNA, PNA and DSA to many of these glycan-carrying proteins indicates the presence of N-linked high mannose and O-linked glycans comprising a core GalGalNAc_1_. Investigation by a 1,3-fucose-specific antibody excluded the presence of the CCD 1,3-fucose in the investigated samples. Applying mass spectrometry, the presence of various N-linked high mannose structures linked to a core HexNAc_2_ glycan was confirmed. Analyses of purified natural major group 1 and group 2 allergens showed that group 1 allergens from *D. pteronyssinus* and *D. farinae* can carry the N-glycans HexNAc_1_, HexNAc_2_ and HexHexNAc in a predicted N-glycosylation consensus sequence. Furthermore, our analyses demonstrate that group 2 allergens from both species can carry at four homologous sites modifications by hexose monosaccharides and in addition at one of these residues a polyhexose.

**Conclusions**: Our results reveal a complex glycosylation pattern of proteins in *D. pteronyssinus* and *D. farinae* extracts that presumably influences significantly the allergenic and immunogenic properties of house dust mite proteins. The immunological relevance of the individual identified carbohydrate modifications needs to be elucidated in detail in further studies. Our data could contribute to development of innovative approaches in allergen specific immunotherapy.

**Keywords**: Carbohydrate; Mite; Glycan; Allergen

#### P16 Specificity of monoclonal antibodies against cross-reactive carbohydrate determinants

##### Michaela Miehe^1^, Melanie Plum^1^, Sara Wolf^1^, Frederic Jabs^2^, Tim Raiber^1^, Frank Bantleon^1^, Henning Seismann^3^, Thilo Jakob^4^, Edzard Spillner^1^

###### ^1^Aarhus University, Aarhus, Denmark; ^2^University of Hamburg, Hamburg, Germany; ^3^Euroimmun AG, Lübeck, Germany; ^4^University Medical Center Freiburg, Freiburg, Germany

**Correspondence**: Michaela Miehe

*Clinical and Translational Allergy* 2016, **6(Suppl 2)**:P16

**Background**: Core structures of N-glycans modified with α1,3-fucose and β1,2-xylose represent the most frequently recognized IgE epitope. The lack of specific antibodies and the complexity of N-glycosylations so far hampered molecular insights into this important interaction. Structural characteristics as well as fine specificity of antibodies against such carbohydrates remain largely unclear.

**Materials and methods**: Core glycan-specific IgE and Fab antibodies were produced by baculovirus-mediated infection of insect cells or in mammalian cells. Functionality and affinity of resulting antibodies was shown by ELISA and SPR analyses. Fab antibodies were used for crystallisation and structure determination in complex with an epitope disaccharide. Fine specificity and potential cross-reactivity with carbohydrate antigens was assessed using glycan arrays.

**Results**: Recombinant IgE and Fab antibodies exhibited pronounced immunoreactivities in ELISA and dissociation constants in the nanomolar range. Structural analyses of a Fab in complex with the disaccharide epitope surrogate provided evidence for establishment of the IgE epitope by several antibody residues interacting with the core fucose. Glycan arrays that include a variety of synthetic carbohydrates representing the most frequently identified binding epitopes proved the specificity of the interaction of core-glycan-specific antibodies.

**Conclusions**: In summary, our data provide insights into the specificity of recognition of complex N-glycans in pathological conditions.

#### P17 Red meat allergic patients have a selective IgE response to the a-Gal glycan

##### Danijela Apostolovic^1^, Anh Thu Tran^2^, Sara Sanchez-Vidaurre^2^, Tanja Cirkovic Velickovic^3^, Maria Starkhammar^4^, Carl Hamsten^2^, Marianne Van Hage^2^

###### ^1^Faculty of Chemsitry, University of Belgrade, Belgrade, Serbia; ^2^Karolinska Institutet, Stockholm, Sweden; ^3^Faculty of Chemistry, University of Belgrade, Belgrade, Serbia; ^4^Department of Internal Medicine, Södersjukhuset, Stockholm, Sweden

**Correspondence**: Danijela Apostolovic

*Clinical and Translational Allergy* 2016, **6(Suppl 2)**:P17

**Background**: Many allergens are glycoproteins that carry one or several carbohydrates linked to the protein structure. Not only proteins but also carbohydrates can stimulate the production of IgE antibodies and be strong inducers of Th2 responses. Galactose-α-1,3-galactose (α-Gal) is a mammalian carbohydrate with significance in a novel type of food allergy. Patients with IgE against α-Gal report severe allergic symptoms 3 to 6 hours after red meat consumption. We investigated whether IgE from red meat allergic patients recognize other mammalian glycans than α-Gal or glycans from the plant kingdom and venoms of importance in allergy. Furthermore, we examined whether red meat allergic patients’ IgE response is directed against the carbohydrate-protein complex or against the glycan part only.

**Materials and methods**: Sera from 24 red meat allergic patients were analyzed on ImmunoCAP for IgE against α-Gal, MUXF3, nCup a 1, nArt v 1 and with Streptavidin ImmunoCAP for biotinylated-Gal-α1,3-Gal, biotinylated-Neu5Gc, as well as enzymatically deglycosylated biotinylated-Gal-α1,3-Gal. The absence of the α-Gal epitope after enzymatic deglycosylation was identified and verified by an anti-α-Gal monoclonal antibody, IgE from red meat allergic patients by IgE-immunoblot as well as α-Gal-IgE inhibition experiment.

**Results**: We found that all 24 red meat allergic patients neither had an IgE antibody response against the other abundant mammalian glycan N-glycolylneuraminic acid nor against cross-reactive carbohydrate determinants from plant or venom sources (nCup a 1, nArt v 1 and MUXF3). Deglycosylation of an α-Gal containing protein, bovine thyroglobulin, significantly reduced the IgE response.

**Conclusions**: We show that red meat allergic patients have a selective IgE response to the α-Gal glycan found in red meat. Other common glycans reactive in allergic disease are not targets of red meat allergic patients’ IgE.

**Keywords**: a-Gal; Cross-reactive carbohydrate determinant (CCD); Glycan; IgE; Red meat allergy

#### P18 Specificity of non-specific lipid transfer proteins and influence of the ligands on their three-dimensional structure

##### Pawel Dubiela^1^, Piotr Humeniuk^1^, Sabine Pfeifer^1^, Merima Bublin^1^, Tomasz Borowski^2^, Karin Hoffmann-Sommergruber^1^

###### ^1^Medical University of Vienna, Vienna, Austria; ^2^The Jerzy Haber Institute of Catalysis and Surface Chemistry of the Polish Academy of Sciences, Krakow, Poland

**Correspondence**: Pawel Dubiela

*Clinical and Translational Allergy* 2016, **6(Suppl 2)**:P18

**Background**: Plant non-specific lipid transfer proteins (nsLTPs) are relevant food allergens e.g. from peach (Pru p 3), hazelnut (Cor a 8) or walnut (Jug r 3). They share a conserved fold with an internal cavity. Different lipid–protein complexes showed that the tunnel adapts its volume while binding a broad range of hydrophobic molecules.

**Materials and methods**: The probe 1-anilinonaphthalene-8-sulfonic acid (1,8-ANS) is non-fluorescent in water but fluorescent with a maximum emission wavelength at 456 nm when binding to the hydrophobic cavity of nsLTPs. Pru p 3, Cor a 8 and Jug r 3 were incubated with different fatty acids (lauric, stearic, oleic acid) and total lipid extract from hazelnut. The binding of lipids was monitored by adding 10 µM 1,8-ANS and measuring the decrease of 1,8-ANS fluorescence. Furthermore, molecular dynamic (MD) analysis was applied to explore the nature of interaction between nsLTPs and tested ligands. ptraj analysis was used to process MD trajectories and to provide information about RMS deviation from a reference structure, hydrogen bonding, time-correlation functions and diffusional behavior.

**Results**: Due to pre-incubation of Pru p 3 with lipids a concentration dependent reduction of ANS binding was observed. Pru p 3 incubated (1:1; 1:10) with lauric acid showed 19 and 66 % of ANS fluorescence reduction, respectively, compared with Pru p 3 without lipids. For oleic acid (1:1; 1:10) reduction was 7 and 77 %, respectively. For other tested lipids and nsLTPs, this reduction was not seen. Molecular dynamic analysis suggests changes in protein structure due to binding of certain ligands. Interaction of Pru p 3 with oleic acid moved the C-terminal loop out towards the surface of the molecule. This interaction is stabilized by hydrogen bonds with Arg32. Interestingly, the region affected by conformational changes is one that was shown to be one of the main epitopes of Pru p 3.

**Conclusions**: In this study, we observed that Pru p 3 was able to bind lauric and oleic acid, while other lipids did not interact. Molecular dynamic simulation has proved differences in the binding capacity of Pru p 3 with the ligands that can lead to conformational changes. The allergen–lipid interaction may act as a potential danger signal during the allergic sensitization phase or increase allergenicity during the effector phase. However, this remains to be investigated.

**Keywords**: Non-specific lipid transfer proteins; Protein–ligand interaction; 3D structure

#### P19 Real-time PCR analysis of Pru av 1 and Pru av 3 allergens

##### Martie C. M. Verschuren^1^, Shanna Bastiaan-Net^2^, Defien Depoortere^1^, Kay Foetisch^3^, Stephan Scheurer^3^, Harry J Wichers^2^, Theo Noij^1^

###### ^1^Avans University of Applied Science, School of Life Science and Environmental Technologies, Research group Analysis Techniques in Life Science, Breda, The Netherlands; ^2^Wageningen UR Food & Biobased research, Wageningen, Netherlands, The; ^3^Division of Allergology, Paul-Ehrlich Institut, Langen, Germany

**Correspondence**: Martie C. M. Verschuren

*Clinical and Translational Allergy* 2016, **6(Suppl 2)**:P19

**Background**: Food allergy to sweet cherry is very common throughout Europe. Oral reactions observed in people from central and northern parts of Europe are mainly caused by Pru av 1 (Bet v 1-like) allergens, whereas systemic reactions, more common in the Mediterranean region, are caused by the Pru av 3 (nsLTP) allergen. Hypoallergenic cherry cultivars might be identified by analysing gene expression levels of Pru av 1 and its isoallergens or variants, and Pru av 3 in natural occurring cherry cultivar variations. However, no RT-qPCR assays are available so far. Therefore, we designed different RT-qPCR primer sets for Pru av 1 and Pru av 3 gene expression studies.

**Materials and methods**: Coding sequences of Pru av 1.0101, three Pru av 1.02 variants, and the Pru av 3 coding sequence were cloned in a pET16b vector. Constructs were verified by sequencing, and plasmid DNA was isolated. Primer sets were validated for specificity using different plasmid DNA samples with real-time quantitative PCR experiments using SybrGreen I chemistry on a LightCycler 48 instrument.

**Results**: PCR amplification curves were obtained using the designed primer sets. Primer set 1 showed specific amplification of isoallergen Pru av 1.0101. Primer set 2 amplified the three variants of isoallergens Pru av 1.02 (i.e. Pru av 1.0201, Pru av 1.0202 and Pru av 1.0203). However, this primer set did not amplify Pru av 1.0101. All tested Pru av 1 sequences were amplified simultaneously using primer set 3. Finally, primer set 4 specifically detected Pru av 3.

**Conclusions**: Four novel real-time PCR primer sets have been designed which can be used in RT-qPCR experiments to detect Pru av 3 and Pru av 1 sequences. In the future, this could lead to the identification of natural hypoallergenic cherry cultivars.

**Keywords**: Cherry allergy; Pru Av 1; Pru Av 3

#### P20 Specificity of anti-Pru av 1 antibodies for the detection of Pru av 1 isoallergens

##### Martie C. M. Verschuren^1^, Shanna Bastiaan-Net^2^, Nikki M.E. Van Uden^1^, Karel Vandenberghe^1^, Kay Foetisch^3^, Stephan Scheurer^3^, Harry J. Wichers H.J.^2^, Theo H.M. Noij^1^

###### ^1^Avans University of Applied Science, School of Life Science and Environmental Technologies, Research group Analysis Techniques in Life Science, Breda, Netherlands, The; ^2^Wageningen UR Food & Biobased Research, Wageningen, Netherlands, The; ^3^Division of Allergology, Paul-Ehrlich Institut, Langen, Germany

**Correspondence**: Martie C. M. Verschuren

*Clinical and Translational Allergy* 2016, **6(Suppl 2)**:P20

**Background**: Fresh cherries can cause oral allergy symptoms in patients with birch pollinosis due to the cross reactivity of IgE to the birch pollen Bet v 1-homologous cherry protein Pru av 1. Previously, different Pru av 1 isoallergens and variants have been identified. These isoallergens showed diverging IgE-binding properties and therefore need to be taken into account when analyzing Pru av 1 protein expression levels in cherry cultivars. Therefore, we determined the specificity of anti-Pru av 1 monoclonal antibodies using Western blot analysis and ELISA.

**Materials and methods**: Coding sequences of Pru av 1.0101 and three Pru av 1.02 variants were cloned in a pET16b vector and transformed into E. coli strain BL21(DE3). Pru av 1 expression was induced and the presence of the four allergens in bacterial lysates was verified using LC–MS–MS analysis. Three anti-Pru av 1 monoclonal antibodies (F11, G4A, and G4D) and 1 polyclonal, the Pru av 1 cross reactive anti-Cor a1 antibody, were used in Western blot analysis of the bacterial lysates. Finally, specificity of a sandwich ELISA was determined using the F11 anti-Pru av 1 monoclonal antibody as catching antibody and the anti-Cor a 1 antibody as detecting antibody.

**Results**: Western blot analysis showed that the cross reactive anti-Cor a 1 antibody was capable of detecting all Pru av 1 proteins. The F11 monoclonal antibody strongly detected the Pru av 1.0101 protein but also had weak affinity for the other Pru av 1.02 variants. Monoclonal antibodies G4A and G4D were specific for isoallergen Pru av 1.0101. Sandwich ELISA using antibody F11 in combination with anti-Cor a 1 antibody easily identified the Pru av 1.0101 isoallergen. Pru av 1.0201 was only detected at high concentrations while the other Pru av 1.02 variants could not be identified using the ELISA.

**Conclusions**: The tested anti-Pru av 1 monoclonal antibodies can be used to specifically detect and quantify isoallergen Pru av 1.0101 in a sandwich ELISA.

**Keywords**: Cherry allergy; Pru Av 1

#### P21 Enhancing recombinant production yield of Bet v 1 through codon usage harmonization

##### Anargyros Roulias^1^, Maria Alejandra Parigiani^1^, Heidi Hofer^1^, Claudia Asam^1^, Christof Ebner^2^, Fátima Ferreira^1^

###### ^1^University of Salzburg, Department of Molecular Biology, Salzburg, Austria; ^2^Allergieambulatorium, Vienna, Austria

**Correspondence**: Anargyros Roulias

*Clinical and Translational Allergy* 2016, **6(Suppl 2)**:P21

**Background**: Improving production yield and elevating quality of recombinantly produced molecules is a high priority for the biotechnological as well as for the clinical sector. This issue has been tackled from numerous angles, one of them being codon usage bias. Crucial information concerning the overall protein synthesis procedure is encoded by the codon usage frequency; and hence even single synonymous codon mutations can significantly affect gene expression levels along with protein folding and function. Suboptimal codon usage bias can significantly limit heterologous protein expression. “Codon harmonization” is a strategy that effectively minimizes codon usage disparities between native and heterologous host by closely matching their respective codon usage frequencies. In this study we investigated the effects of codon harmonization in the recombinant production of the allergen Bet v 1.0101.

**Materials and methods**: Different batches of rBet v 1 and its harmonized version, Bet-Harm, were produced in parallel. Production yields were quantified by SDS-PAGE densitometry. All batches were physicochemically analyzed by mass spectrometry, amino acid analysis, circular dichroism, dynamic light scattering, Fourier transform infrared spectroscopy and ANS-binding assays. Immunological properties of the rBet v 1 and Bet-Harm batches were compared by endolysosomal degradation assays, ELISA and mediator release assays.

**Results**: A significant increase in protein yield and solubility was observed for Bet-Harm compared to rBet v 1. Besides, the two proteins displayed no alterations in their secondary structure elements, their behavior in solution and their ligand-binding ability. Furthermore, no differences in the proteolytic susceptibility, IgE-binding and IgE cross-linking ability of rBet v 1 and Bet-Harm were observed.

**Conclusions**: Codon harmonization is an effective approach towards increasing protein expression levels and should be considered as a potent strategy for overcoming protein production problems.

**Keywords**: Betv1; Recombinant; Codon harmonization; Expression levels

#### P22 Structural and dynamic insights into the world of PR-10 allergens

##### Linda Ahammer, Sarina Grutsch, Martin Tollinger

###### Institute of Organic Chemistry, Innsbruck, Austria

**Correspondence**: Linda Ahammer

*Clinical and Translational Allergy* 2016, **6(Suppl 2)**:P22

**Background**: Pathogenesis-related (PR) proteins are encoded by different genes that are induced to defend the plants against infections with fungi, bacteria or viruses. The exact function of the PR proteins of class 10 is still unclear. Most of the identified plant allergens can be grouped into the PR-10 protein family. A prominent member of this protein family is Bet v 1 (*Betula verrucosa*), the birch pollen allergen. Bet v 1 proteins represent one of the best-characterized group of model allergens in immunology. These proteins are one of the main causes of allergic reactions worldwide, with an estimated 100 million people affected. More than 70 % of birch pollen allergic patients are also reacting allergic to apple. Mal d 1 (*Malus domestica*) is the main allergen found in apples. The cross-reaction between Bet v 1 and Mal d 1 may result from a high degree of amino acid sequence identity (55–68 %) and the structural similarity of these two proteins.

**Materials and methods**: The goal of our study is to characterize and compare the structures, the stabilities and the flexibilities of allergenic PR-10 proteins from various sources (i.e. birch pollen and apple) by different NMR spectroscopic techniques.

**Results**: We rationalize the different allergenic and immunological properties of Bet v 1 and Mal d 1 and obtain structural insight into the cross-reactivity of these two allergens. Our data provide a comprehensive picture of the interplay between structure, dynamics and function of different allergens from the PR-10 family.

**Conclusions**: Among the factors that can contribute to the allergenic potential of proteins, we hypothesize that intrinsic dynamic and structural processes are not only critical but even essential for their immunologic properties.

**Keywords**: Bet V 1; Mal D 1; NMR spectroscopy

### Poster Session 3: Allergen molecules: identification, characterization, structure and function

#### P23 Purification of polcalcin from different pollen allergenic sources by antibody-affinity chromatography

##### Raquel Moya, Mª Angeles López-Matas, Raquel Reyes, Jerónimo Carnés

###### Laboratorios Leti S.L.U., Madrid, Spain

**Correspondence**: Raquel Moya

*Clinical and Translational Allergy* 2016, **6(Suppl 2)**:P23

**Background**: Polcalcins are small calcium-binding pollen proteins (around 9 kDa). They have been identified in pollen from diverse plant families where they are considered minor allergens. Due to their sequence homology and conserved structure, they show a high cross-reactivity. Polcalcins are not usually as much represented as other allergens in pollen extracts, for that reason their availability as purified proteins would represent an useful tool for diagnosis and/or immunotherapy purposes in allergic individuals.

The objective of this study was to purify the native polcalcin from different pollen allergenic sources by developing a purification method based on affinity chromatography.

**Materials and methods**: RNA from *Chenopodium album* pollen was obtained and used in RT-PCR with specific primers to obtain the polcalcin (Che a 3) cDNA. This cDNA was cloned in pQE31 vector and the protein expressed in *Escherichia coli.* The recombinant protein was purified by affinity chromatography and size exclusion filtration, characterized by immunoblot and sequencing and used to produce rabbit polyclonal antibodies. These antibodies anti-rChe a 3 were also purified and used to identify polcalcin in different pollen extracts by direct ELISA. An anti-rChe a 3 antibody-sepharose column was packed for the purification of polcalcin from *C. album* and *Olea europaea* extracts.

**Results**: The RT-PCR allowed the obtaining of a 261 pb cDNA which sequence corresponded to Che a 3. A highly purified 10 kDa-protein was obtained after its expression and purification in two steps and its identity confirmed as Che a 3. Polcalcin was detected by ELISA in eight pollen extracts including *Cynodon dactylon, C. album, Phleum pratense, O. europaea, Alnus glutinosa, Anthoxantum odoratum*, *Parietaria judaica*, and *Betula alba*. Extracts from *O. europaea* and *C. album* were selected for the purification of native polcalcin. Purified Che a 3 and Ole e 3 were obtained with a high degree of purity (>95 %). Ole e 3 was identified by mass-spectrometry.

**Conclusions**: Affinity chromatography based on specific antibodies is an efficient **Materials and methods** for the purification of native polcalcin from different allergenic sources.

#### P24 Variations of wheat allergens in cultivars measured through a targeted quantitative mass spectrometry approach

##### Colette Larré, Hélène Rogniaux, Roberta Lupi, Sandra Denery-Papini

###### INRA UR1268 Biopolymers Interactions Assemblies, Nantes, France

**Correspondence**: Colette Larré

*Clinical and Translational Allergy* 2016, **6(Suppl 2)**:P24

**Background**: Wheat is an important part of the daily diet of millions of people; however, this staple food is also responsible for food allergies. Food allergy has become a major health issue in developed countries, therefore there is an urgent need to develop analytical methods able to detect and quantify with a good sensitivity and reliability some specific allergens in complex food matrices.

Wheat genus presents species with different ploidy levels, among which: diploid species with genome A (*T.* *monococcum*, einkorn); tetraploid with genomes A and B (durum wheat, Emmer); and hexaploid with genomes A, B and D (spelt, bread wheat). The bread wheat currently cultivated (*T.* *aestivum*) is hexaploid (genome ABD). It originated through spontaneous hybridizations between A, B and D genome of ancestral species and has been widely bred. In the case of prolamins, there is experimental evidence for a natural variation in the degree of biological activity between cereal genus (oat, rye, barley and wheat) as well as in the genus Triticum.

*T. monococcum*, is an ancient species which has received renewed attention in the context of low impact and sustainable agriculture and because of its potential nutritional qualities. In addition, it was shown to exhibit a lower IgE-binding capacity compared to hexaploid species. We present a targeted MS/MS approach to compare the relative abundance of the major recognized wheat allergens in the salt-soluble (albumin/globulin) fraction of wheat grains.

Twelve allergens were quantified in seven wheat varieties, selected from three *Triticum* species: species: *T. aestivum* (bread wheat), *T. durum* (durum wheat), and *T. monococcum*.

**Materials and methods**: The allergens were monitored from one or two proteotypic peptides and their relative abundance was deduced from the intensity of one fragment measured in MS/MS.

**Results**: Whereas the abundance of some of the targeted allergens was quite stable across the genotypes, others like alpha-amylase inhibitors showed clear differences according to the wheat species, in accordance with the results of earlier functional studies.

**Conclusions**: This study enriches the scarce knowledge available on allergens content in wheat genotypes, and brings new perspectives for food safety and plant breeding.

**Keywords**: Allergens; Genotypes; MRM MS; Quantification; Wheat

#### P25 Art v 1, Amb a 4 and Par h 1 defensin-like proteins share similar structural features but distinct immunological and allergenic properties

##### Isabel Maria Pablos^1^, Stephanie Eichhorn^1^, Yoan Machado^1^, Peter Briza^1^, Christof Ebner^2^, Jung-Won Park^3^, Alain Didierlaurent^4^, Naveen Arora^5^, Stefan Vieths^6^, Gabriele Gadermaier^1^, Fatima Ferreira^1^

###### ^1^University of Salzburg, Salzburg, Austria; ^2^Allergy Clinic Reumannplatz, Vienna, Austria; ^3^Yonsei University College of Medicine, Seoul, Korea; ^4^Stallergenes S.A, Antony, France; ^5^CSIR-Institute of Genomic and Integrative Biology, Delhi, India; ^6^Paul-Ehrlich-Institut, Langen, Germany

**Correspondence**: Isabel Maria Pablos

*Clinical and Translational Allergy* 2016, **6(Suppl 2)**:P25

**Background**: Defensin-like proteins such as Art v 1, the major allergen from mugwort (*Artemisia vulgaris*), Amb a 4 from ragweed (*Ambrosia artemisiifolia*) and Par h 1 from feverfew (*Parthenium hysterophorus*) are important triggers of allergy. They consist of an N-terminal globular domain and a C-terminal proline-rich extended tail. To investigate the structural and immunological features of these proteins, Art v 1, Amb a 4 and Par h 1 were expressed in *E. coli* and purified to homogeneity.

**Materials and methods**: Proteins were characterized using mass spectrometry, dynamic light scattering, circular dichroism and Fourier transform infrared spectroscopy. For immunological studies the proteins were subjected to endolysosomal degradation and *in vitro* antigen uptake with BMDCs. IgE recognition and cross-inhibition experiments were assessed by ELISA using patients’ sera from Austria (n = 40), Canada (n = 38) and Korea (n = 27). The allergenic activity was studied by mediator release assay.

**Results**: The identity of purified proteins was confirmed by mass spectrometry. The three proteins were monomeric as determined by dynamic light scattering. Fourier transform infrared spectroscopy revealed comparable content of α-helices and β-sheets, indicating similar foldings for the three allergens. Despite of their secondary structural similarity, the uptake kinetics were markedly different, with Amb a 4 being more efficiently internalized by BMDCs, followed by Art v 1 and Par h 1. The defensin domain of Art v 1 was more stable to endolysosomal proteolysis compare to Amb a 4 and Par h 1, pointing to possible differences in their immunogenicity. All three allergens triggered IgE-mediated basophil degranulation ranging from 15 to 40 %, demonstrating their allergenicity. The IgE reactivity of Art v 1 was significantly higher in Austrian and Korean cohorts compared with Amb a 4 and Par h 1, while in the Canadian cohort all allergens showed similar reactivity. Additionally, in cross-reactivity studies, Amb a 4 and Par h 1 showed a higher cross-reactivity between them compared to Art v 1, which in some cases was unable to inhibit the response against Amb a 4 or Par h 1. This data suggests the presence of shared-common IgE epitopes while others are allergen-specific.

**Conclusions**: Art v 1, Amb a 4 and Par h 1 have similar structural features but show different immunological behavior *in vitro*. The three allergens share common IgE epitopes while they display different IgE sensitization profiles in patients’cohorts.

**Keywords**: Weed pollen allergens; Cross-reactivity; Allergenicity

#### P26 Homogeneity or diversity of IgE-binding proteins in wheat dependant exercise induced anaphylaxis ?

##### Sandra Denery-Papini^1^, Charlene Tanaka^2^, Florence Pineau^1^, Roberta Lupi^1^, Martine Drouet^3^, Etienne Beaudouin^4^, Martine Morisset^5^, Susan Altenbach^2^

###### ^1^UR1268 BIA, INRA, Nantes, France; ^2^USDA-ARS Western Regional Research Center, Albany, United States; ^3^Unité d’Allergologie Générale et de Pneumologie, CHU d’Angers, Angers, France ; ^4^Service d’Allergologie, CH Epinal, Epinal, France ; ^5^Immunologie-Allergologie, CH de Luxembourg, Luxembourg, Luxembourg

**Correspondence**: Sandra Denery-Papini

*Clinical and Translational Allergy* 2016, **6(Suppl 2)**:P26

**Background**: Wheat is involved in 7.5 % of the severe food anaphylaxis according to the 2014 statistics of the French Allergo-vigilance Network. Exercise Induced Anaphylaxis is the most common form of wheat food allergy seen in adult patients and triggered by wheat intake and exercise in the following hours. Omega-5 gliadins (Tri a 19) have been documented for their interest in WDEIA diagnosis; however, in a number of cases, involvement of other proteins such as high MW and low MW glutenin subunits (HMW-GS and LMW-GS) and Lipid Transfer Protein (LTP) has been suggested.

Wheat is a complex mixture of proteins; within a single cultivar, more than 100 constituents can be observed, among which, gliadins and glutenins present a high polymorphism and large sequence homologies. Two-dimensional immunoblot analysis after extraction of total proteins is a powerful technique to observe the diversity of IgE-binding in wheat flour, preserving the natural polymorphism, while eliminating cross-contamination issues.

**Materials and methods**: Sera obtained from adult patients diagnosed with WDEIA were analyzed by immunoblot after two-dimensional separation of total proteins from the US wheat cultivar Butte 86. The availability of a proteomic map from this cultivar in which most of the major flour proteins were identified by tandem mass spectrometry makes it possible to determine the specific flour proteins that react with patients IgE. Comparison was carried out with ELISA results.

**Results**: As expected, most of the sera (70 %) reacted strongly to omega-5 gliadins in ELISA and detected multiple corresponding 2-DE spots. This reactivity was often accompanied in immunoblot by weak reactivity to certain LMW-GS and alpha-gliadins which may correspond to cross-reactions, but that did not always correlate with ELISA results. The majority of patients with no IgE reactivity to omega-5 gliadins reacted to wheat LTP (about 20 % of cases). A few sera exhibited very different and diverse profiles: strong reactivity to all gliadins and LMW-GS, predominant binding to alpha-gliadins, specific binding to HMW-GS or specific binding to salt-soluble proteins including alpha-amylase inhibitors and peroxidases.

**Conclusions**: These data suggest that multiple proteins may be involved in WDEIA, even though some of these reactions occur as cross-reactions to the major allergens omega-5 gliadins. Wheat lacking omega-5 gliadins would result in decreased allergenic potential but would not be suitable for individuals already diagnosed with WDEIA.

**Keywords**: Wheat allergens; EIA; 2D immunoblots

#### P27 Deciphering the role of disulfide bonds and of repetitive epitopes in immunoglobulin E binding to wheat gliadins

##### Sandra Denery-Papini^1^, Hamza Mameri^1^, Chantal Brossard^1^, Roberta Lupi^2^, Florence Pineau^1^, Jean Charles Gaudin^1^, Denise Anne Moneret-Vautrin^3^, Etienne Beaudouin^3^, Evelyne Paty^4^, Martine Drouet^5^, Olivier Tranquet^1^, Colette Larré^1^

###### ^1^UR1268 BIA, INRA, Nantes, France; ^2^UR1268 BIA, Nantes, France; ^3^CH Epinal, Epinal, France; ^4^Hopital Necker, Paris, France; ^5^CHU Angers, Angers, France

**Correspondence**: Sandra Denery-Papini

*Clinical and Translational Allergy* 2016, **6(Suppl 2)**:P27

**Background**: The prolamin superfamily comprises the largest number of allergenic plant proteins. The members possess a conserved skeleton of 8 cysteine residues that are connected by intramolecular disulfide bonds. The sulfur-rich prolamins of wheat, alpha-gliadins, gamma-gliadins and low molecular weight glutenin subunits differ by their organization into two structural domains: the C-terminal (Ct-) domain, which include the cysteine skeleton, and the N-terminal (Nt-) domain, which contains repeating motifs. Wheat gliadins are frequently involved in food allergy to wheat and occasionally involved in baker’s asthma. The present study aimed to decipher the molecular basis of α- and γ-gliadin allergenicity.

**Materials and methods**: Different forms (natural/recombinant; native/reduced-alkylated) and fragments (Nt- and Ct-) of α- and γ-gliadins were produced to study the contribution of their structural domains and disulfide bonds to IgE binding. Their secondary structure was determined using synchrotron radiation circular dichroism (SRCD); sera from patients with food allergies to wheat or baker’s asthma were used to analyze IgE binding and triggering potential on RBL cells.

**Results**: The secondary structures of natural and recombinant proteins were slightly different. Compared with natural gliadins, recombinant proteins retained IgE binding but with reduced reactivity. When the proteins were reduced/alkylated, no significant effect was observed on the secondary structures of α- and γ-gliadin while significant decrease of IgE binding for both natural and recombinant gliadins was observed. Thus, reduction may have an indirect but drastic effect on the global folding of the proteins. Evaluation of the triggering potential of α-gliadin led to similar conclusions. IgE binding to peptides and recombinant domains of gliadins indicated the presence of epitopic regions in both domains.

**Conclusions**: In Conclusions, the α- and γ-gliadins in their native form should be preferentially used for IgE reactivity detection. As for other allergens of the prolamin superfamily, formation of disulfide bonds appears to be of importance for IgE binding to a and g-gliadins. Disulfide bonds in the Ct-domain most likely play a role in generating conformational epitopes within this domain or which are formed by a combination of residues from both domains.

**Keywords**: Food allergy; Wheat allergens; Epitopes

**Reference**

1. Mameri et al., J Agric Food Chem. 2015, 63:6546–54

#### P28 Assessment of the allergenicity of soluble fractions from bread and durum wheats genotypes

##### Roberta Lupi^1^, Stefania Masci^2^, Olivier Tranquet^1^, Denise-Anne Moneret-Vautrin^3^, Sandra Denery-Papini^1^, Colette Larré^1^

###### ^1^INRA UR1268 BIA, Nantes, France; ^2^University of Tuscia, Viterbo, Italy; ^3^Faculté de Médecine de Nancy, Service d’Allergologie, Epinal, France

**Correspondence**: Roberta Lupi

*Clinical and Translational Allergy* 2016, **6(Suppl 2)**:P28

The published version of this abstract can be found at [1].

**Reference**

1. Lupi R, Masci S, Rogniaux H, Tranquet O, Brossard C, Lafiandra D, Moneret-Vautrin DA, Denery-Papini S, Larre C. Assessment of the allergenicity of soluble fractions from GM and commercial genotypes of wheats. J. Cereal Sci. 2014. 60(1):179–86.

#### P29 Isolation and characterization of Ara h 12 and Ara h 13: defensins, a novel class of peanut allergens

##### Skadi Kull^1^, Arnd Petersen^1^, Marisa Böttger^1^, Sandra Rennert^1^, Wolf-Meinhard Becker^1^, Susanne Krause^1^, Martin Ernst^2^, Thomas Gutsmann^3^, Johann Bauer^4^, Buko Lindner^5^, Uta Jappe^6^

###### ^1^Division of Clinical and Molecular Allergology, Research Center Borstel, Airway Research Center North (ARCN), Member of the German Center for Lung Research (DZL), Borstel, Germany; ^2^Division of Immune Cell Analytics, Research Center Borstel, Borstel, Germany; ^3^Division of Biophysics, Research Center Borstel, Borstel, Germany; ^4^Chair of Animal Hygiene, Technische Universität München, Freising-Weihenstephan, Germany; ^5^Division of Bioanalytical Chemistry, Research Center Borstel, Borstel, Germany; ^6^Division of Clinical and Molecular Allergology, Research Center Borstel, Airway Research Center North (ARCN), Member of the German Center for Lung Research (DZL), Interdisciplinary Allergy Outpatient Clinic, MK III, University of Lübeck, Borstel/Lübeck, Germany

**Correspondence**: Skadi Kull

*Clinical and Translational Allergy* 2016, **6(Suppl 2)**:P29

**Background**: Peanut allergy is one of the most severe food allergies worldwide. For estimating the allergenic risk of peanut allergens and for improving diagnostic tests, allergens that lead to severe clinical reactions need to be identified. There is evidence that lipophilic allergens are associated with severe clinical symptoms, but many of them are not included in diagnostic test systems so far or are still unknown due to the complexity of the peanut matrix. Therefore, the aim of this study was to detect, isolate and characterize novel peanut allergens with lipophilic properties. Based on the observation that food processing, for example roasting, influences the allergenicity of molecules, we also investigated the impact of roasting on the IgE-binding of the novel peanut allergens.

**Materials and methods**: Using lipophilic extraction methods and subsequent chromatographic separation techniques, we were able to isolate low molecular weight compounds in the range of 10–12 kDa under non-reducing conditions from roasted and unroasted peanuts respectively. These proteins were further characterized by immunoblot analysis with sera of peanut-allergic patients. Allergic but non-peanut allergic patients and non-allergic individuals were used as controls. Additionally, characterization of the allergenicity was performed by basophil activation test and further functional properties of the molecules were analysed by antimicrobial activity assays.

**Results**: The low molecular weight compounds were identified as peanut defensins. The World Health Organization/International Union of Immunological Societies (WHO/IUIS) accepted them as two novel allergens (Ara h 12, and Ara h 13). Peanut allergic patients show a higher IgE reactivity against roasted peanut defensin compared to unroasted peanut defensin. Furthermore, we were able to show that defensins can activate basophils of peanut-allergic patients and have an inhibitory effect on mould strains, like *Cladosporium* and *Alternaria*, but not on the so far tested bacteria strains.

**Conclusions**: Our data suggests that the IgE reactivity correlates with severe clinical symptoms. For substantiating this observation and the impact of lipophilic allergens for component resolved diagnostics, future studies with higher numbers of peanut-allergic patients are needed.

**Keywords**: Peanut; Ara H 12/Ara H 13; Food allergy; Lipophilic extraction; Plant defensins

#### P30 Allergenicity attributes of different peanut market types

##### Stef Koppelman^1^, Shyamali Jayasena^1^, Dion Luykx^2^, Erik Schepens^2^, Danijela Apostolovic^3^, Govardus De Jong^4^, Tom Isleib^5^, Julie Nordlee^1^, Joe Baumert^1^, Steve Taylor^1^, Soheila Maleki^6^

###### ^1^University of Nebraska, Lincoln, Ne, United States; ^2^HAL Allergy, Leiden, Netherlands, The; ^3^University of Belgrade, Belgrade, Serbia; ^4^TNO, Zeist, Netherlands, The; ^5^North Carolina State University, Raleigh, Nc, United States; ^6^USDA ARS, New Orleans, La, United States

**Correspondence**: Stef Koppelman

*Clinical and Translational Allergy* 2016, **6(Suppl 2)**:P30

The published version of this abstract can be found at [1].

**Reference**

1. Koppelman SJ, Jayasena S, Lukyx D, Schepens E, Aposotolvic D, de Jong GAH, Isleib TG, Nordlee J, Baumert J, Taylor SL, Cheng H, Maleki S. Allergenicity attributes of different peanut marker types. Food Chem. Toxicol. 2016; 91: 82–90.

#### P31 The impact of peanut lipids on Ara h 1-induced immune responses in monocytes-derived dendritic cells

##### Chiara Palladino^1^, Barbara Gepp^1^, Sofía Sirvent^2^, Alba Angelina^2^, Merima Bublin^1^, Christian Radauer^1^, Nina Lengger^1^, Thomas Eiwegger^3^, Oscar Palomares^2^, Heimo Breiteneder^1^

###### ^1^Department of Pathophysiology and Allergy Research, Medical University of Vienna, Vienna, Austria; ^2^Department of Biochemistry and Molecular Biology, School of Chemistry, Complutense University of Madrid, Madrid, Spain; ^3^Division of Clinical Immunology and Allergy, The Hospital for Sick Children, Toronto, Canada

**Correspondence**: Chiara Palladino

*Clinical and Translational Allergy* 2016, **6(Suppl 2)**:P31

**Background**: Although the uptake of the major peanut allergen Ara h 1 by dendritic cells (DCs) has been described, it is still not clear whether the allergen is able to sensitize by itself or whether there are other molecules involved. Evidence of the role of small molecules in the allergic sensitization, such as lipids, directly bound as ligands by the allergen or present in the allergen source, is emerging. Peanuts contain a significant amount of lipids. Therefore, we aimed to assess whether peanut lipids can bind to Ara h 1, and whether they can modulate the allergen-induced response in monocyte-derived dendritic cells (MoDCs).

**Materials and methods**: Ara h 1 was purified from commercially available roasted peanuts by standard chromatographic methods. Extraction of total lipids from peanut was performed using the chloroform/methanol method. The lipid concentration was determined by the sulfo-phospho-vanillin method. Lipid binding to Ara h 1 was evaluated by 1-anilinonaphthalene-8-sulfonic acid (ANS) displacement assay. ANS was used at 5 µM, and peanut lipids were added to Ara h 1 at 5, 50, and 100 µM. Fluorescence was measured at 484 nm. Monocytes were purified from PBMCs of 10 non-peanut allergic individuals and differentiated for 6 days with IL-4 and GM-CSF. The resulting MoDCs were treated with 10 µg/ml peanut lipids alone or in combination with 10 µg/ml Ara h 1. TNF-α levels in the cell culture supernatant were measured by ELISA.

**Results**: Ara h 1 incubated with peanut lipids at the highest concentration, showed a reduction of ANS fluorescence by 30 % compared with Ara h 1 incubated with ANS alone. In MoDCs from 5 of 10 non-peanut allergic individuals Ara h 1 increased the TNF-α production. Interestingly, peanut lipids reduced TNF-a levels in response to Ara h 1 stimulation by 65 %. In the other 5 donors, Ara h 1 did not elicit any TNF-α response, but peanut lipids in combination with Ara h 1 showed a non-significant increase of TNF-α levels.

**Conclusions**: We observed that lipids extracted from peanut were able to bind to Ara h 1. Furthermore, peanut lipids diminish the Ara h 1-induced TNF-α response in MoDCs of non-allergic individuals. These data indicate that peanut lipids do play a role in the modulation of the immune response to Ara h 1.

Supported by the Austrian Science Fund Doctoral Program MCCA W 1248-B13.

**Keywords**: Peanut allergy; Peanut lipids; MoDCs; Sensitization

#### P32 Compared allergenicity of native and thermally aggregated ovalbumin as large agglomerated particles

##### Mathilde Claude, Roberta Lupi, Grégory Bouchaud, Marie Bodinier, Chantal Brossard, Sandra Denery-Papini

###### INRA UR 1268 BIA (Biopolymers, Interactions, Assemblies), Nantes, France

**Correspondence**: Mathilde Claude

*Clinical and Translational Allergy* 2016, **6(Suppl 2)**:P32

**Background**: Several studies reported data, sometimes contradictory, about the effect of heat-treatment of egg on allergy using mice or sera form egg allergic patients; but the possible impact of aggregation was not considered. Aggregation is an irreversible modification of proteins by formation of new intermolecular bonds. Aggregates of various morphologies may be generated depending on physico-chemical conditions. Ovalbumin, a major allergen of egg white, is prone to aggregate upon thermal processing. This study compares allergenicity of native and aggregated ovalbumin as large agglomerated particles on parameters from both phases of allergic reaction: sensitization and elicitation.

**Materials and methods**: An ovalbumin solution (pH5–0.8 M) was extensively heated (80 °C for 6 h) to generate large agglomerated aggregates. A murine model of allergy was used to evaluate influence of ovalbumin structure on Ig production upon sensitization by intraperitoneal route. Using sera from mice sensitized to native or aggregated ovalbumin, Ig-binding and degranulation capacities of native and aggregated ovalbumin were measured by ELISA and with RBL cells.

**Results**: We showed that heat-aggregation of ovalbumin as large particles enhanced IgG production and promoted a shift toward IgG_2a_ production in mice (pro-Th_1_ profile) compared to native ovalbumin (pro-Th_2_ profile). Moreover, the IgG repertory differed i.e. aggregated ovalbumin generated antibodies able to bind both native and aggregated ovalbumin. Large agglomerated aggregates displayed lower IgE-binding and RBL activation capacities.

**Conclusions**: This work illustrates links between structure of ovalbumin and allergenicity potential on parameters from both sensitization and elicitation phases of the allergic reaction. It would appear that large agglomerated aggregates of OVA increased sensitization by producing more IgG and decreased elicitation.

**Keywords**: Egg allergy; Food processing; Aggregation; Basophil activation

#### P33 Simulation of the gastrointestinal digestion of the hazelnut allergens Cor a 9 and Cor a 11 by an in-vitro model and characterisation of peptidic products including epitopes by HPLC-MS/MS

##### Robin Korte, Julia Bräcker, Jens Brockmeyer

###### University of Münster, Münster, Germany

**Correspondence**: Robin Korte

*Clinical and Translational Allergy* 2016, **6(Suppl 2)**:P33

**Background**: Allergic reactions against certain foods are triggered by epitopic regions in the allergen’s amino acid sequence that show the ability to bind IgE. For an allergic reaction to occur, the passage of these epitopes through the intestinal mucosa in an immunologically intact form is required (not considering oral allergies that tend to show far less severe symptoms). Epitopes have been identified for many major tree nut allergens known to be able to trigger anaphylaxis. Still very little is known about their molecular fate during the gastro-intestinal passage and the IgE-binding potential of the digestive products.

**Materials and methods**: A standardised multistage in-vitro model (Minekus et al. 2014), simulating the three phases of human digestion (oral, gastral, intestinal), was applied to ground hazelnut kernels. The time-dependent course of the in-vitro digestion processes was analysed by SDS-PAGE. A software assisted HPLC-MS/MS proteomics approach on a high resolution orbitrap mass spectrometer was used to elucidate the structure of peptidic degradation products and assess their relative concentrations over time.

**Results**: Degradation was observed for both major allergens from hazelnut kernels, Cor a 9 and Cor a 11, in the gastral and intestinal phases accompanied by the formation of low-molecular products. While Cor a 11 showed higher stability against digestion both molecules were largely degraded after 120 min in the gastral and 10 min in the intestinal phase. The molecular structures of several peptidic products were identified by mass spectrometry. Time dependent formation and degradation kinetics of these peptides indicate that the gastrointestinal digestion of the intact allergens follows a multi-stage mechanism based on consecutive enzymatic activity resulting in a broad variety of peptidic structures. While the concentration of the intact allergens decreased rapidly during the digestion processes, several of the peptidic degradation products overlap with previously identified linear epitopes.

**Conclusions**: Human digestion significantly affects the molecular structure of allergens by gastral and intestinal degradation processes. The major hazelnut allergens Cor a 9 and Cor a 11 are shown to be largely degraded before they reach the intestinal lumina whereas particular parts of the protein sequence resist digestion. The stability of specific linear epitope against gastrointestinal degradation therefore appears to play a crucial role for their allergenic potential.

**Keywords**: Hazelnut allergy; In-vitro digestion; High resolution mass spectrometry

#### P34 Analysis of distribution of rice allergens in brown rice grain and allergenicity of the products containing rice bran

##### Rie Satoh^1^, Reiko Teshima^2^

###### ^1^National Food Research Institute, National Agriculture and Food Research Organization, Tsukuba, Ibaraki, Japan; ^2^National Institute of Health Sciences, Setagaya, Tokyo, Japan

**Correspondence**: Rie Satoh

*Clinical and Translational Allergy* 2016, **6(Suppl 2)**:P34

**Background**: Rice allergy is a problem in both, Asian and Western countries. Several rice proteins have already been identified as causative agents of rice allergy. Since the first case of occupational contact urticaria was reported in a housewife and was due to the handling of rice bran in the form of rice bran pickles, it is necessary to understand the allergenicity of whole brown rice, not only polished rice but also rice bran, and to consider the sensitization by percutaneous exposure. In this study, we analysed the distribution of rice allergens in brown rice grain and the allergenicity in health food and cosmetics containing rice bran.

**Materials and methods**: Brown rice (*Oryza sativa* L. cv koshihikari), polished rice, and rice bran were powdered and suspended in 1 M NaCl containing protease inhibitors or Laemmli sample buffer and proteins were extracted. For the health foods and cosmetics containing rice bran, samples were suspended in phosphate-buffered saline (PBS) and proteins were extracted. The extracted protein solutions were then centrifuged and filtered. The proteins were separated by sodium dodecyl sulfate-polyacrylamide gel electrophoresis (SDS–PAGE) under reducing conditions, and analysed using western blotting with rabbit polyclonal antibody specific to two rice allergens, 19 kDa globulin and 52 kDa globulin.

**Results**: The results of western blot analysis revealed that the 19 kDa globulin was hardly detected in the rice bran but was detected in the brown rice and the polished rice. On the other hand, 52 kDa globulin was detected in the rice bran and the brown rice, and was hardly detected in the polished rice. Regarding the proteins from the health foods and cosmetics, some of them showed clear protein bands and the others hardly showed the bands. Furthermore, 19 and/or 52 kDa globulins were detected in some products because the western blot analysis used the rice allergen specific antibody.

**Conclusions**: 19 and 52 kDa globulins are localized mainly in the inner and outer parts of the rice grains, respectively. The finding that some health foods and cosmetics with rice bran contain 19 and 52 kDa globulins indicates that the patients with rice allergy need to be careful about using these products.

**Keywords**: Rice; Rice bran; Globulin; Health foods; Cosmetics

### Poster Session 4: Molecular approaches in AIT

#### P35 Production of a recombinant hypoallergenic variant of the major peanut allergen Ara h 2 for allergen-specific immunotherapy

##### Angelika Tscheppe^1^, Dieter Palmberger^2^, Merima Bublin^1^, Christian Radauer^1^, Chiara Palladino^1^, Barbara Gepp^1^, Nina Lengger^1^, Reingard Grabherr^2^, Heimo Breiteneder^1^

###### ^1^Department of Pathophysiology and Allergy Research, Medical University of Vienna, Vienna, Austria; ^2^Department of Biotechnology, University of Natural Resources and Life Sciences, Vienna, Austria

**Correspondence**: Angelika Tscheppe

*Clinical and Translational Allergy* 2016, **6(Suppl 2)**:P35

**Background**: Peanut allergy is one of the most dangerous food allergies. Ara h 2 is a major peanut allergen. At present, peanut allergen-specific immunotherapy is not available for clinical use. We aimed to produce a hypoallergenic mt (mutant) Ara h 2.

**Materials and methods**: An *in silico* designed mtAra h 2 in which IgE-binding surface exposed loops were removed and wt (wild-type) Ara h 2 with a hexahistidyl-tag were expressed in *Trichoplusia ni* BTI-TN5B1-4 ‘‘HighFive’’ cells. Following purification from the supernatants, mtAra h 2 and wtAra h 2 protein expression was verified by SDS-PAGE and Western blotting. Physicochemical characteristics were determined by mass spectrometry, CD spectrometry and N-terminal sequencing. IgE-binding to purified n (natural) Ara h 2, wtAra h 2 and mtAra h 2 was tested by direct ELISA, Western blotting and inhibition ELISA.

**Results**: Mass spectrometry analysis confirmed the absence of post-translational modifications for the main fraction of wtAra h 2. The folding and the N-terminal amino acid sequence of wtAra h 2 corresponded to the natural protein. For mtAra h 2, mass spectrometry analysis revealed a mass corresponding to the predicted size. The folding as well as the N-terminal amino acid sequence were identical to the natural protein with removed IgE-binding loops. Immunoblots of Ara h 2-sensitized patients showed lower IgE-binding to mtAra h 2 than to the wild-type and the natural protein. In direct ELISA, five peanut allergic patients’ sera revealed a 20–50 % reduced IgE-binding to mtAra h 2 compared to wtAra h 2. Inhibition ELISAs showed significantly reduced IgE-binding of mtAra h 2 compared with nAra h 2.

**Conclusions**: The obtained results indicate that mtAra h 2 is a promising template for designing the next generation of hypoallergenic mutants.

Supported by the Austrian Science Fund doctoral program W1248-B13 (Doctoral Program Molecular, Cellular and Clinical Allergology, MCCA).

**Keywords**: Allergen-specific immunotherapy; Ara H 2; Hypoallergenic mutant; Baculovirus insect cell system

#### P36 Mutagenesis of amino acids critical for calcium-binding leads to the generation of a hypoallergenic Phl p 7 variant

##### Marianne Raith^1^, Linda Sonnleitner^2^, Doris Zach^1^, Konrad Woroszylo^1^, Margit Focke-Tejkl^3^, Herbert Wank^1^, Thorsten Graf^4^, Annette Kuehn^4^, Ines Swoboda^1^

###### ^1^Molecular Biotechnology Section, University of Applied Sciences, FH Campus Wien, Campus Vienna Biocenter, Vienna, Austria; ^2^Department of Biomedical Analytics, University of Applied Sciences Wiener Neustadt, Wiener Neustadt, Austria; ^3^Division of Immunopathology, Department of Pathophysiology and Allergy Research, Center for Pathophysiology, Infectiology and Immunology, Medical University of Vienna, Vienna, Austria; ^4^Department of Infection and Immunity, Luxembourg Institute of Health, Esch-Sur-Alzette, Luxembourg

**Correspondence**: Marianne Raith

*Clinical and Translational Allergy* 2016, **6(Suppl 2)**:P36

**Background**: Immunotherapy, the only causative treatment for type I allergies, may cause severe side effects. Therefore new strategies for safer forms of specific immunotherapy are needed. One strategy is to modify allergens in a way that they show reduced IgE-binding capacity and thus upon application have a lower risk of inducing severe side effects.

**Materials and methods**: We previously produced a hypoallergenic mutant of the major fish allergen, parvalbumin, by introduction of point mutations into the two calcium-binding sites. To see whether the same strategy can also be applied for other calcium-binding allergens, we introduced by site-directed mutagenesis the same mutations into the calcium-binding sites of Phl p 7, a polcalcin from timothy grass. The histidine-tagged wildtype and mutant variant of Phl p 7 were expressed in *Escherichia coli* and purified using affinity column chromatography.

**Results**: The successful expression of the wildtype and mutant proteins was shown in immunoblots using anti-histidine as well as anti-Phl p 7 antibodies. Immunoblots and dot blots with sera from grass pollen allergic individuals showed a strongly reduced IgE-binding capacity of the mutant as compared to the wildtype allergen. These results demonstrated that mutagenesis of specific amino acids involved in calcium-binding generated a hypoallergenic Phl p 7 molecule. Circular dichroism spectroscopy experiments performed in the presence and absence of calcium further showed that the Phl p 7 mutant had lost the calcium-binding capacity. To analyze whether the Phl p 7 mutant variant represents an immunogenic molecule, rabbits were immunized with the protein. ELISA analyses showed that the Phl p 7 mutant induced specific IgG antibodies, which recognized the mutant protein and also the Phl p 7 wildtype allergen.

**Conclusions**: By mutagenesis of specific amino acids involved in calcium-binding we thus generated a hypoallergenic Phl p 7 molecule which still has the potential to stimulate the immune system. We therefore suggest, that Phl p 7 mutant protein could be used for specific immunotherapy of patients sensitized to calcium-binding pollen allergens.

**Keywords**: Polcalcin; Immunotherapy; Calcium-binding sites

#### P37 Are birch pollen allergen immunotherapy induced blocking antibodies protective for cross-reactive allergens?

##### Claudia Asam^1^, Sara Huber^1^, Heidi Hofer^1^, Roland Lang^2^, Thomas Hawranek^2^, Fátima Ferreira^1^, Michael Wallner^1^

###### ^1^Department of Molecular Biology, University of Salzburg, Salzburg, Austria; ^2^Department of Dermatology, Paracelsus Private Medical University Salzburg, Salzburg, Austria

**Correspondence**: Claudia Asam

*Clinical and Translational Allergy* 2016, **6(Suppl 2)**:P37

**Background**: Pollen from Fagales trees, especially birch pollen, is the main cause of early seasonal allergy in the temperate climate zone of the Northern hemisphere. Birch pollen allergic patients frequently develop allergies also towards various fruits, vegetables and nuts belonging to the botanical families of Rosaceae, Apiaceae and Fabaceae, respectively. Common symptoms in birch allergic patients include the oral allergy syndrome against apple and hazelnut. Allergen immunotherapy (AIT) is currently the only strategy to effectively cure allergies and the success of the treatment is influenced by the development of specific blocking antibodies. The aim of this study was to investigate whether blocking antibodies induced by successful birch pollen AIT using conventional allergen extracts would inhibit IgE binding to Bet v 1 related pollen as well as food allergens.

**Materials and methods**: Therefore, a panel of 8 Fagales pollen allergens (Aln g 1.0101 from alder, Bet v 1.0101 from birch, Car b 1.0109 from hornbeam, Cas s 1.0101 from chestnut, Cor a 1.0104 from hazelnut, Fag s 1.0101 from beech, Ost c 1.0101 from hop-hornbeam and Que a 1.0301 from oak) as well as two related food allergens (Mal d 1.0108 from apple and Cor a 1.0401 from hazelnut) were recombinantly produced in *E. coli*, purified to homogeneity and analyzed physico-chemically. Serum samples from 5 different birch pollen allergic patients were collected before, after reaching maintenance dose and after 1-year after initiation of AIT with birch pollen extracts. Treatment efficacy was analyzed by nasal provocation tests. Facilitated antigen binding (FAB) assays were performed to determine the blocking activity of AIT induced IgG.

**Results**: All five patients included in the study showed an improvement of nasal provocation scores during AIT. Moreover, all patients developed Bet v 1 specific blocking antibodies. However, FAB assays revealed that not all donors developed blocking antibodies against the whole panel of Bet v 1 related allergens.

**Conclusions**: Therefore, we believe that successful birch pollen AIT cannot always ameliorate allergic symptoms towards related allergens emphasizing the need for improved treatment strategies.

**Keywords**: Allergy; Fagales pollen; Facilitated antigen binding assay

#### P38 High success of 58 subcutaneous immunotherapy for pets allergy in a polyallergic cohort of patients: a component resolved individually adapted treatment (CRIAT)

##### Fabienne Gay-Crosier

###### Private practice, Carouge, Switzerland

**Correspondence**: Fabienne Gay-Crosier

*Clinical and Translational Allergy* 2016, **6(Suppl 2)**:P38

**Background**: Allergy to pets is a public health problem [1–3]. Geneva is a multiple complex community [4] where the use of component resolved diagnostic (CRD) is unavoidable [5].

**Materials and methods**: Multiplex microarray Isac^®^ 112 was performed for 431 patients from 2012 to 2014; prevalence of pets allergens were recorded. Results of subcutaneous immunotherapy started between 2005 and 2010 on 58 patients allergic to pets were analysed.

**Results**: Prevalence of Feld1 is 42 % (181/431). In 54 % of the cases, sensitization to Feld1 is associated with another pets allergen sensitization as lipocalin and serum albumin: Canf5 in 55 % (53/97), Canf1 in 44 % (43/97) and Feld4 in 42 % (41/97) for the most frequent ones. On the 58 patients taken in charge by subcutaneous immunotherapy (SIT) started between 2005 and 2010 for pets allergy, 100 % (58/58) were complaining for cat allergy, 19 % (11/58) were complaining for symptoms for other pets than cat, 55 % (32/58) have asthma associated to rhinitis and 43 % (25/58) have a food complain allergy associated. 97 % of patients are polyallergic with a clinical allergy other than pets. SIT clinical improvement score for pets was 68 % for SIT started in 2005 (end in 2009–2010) compared to 91 % for SIT started in 2010 (end of SIT 2014–2015). Adverse systemic reaction (ASR) were recorded for 7 patients (2 grade II and 5 grade III) without grade IV. All grade III were asthmatic patients.

**Conclusions**: Pets habits as food habits have changed in most urban European areas: they share frequently human life and are indoor allergens. It is shown here that in a multicomplex community like Geneva, most of patients consulting an allergy setting are highly sensitized to Feld1 and have another pets minor allergen’s sensitization associated to Feld1. Once allergic to pets, >90 % of the patients are co-allergic to pollens and food. Immunotherapy being the only treatment to cure the allergic disease [6, 7], improving schedule for individual patient should be an option. Component resolved diagnostic routine used in this setting is able to improve the SIT efficacy from 68 to 91 %, with an average (median) score of improvement for pets allergy by SIT of 84 %. ASR were recorded for 12 % of patients.

SIT for pets is highly efficient and secure despite polysensitization and polyallergy. As for Canf1 for dog allergy [8], Feld1 alone seems not sufficient for diagnosis and consecutively treating cat allergic patient.

**Keywords**: Component resolved treatment; Subcutaneous immunotherapy; Pets allergy; Polysensitization; Molecular allergology

**References**

1. Custovic A, Simpson A, Woodcock A. Importance of indoor allergens in the induction of allergy and elicitation of allergic disease. Allergy. 1998; 53:115–2010.
2. Bukstein D. The Obama dilemma: allergic rhinitis (animal dander allergy)-the great burden of illness. Allergy Asthma Proc. 2009;30(6):567–72. doi: 10.2500/aap.2009.30.3297.
3. Pyrhönen K, Näyhä S, Läärä E. Dog and cat exposure and respective pet allergy in early childhood. Pediatric Allergy Immunol. 2015; 26: 247–255.
4. Gay-Crosier F, Barber D, Bienvenu J. Allergy diagnosis in Geneva area: a complex multi-ethnic community with high pan-allergen prevalence. Clin. Transl. Allergy. 2014;4(Suppl 2): P49.
5. Gay-Crosier F, Barber D, Diaz Perales A. Profilin dominates food allergy phenotype in Geneva Area. In: EAACI Copenhagen 2014. TPS 23-8.06.014.
6. Lockey R, Ledford D. Allergens and Allergen Immunotherapy: Subcutaneous, Sublingual and Oral, 5th Edition Edité par CRC Press, 2014. ISBN 10: 1842145738/ISBN 13: 9781842145739.
7. A European Declaration on Immunotherapy. Designing the future of allergen specific immunotherapy. Clin. Transl. Allergy. 2012; 2:20.
8. Polovic N, Wadén K, Binnmyr J, Hamsten C, Grönneberg R, Palmberg C, Milcic-Matic N, Bergman T, Grönlund H, Van Hage M, Reto Cramer R. Dog saliva—an important source of dog allergens. Allergy. 2013; 68(5): 585–592.

#### P39 Neutrophils are potential antigen presenting cells in IgE- mediated allergy

##### Dominika Polak^1^, Birgit Nagl, Claudia Kitzmüller, Barbara Bohle

###### Medical University of Vienna, Vienna, Austria

**Correspondence**: Dominika Polak

*Clinical and Translational Allergy* 2016, **6(Suppl 2)**:P39

**Background**: Neutrophils are present in large numbers in allergic late-phase reactions. However, it is not yet clear whether they contribute to allergic inflammation. These professional phagocytes might present the allergen to allergen-specific T cells since they express MHC class II molecules upon stimulation with certain cytokines, chemokines and bacterial factors, such as GM-CSF, TNF-a, IL-8, IFN-g and LPS, respectively. In fact, murine neutrophils have been shown to process and present antigens to CD4^+^ T-cells.

Our aim is to assess whether human neutrophils act as antigen-presenting cells for allergen-specific T-cells.

**Materials and methods**: Neutrophils isolated from the peripheral blood of allergic donors were cultured under different conditions and analyzed for the expression of MHC class II, CD40, CD80, and CD86, by flow cytometry. Surface binding, internalization and intracellular degradation of fluorescence-labelled Bet v 1 by neutrophils were compared with monocytes. Microsomal proteases were isolated from both cell types and incubated with Bet v 1. The resulting proteolytic fragments were sequenced using mass spectrometry. Finally, neutrophils and monocytes were cocultured with Bet v 1-specific T-cell cultures generated from birch-pollen allergic donors in the presence or absence of Bet v 1 and proliferative responses of T-cells were assessed.

**Results**: A cocktail of IL-3, GM-CSF and IFN-g enhanced the expression of HLA class II and CD80 on neutrophils. Neutrophils effectively internalized Bet v 1 and their uptake and endolysosomal degradation of the allergen was faster than by monocytes. In addition, neutrophils processed longer peptides of Bet v 1 than monocytes. Neutrophils pulsed with Bet v 1 induced proliferation in Bet v 1- specific T- cells specific for different epitopes distributed over its entire amino acid sequence. However, monocytes were the more potent antigen-presenting cells.

**Conclusions**: Our data provide evidence that neutrophils may serve as antigen-presenting cells for allergen specific T-cells and thereby, play a role in the late phase reaction of IgE-mediated allergy.

**Keywords**: Neutrophils; Antigen presenting cells; IgE mediated

#### P40 Characterization of allergen-specific CD8+ T cells in type I allergy

##### Nazanin Samadi^1^, Claudia Kitzmüller^1^, Rene Geyeregger^2^, Barbara Bohle^1^, Beatrice Jahn-Schmid^1^

###### ^1^Medical University of Vienna, Vienna, Austria; ^2^Children Cancer Research, Vienna, Austria

**Correspondence**: Nazanin Samadi

*Clinical and Translational Allergy* 2016, **6(Suppl 2)**:P40

**Background**: Allergy is an IgE-mediated hypersensitivity reaction against harmless antigens. Like any immune response, allergic reactions are mediated by both innate and adaptive immunity. T cells are part of adaptive immunity and play a main role in the maintenance of IgE-mediated allergy. The function of CD4^+^ T cells in the pathophysiology of allergic disorders has been extensively investigated while the role of CD8^+^ T cells is still poorly understood and controversial. The aim of this project is to characterize allergen-specific CD8^+^ T cells in patients with different allergic manifestations (rhinoconjunctivitis, atopic dermatitis and atopic bronchial asthma).

**Materials and methods**: Different seasonal (birch pollen and grass pollen) and perennial allergens (cat dander and house dust mite) were included. PBMCs from allergic patients were stained with the proliferation dye efluor 670 and incubated with allergen. Proliferating CD3^+^CD8^+^ cells were then assessed for the expression of differentiation markers (CD27, CD28, CD45RO, CXCR3, CRTh2, PD-1), homing factors (CCR4, CD62L, CD29b), intracellular cytokines (IL-4, IL-5, IL-13, IL-17, IL-22 and IFN-γ, TNF-α), and cytotoxic proteins (granzyme B and perforin) by flow cytometry and compared to non-proliferating CD8^+^ T cells.

**Results**: We found significant numbers of proliferating CD8^+^ T cells in all allergic manifestations, however, largest numbers were detected upon stimulation with house dust mite & grass pollen extracts. Furthermore, we observed a higher production of IL-4, granzyme B and perforin in proliferating than non-proliferating CD8^+^ T cells. In addition, a significantly higher expression of CD27, CD45RO, CD62L, and CD29b was detected in allergen-reactive CD8^+^ T cells.

**Conclusions**: So far, we could confirm the existence of allergen-reactive IL-4^+^ CD8^+^ T cells in different allergic manifestations. These cells produce cytotoxic proteins and will be further characterized in future experiments.

### Poster Session 5: Molecular and cellular diagnostic tests

#### P41 Nanofluidic-based biosensors allow quantification of total circulating IgE from a drop of blood in 5 minutes

##### Aurélie Buchwalder, Ariel Gomez, Fabien Rebeaud, Iwan Märki

###### Abionic SA, Lausanne, Switzerland

**Correspondence**: Fabien Rebeaud

*Clinical and Translational Allergy* 2016, **6(Suppl 2)**:P41

**Background**: In a world where information is instantly available, the need to provide rapid, near-patient, quantitative diagnosis is becoming an essential requirement. In response to this demand we have developed biosensors with a nanometric-size reaction chamber that enhances molecular interactions and thereby reduces incubation times of immunoassay from hours to seconds. The first application available for this system quantifies total circulating IgE from as little as 50 ul of blood in 5 min. The patient’s sample is diluted with a solution containing fluorescently-labelled detection biomolecules that specifically bind to IgE, thus forming fluorescent molecular complexes. Through capillarity, the solution enters the biosensor where complexed IgE are captured on the targeted area. Fluorescence is then measured by a miniaturized microscope in the abioSCOPE reading unit, a novel and versatile in vitro diagnostic platform.

**Materials and methods**: Matrix commutability, test linearity, effect of potential interfering substances and a comparison study have been performed to determine key analytical performance characteristics of the test.

**Results**: The test is compatible with capillary blood or with serum or plasma samples. It has an assay measurable range from 10 to 400 kU/l of IgE. High levels of nonIgE immunoglobulins, icteric, hemolytic or lipemic samples do not interfere with test results. A comparison study between total IgE tested in the abioSCOPE against an industry gold standard (ImmunoCAP Total IgE^®^ in Phadia 250) showed an excellent agreement between the two systems, with values of sensitivity, specificity and accuracy of 88.7, 87.5 and 88.2 %, respectively.

**Conclusions**: Thanks to this miniaturized biosensor and the abioSCOPE, non-invasive quantification of protein at the point-of-care is performed in 5 minutes from a drop of whole blood collected from the patient’s fingertip, whereas time-to-results using laboratory-based instruments usually takes hours. Validation of this technology allows the development of allergen-specific IgE tests to aid allergists in making precise and comprehensive allergy diagnoses.

#### P42 Allergen microarray for the analysis of serum IgE binding profile and allergenic activity

##### Jaana Haka^1^, Liisa Hattara^2^, Marika Heikkinen^2^, Merja H Niemi^1^, Juha Rouvinen^3^, Petri Saviranta^2^, Pekka Mattila^1^, Kristiina Takkinen^2^, Marja-Leena Laukkanen^2^

###### ^1^Desentum Oy, Espoo, Finland; ^2^VTT Technical Research Centre of Finland Ltd, Espoo, Finland; ^3^University of Eastern Finland, Department of Chemistry, Joensuu, Finland

**Correspondence**: Jaana Haka

*Clinical and Translational Allergy* 2016, **6(Suppl 2)**:P42

**Background**: More than 25 % of the population suffer from IgE-mediated allergic reactions and the number of allergic people is increasing. Consequently, there is increasing demand for strategies targeting early diagnosis, prevention and treatment of allergic sensitisation. Allergy diagnosis is traditionally carried out using allergen extracts in provocative or serological tests. By using component-resolved diagnostics, however, it is possible to identify the specific disease-eliciting allergen and, thus, to introduce more personalised and effective immunotherapy treatments for sensitised individuals.

**Materials and methods**: A simple and rapid Array-in-Well (AiW) platform provides simultaneous IgE reactivity profiling for multiple allergens using only minute amounts of serum.

**Results**: To establish a proof-of-concept, we printed purified and MS validated recombinant or native allergens representing relevant respiratory allergen sources, such as dog, horse and birch pollen allergens as an array into wells of 96-well microtiter plate. The IgE profiling against these allergens was carried out using serum samples collected from allergic donors. The binding of IgEs present in serum samples to the total of nine allergens present in a single well was measured and subsequently detected by fluorescently labelled human IgE-specific secondary antibodies. Fluorescence imaging of the microarrays was used to quantify the fluorescence intensity in each spot, thus resulting in a quantitative profile of IgEs present in the sample.

The allergenic activity of recombinant allergens in solution was measured by a competitive immunoassay on the above established AiW platform. Two recombinant allergens, wild-type allergen vs. natural hypoallergen, with different allergenic properties were compared by a competitive assay. The binding of serum IgEs to immobilised allergens was inhibited by the increasing amounts of the soluble allergens.

**Conclusions**: The developed allergen microarray provides a promising, simple and cost-effective tool for simultaneous analysis of allergy-associated IgE antibodies enabling also the biological activity measurement of the allergens by the competitive immunoassay. The assay has apparent potential for high-throughput IgE profiling in the large patient cohorts targeting wide variety of allergens as well as the analysis of the allergenic activity of allergens.

#### P43 Generation of a well-characterized panel of periplaneta americana allergens for component resolved diagnosis

##### Stephanie Eichhorn^1^, Isabel Pablos^1^, Bianca Kastner^1^, Bettina Schweidler^1^, Sabrina Wildner^2^, Peter Briza^1^, Jung-Won Park^3^, Naveen Arora^4^, Stefan Vieths^5^, Gabriele Gadermaier^1^, Fatima Ferreira^1^

###### ^1^University of Salzburg, Department of Molecular Biology, Division of Allergy and Immunology, Salzburg, Austria; ^2^Christian Doppler Laboratory for Innovative Tools for the Characterization of Biosimilars, Salzburg, Austria; ^3^Yonsei University College of Medicine, Department of Internal Medicine, Division of Allergy and Immunology, Seoul, Korea; ^4^CSIR-Institute of Genomic and Integrative Biology, Allergy and Immunology Section, New Delhi, India; ^5^Paul-Ehrlich-Institut, Division of Allergology, Langen, Germany

**Correspondence**: Stephanie Eichhorn

*Clinical and Translational Allergy* 2016, **6(Suppl 2)**:P43

**Background**: In tropic and sub-tropic regions American cockroach (*Periplaneta americana*) is a major source of indoor allergens, frequently causing allergic reactions and asthma. From this source no single major allergen is able to predict patients’ sensitization and therefore serve as a marker allergen in allergy diagnosis. As a result there is a need for component resolved diagnosis (CRD) in American cockroach allergy to determine sensitization patterns and point out treatment strategies. Within the ERA-Net New Indigo project GENALL (Genetically engineered allergens for component-resolved diagnosis and immunotherapy of airway allergies) a set of well-characterized recombinant *Periplaneta americana* allergens will be produced.

**Materials and methods**: Per a 1.0103 (residues 197–378), Per a 2.0101, and the C-terminal domain of Per a 3.0101 (residues 426–675), were recombinantly produced in *E. coli* and purified to homogeneity. Proteins were physico-chemically characterized by mass spectrometry (MS), amino acid analysis (AAA) and circular dichroism spectroscopy (CD). The IgE binding capacity of the proteins was determined by ELISA experiments using sera of cockroach allergic patients from India (n = 25) and South Korea (n = 10).

**Results**: In MS the primary sequence of the proteins was confirmed. With AAA the expected amino acid distribution was obtained and the exact protein concentrations were determined. Spectra obtained from CD revealed partially intact secondary structural elements. The ability of the three allergens to bind IgE was confirmed by ELISA. While for Per a 2 and Per a 3 sensitization frequencies ranged from 40 to 60 %, Per a 1 appeared to be a minor allergen in the tested patients’ cohorts, showing reactivity with only one of the tested sera.

**Conclusions**: Recombinantly produced Per a 1, Per a 2 and Per a 3 were physico-chemically characterized, and their IgE binding capacity was shown by ELISA. In further studies, all ten described *Periplaneta americana* allergens will be used for testing large patients’ cohorts from Europe, Asia and America in order to evaluate the sensitization patterns. Cross-reactivity studies between American cockroach allergens and their homologues from German cockroach (*Blattella germanica*) might help to elucidate the primary sensitizer.

The work was funded by the ERA-Net New INDIGO project I 1152 (GENALL) of the Austrian Science Fund (FWF).

**Keywords**: Cockroach allergy; Component resolved diagnosis

#### P44 Improved diagnostic sensitivity of recombinant Api m 1 and Ves v 5 in diagnosis of Hymenoptera venom allergy

##### Mira Silar, Julij Selb, Rok Kogovsek, Mitja Kosnik, Peter Korosec

###### University Clinic for Respiratory and Allergic Diseases Golnik, Golnik, Slovenia

**Correspondence**: Mira Silar

*Clinical and Translational Allergy* 2016, **6(Suppl 2)**:P44

The published version of this abstract can be found at [1].

**Reference**

1. Šelb J, Kogovšek R, Šilar M, Košnik M, Korošec P. Clinical Exp Allergy. 2016; 4:621*–*630.

#### P45 Added value of biomarkers of primary sensitization and cross-reactivity in patients with hymenoptera venom allergy

##### Leticia Pestana^1^, Alcinda Campos Melo^2^, Ana Mendes^3^, Maria Elisa Pedro^3^, Manuel Pereira Barbosa^4^, Maria Conceição Pereira Santos^5^

###### ^1^Immunoallergology’s Department, Hospital de Santa Maria, CHLN, E.P.E., Lisboa, Portugal, lisboa, Portugal; ^2^Laboratory of Clinical Immunology, Faculdade de Medicina, Universidade de Lisboa. /Instituto de Medicina Molecular, lisboa, Portugal; ^3^Immunoallergology’s Department, Hospital de Santa Maria, CHLN, E.P.E, lisboa, Portugal; ^4^Immunoallergology’s Department, Hospital de Santa Maria, CHLN, E.P.E, Lisboa, Portugal; ^5^Laboratory of Clinical Immunology, Faculdade de Medicina, Universidade de Lisboa. /Instituto de Medicina Molecular, Lisboa, Portugal

**Correspondence**: Maria Elisa Pedro

*Clinical and Translational Allergy* 2016, **6(Suppl 2)**:P45

**Background**: In most cases, the diagnostic tests with conventional extracts are often positive for Bee (B), Wasp (W) and Polistes (P) venom, which may be due to double sensitization or cross-reactivity, stickling the decision of specific immunotherapy (SIT).

**Materials and methods**: Characterization of sensitization profile through the use of recombinant allergens for diagnosis of hymenoptera venom allergy, identifying dual sensitization or cross-reactivity. Contribution of basophil activation test (BAT) in patients (pts) with systemic reactions and both negative skin test (ST) and specific IgE (sIgE). 112 pts (74 men, 38 women, 21–78 years) with systemic reactions to Hymenoptera stings, not submitted to SIT, and 20 atopic controls without history of reaction to insect stings. ST were performed with commercial extracts (CE) of Bee (B), Wasp (W) and Polistes (P) venom, sIgE to the same extracts and recombinant allergens: Api m1, Ves v1, Ves v5 and Pol d5 and CCDs (ImmunoCapÒ). In BAT both venoms were tested in six concentrations: 0.01; 0.02; 0.04; 1.0; 2.5 and 5 ug/ml (Flow2 CAST^®^).

**Results**: Based on sIgE, 50pts (45 %) were polisensitized to B, W, P and 55 pts (49 %) were monosensitized: 30 (B), 20 (W), 5 (P). In 7 pts (6 %) ST and sIgE were both negative. Of the 55 polisensibilized pts, 23 (42 %) had double sensitization (positive sIgE to Api m1 and Ves v1); the remaining 32 (58 %) revealed cross-reactivity (15 Api m1 and 16 Ves v1/Vesv5 and 1 Pol d5). The monosensitized pts to W/P had positive sIgE to Ves v1 and/or Ves v5 and Pol d5. 25 (83 %) of the sensitized pts to B had positive Api m1. The sIgE to CCDs was positive in 15 pts, all with double sensitization with CE, indicating cross-reactivity between species. The 7 pts with negative sIgE had negative recombinants. The specificity for the four components was high, since monosensitized pts showed no positive sIgE for other species, and only a positive control had a positive sIgE to Ves v1/Ves v5. The BAT was performed in 5 pts (with negative ST and sIgE) and it was positive in 3, at concentrations of 0.04 and 2.5 ug/ml for B venom.

**Conclusions**: The determination of sIgE to Api m1, Ves v1/Ves v5 and Pol d5 is a useful diagnostic method allowing discrimination between dual primary sensitization and cross-reactivity, as well as a better selection of patients for SIT. The BAT may be useful in cases of difficult diagnosis.

#### P46 Cosensitization to Alt a 1 and Act d 2: more than a fortuitous association?

##### Françoise Bienvenu^1^, Claire Goursaud^1^, Lorna Garnier^1^, Sandrine Jacquenet^2^, Michaël Degaud^1^, Sébastien Viel^1^, Annick Barre^3^, Pierre Rougé^3^, Jacques Bienvenu^1^, Joana Vitte^4^

###### ^1^Immunology Laboratory, Centre Hospitalier Lyon Sud, Hospices Civils de Lyon, Pierre-Benite, France ; ^2^Genclis SA, Vandoeuvre-Lès-Nancy, France ; ^3^UMR152 IRD-UPS, Toulouse, France; ^4^Immunology Laboratory, Hôpital de la Conception, Assistance Publique-Hôpitaux de Marseille, Marseille, France

**Correspondence**: Françoise Bienvenu

*Clinical and Translational Allergy* 2016, **6(Suppl 2)**:P46

**Background**: The ISAC^â^ microarray explores sensitization to 112 molecular allergens. The analysis of such a broad panel is useful for the detection of overlooked associations. We report cosensitization to Alt a 1 (*Alternaria alternata* acidic glycoprotein) and Act d 2 (kiwi *thaumatin-like protein* TLP), the only TLP tested by ISAC^â^.

**Objectives**: - to study the frequency of Alt a 1 and Act d 2 cosensitization.

 - to try and understand its mechanism

**Materials and methods**: Data from 807 ISAC^â^ 112 microarrays (Thermo Fisher) were collected:

221 patients from Lyon, Central France (62 % males, mean age: 12 years)

586 patients from Marseille, Southern France (51 % males, mean age: 13 years)

**Results**: In both centers, more than 80 % of patients sensitized to Act d 2 were also sensitized to Alt a 1 (Lyon 82 %, Marseille 86 %). Conversely, only 35 % of Alt a 1 sensitized patients from Lyon were also sensitized to Act d 2, but the percentage reached 61 % in Marseille patients. The median level of Alt a 1 specific IgE (sIgE) was significantly higher in cosensitized patients than in Alt a 1 monosensitized patients: 5.4 ISU versus 1.99 for Lyon (p < 0.01) and 17.3 ISU versus 3.7 for Marseille (p < 0.0001).

Importantly, cosensitization to Alt a 1 and other kiwi allergens present on the microarray (Act d 1, Act d 5, Act d 8) was infrequent.

Clinical data were not available for all patients. Act d 2 sensitization was not always clinically relevant with respect to kiwi fruit. Most patients cosensitized to Alt a 1 and Act d 2 displayed respiratory symptoms.

**Discussion**: Gomez-Casado C. (*FEBS Letters 2014*) demonstrated that:

- Alt a 1 is co-localized with Act d 2 in kiwi
- electrostatic interactions exist between Act d 2 and Alt a 1
- Alt a 1 behaves as a competitive inhibitor of the TLP.

Moreover, Act d 2 spotted on ISAC^â^ is a natural purified allergen (as opposed to Alt a 1 which is a recombinant one). Therefore, Act d 2 might be contaminated by Alt a 1 during the purification process which could induce recognition of Act d 2 spots by Alt a 1 sIgE. Alternatively, true cross-reactivity between Act d 2 and Alt a 1 cannot be excluded at this stage.

**Conclusions**: Considering its frequency, Alt a1/Act d 2 cosensitization observed in 2 centers is unlikely to be incidental. The mechanism underlying this finding needs further investigation, including the search for (i) Alt a 1 association with other TLP, (ii) structurally similar epitopes, and (iii) clinical relevance of Act d 2 sensitization.

**Keywords**: Act D 2; Alt A 1; Kiwi; Alternaria; Thaumatin-like-protein

#### P47 Molecular diagnosis for peanut allergy: ALFA method performs as well as established methods for Ara h 1, Ara h 2, Ara h 6, Ara h 9 and CCD

##### Amel Bensalah^1^, Isabelle Cleach^1^, Laurent Mousseau^2^, Chantal Agabriel^3^, Valérie Liabeuf^4^, Joëlle Birnbaum^4^, Jean-Louis Mège^1^, Joana Vitte^1^

###### ^1^Hôpital de la Conception, Laboratoire d’Immunologie, Marseille, France, Metropolitan; ^2^Galenus Regeneratio, Tours, France, Metropolitan; ^3^Hôpital de la Timone, Service de Pédiatrie Multidisciplinaire, Marseille, France, Metropolitan; ^4^Hôpital de la Timone, Service de Dermatologie-Vénéréologie, Marseille, France, Metropolitan

**Correspondence**: Amel Bensalah

*Clinical and Translational Allergy* 2016, **6(Suppl 2)**:P47

**Background**: Molecular allergy diagnostics is currently a well-established, valuable tool for classifying peanut-related reactions in either genuine peanut allergy or cross-reactions. This is an important point, given the difference in further severity evaluation, prognostic prediction, and therapeutic management of the true peanut allergic patient.

**Materials and methods**: We took advantage of the singleplex ALFA method (DrFooke) in order to study the performances of most important peanut allergens (Ara h 6, Ara h 2, Ara h 1, Ara h 9) in peanut allergic or peanut tolerant patients. Serum specific IgE (sIgE) to cross-reactive carbohydrate determinants (CCD) and raw peanut extract were also assessed. ALFA results for sIgE (colorimetric result units) were compared to ImmunoCAP 250 (expressed in kU_A_/L) and ImmunoCAP ISAC (expressed in ISU) sIgE data for the same serum samples from 19 peanut-allergic patients (9 boys) with a median age of 10 years (2–19). For semiquantitative comparison, we defined 7 classes for each method, based on the intensity of the sIgE signal.

**Results**: Concordance between two methods was considered as ideal if the results fit in the same class, good if the difference was ±1 class, and poor for ±2 classes. Results were considered as discordant when the difference was ≥2 classes (Table 4). No positive/negative discordance was seen for Ara h 2 and Ara h 6. Ideal or good concordance for these two allergens ranged from 74 to 89 %. On the contrary, despite 50–68 % ideal or good concordance for Ara h 1 and Ara h 9, positive/negative discordance was frequent for these allergens, with ALFA method yielding more positive results than both ISAC and ImmunoCAP.

**Table 4:** Comparison of peanut allergen sIgE measured with three methods: ALFA, ImmunoCAP®, ISAC®

Methods	Allergens	Concordance (%)				Positive/negative discordance
		Ideal	Good	Poor	None	
ALFA® and singleplex ImmunoCAP®	Ara h 2	26	63	11	0	0
	Ara h 1	41	24	12	24	9, with 4 ALFA positive, ImmunoCAP negative, and 1 ALFA negative, ImmunoCAP positive
	Ara h 9	22	44	28	6	7, with 5 ALFA positive, ImmunoCAP negative, and 2 ALFA negative, ImmunoCAP positive
	CCD	53	33	13	0	4, all ALFA positive, ImmunoCAP negative
	Peanut extract	17	41	41	6	0
ALFA® and ISAC® microarray	Ara h 2	42	32	16	11	0
	Ara h 6	16	58	11	16	0
	Ara h 1	17	33	28	22	9, all ALFA positive, ISAC negative (4 ImmunoCAP positive, 4 ImmunoCAP negative, 1 not assayed with ImmunoCAP)
	Ara h 9	44	17	28	11	9, all ALFA positive, ISAC negative (4 ImmunoCAP positive, 5 ImmunoCAP negative)
	CCD	33	39	22	6	10, all ALFA positive, ISAC negative (4 ImmunoCAP positive, 4 ImmunoCAP negative, 2 not assayed with ImmunoCAP)

For each method, sIgE results were converted in 7 classes (0 to 6) based on the intensity of the measured signal. Ideal concordance was defined as two same-class results, good concordance as adjacent class results, poor concordance as a difference of 2 classes, and no concordance when results differed by more than 2 classes

**Conclusions**: Molecular allergy diagnostics for peanut allergy with the ALFA method performs well in comparison with other methods. In particular, Ara h 2 and Ara h 6, peanut major allergens in both temperate and mediterranean environments, display a very good correlation with both ImmunoCAP ISAC and singleplex ImmunoCAP. No false positive or false negative result was reported for these two allergens in our patient group. In conclusion, the Dr. Fooke ALFA kit is a reliable tool for peanut molecular allergy diagnostics.

**Keywords**: Peanut allergy; Ara H 1; Ara H 2; Ara H 6; Ara H 9

#### P48 Evaluation of a food challenge service in relation to specific IgE to molecular components in children with suspected peanut allergy

##### James Gardner^1^, Minal Gandhi^2^, Harsha Kariyawasam^3^, Giuseppina Rotiroti^4^

###### ^1^Great North Children’s Hospital, Newcastle Upon Tyne, United Kingdom; ^2^Royal Free Hospital, London, United Kingdom; ^3^Royal National Throat Nose and Ear Hospital, London, United Kingdom; ^4^Royal National Throat, Nose and Ear Hospital, London, United Kingdom

**Correspondence**: James Gardner

*Clinical and Translational Allergy* 2016, **6(Suppl 2)**:P48

**Background**: The aim of this study was to ascertain if negative specific IgE to peanut component Ara-h2 is able to predict peanut tolerance whilst positive specific IgE to Peanut Ara-h2 is more likely associated with peanut allergy in a population of cosmopolitan children with suspected peanut allergy, resident in the central and Greater London region and attending a tertiary level allergy clinic.

**Materials and methods**: The study is a retrospective evaluation of peanut challenge cases in relation to peanut challenge outcomes, results of peanut skin prick test and peanut specific IgE and specific IgE for peanut molecular components (Ara-h2 and Ara-h8). In addition children with a recent (within 12 months) history of peanut reaction for whom specific IgE for peanut molecular component was available were also included in the analysis.

**Results**: The analysis of our data suggests that specific IgE to Ara h 2 is a more specific test for predicting outcome of a peanut food challenge when compared to peanut specific IgE, with specificities ranging from 85 to 95 %, depending on the cohort examined. These similar values across cohorts are reflected in this study data. In our peanut allergic subjects the median value for Ara h2 was 6.7 and 0.09 kua/l in the peanut tolerant group. This was found to be statistically significant (p = <0.0001). The AUC for was found to be 0.91 and therefore significant in predicating peanut challenge outcome.

**Conclusions**: Different cut off points for SPT and specific IgE have been presented in a number of studies, but so far either a low sensitivity or low specificity has reduced the reliability as a replacement for oral challenges and thereby the clinical impact. The usage of component-resolved diagnostics have been reported as more useful compared to whole peanut protein specific IgE. In line with other studies we found that Ara h 2 was the best predictor of peanut challenge outcomes. Whilst the results of the study do not reduce the need for challenging patients, the need for food challenges may be reduced by combing the results of Ara H2 IgE and clinical history.

#### P49 Component resolved diagnosis in cereal allergy

##### Isabel Carrapatoso, Celso Pereira, Frederico Regateiro, Emília Faria, Ana Todo-Bom

###### Immunoallergy Department. Coimbra University Hospital Centre, Coimbra, Portugal

**Correspondence**: Isabel Carrapatoso

*Clinical and Translational Allergy* 2016, **6(Suppl 2)**:P49

**Background**: Component resolved diagnosis (CRD) provides a step forward in identifying trigger allergens and assessing the risk of severe reactions in food allergy. This study aims to characterize, over a one-year period, the clinical and molecular profiles of patients with immediate food reactions to cereals, followed in a Food Allergy outpatient consultation.

**Materials and methods**: Ten patients (8 males) with an average age of 42 ± 12 years were included. We recorded the first food/drink containing cereals associated with clinical manifestations of food allergy and screened potential co-factors involved in the reactions. Our investigation included the evaluation of symptoms after ingestion of other foods and related to inhalant allergens. Skin prick tests (SPT) to cereal flours, to inhalant allergens and to Pru p 3 were performed. We carried out specific IgE (sIgE) determinations to cereal flours and to some components: Gluten, Tri a 19, Tri a 14, Pru p 3 and Pru p 4. We performed additional SPT, prick-to-prick tests and sIgE in selected cases, depending on clinical history.

**Results**: Two patients described symptoms after drinking beer and other two after ingesting pizza. One patient referred wheezing after eating corn or bread when exercising. The remaining patients developed food reactions after eating meals containing mixed foods that always included cereals, particularly wheat. Nine patients had systemic reactions and severe anaphylaxis occurred in six of them. In the four patients with positive sIgE to Tri a 19, anaphylaxis only occurred associated with exercise. However, two of them had recurrent urticaria while treated with low doses of aspirin. Four patients had sIgE to Tri a 14. Two of them described systemic reactions with variable severity and even anaphylaxis when co-factors such as exercise, alcohol or drug intake were present. One patient was only sensitised to gluten. All the patients sensitised to Tri a 14 had sIgE to Pru p 3. Besides cereal allergy, most of the patients had other food allergies. Seven patients had respiratory allergies related to inhalant allergens.

**Conclusions**: Cereal allergy diagnosis is a clinical challenge. Sensitisation to Tri a 19 seems to be directly related to wheat- dependent exercise induced anaphylaxis, but for those positive to Tri a 14 other different components and co-factors may be crucial for clinical severity. CRD improved the accuracy of cereal allergy diagnosis, particularly wheat allergy.

### Poster Session 6: Molecular diagnosis in prevention and therapy

#### P50 Pretreatment molecular sensitizations determine the sIgG4 induction during the updosing of SCIT and may be useful to identify clinically relevant additional sensitizations

##### Johannes Martin Schmid^1^, Ronald Dahl^2^, Hans Juergen Hoffmann^1^

###### ^1^Aarhus University Hospital, Aarhus, Denmark; ^2^Odense University Hospital, Odense, Denmark

**Correspondence**: Johannes Martin Schmid

*Clinical and Translational Allergy* 2016, **6(Suppl 2)**:P50

**Background**: Allergen immunotherapy is an effective treatment of allergic rhino-conjunctivitis. Clinical efficacy is associated with improvement of basophil sensitivity and an increase in allergen specific immunoglobulin concentration.

**Objective**: Can assessment of allergen component sIgE be used to diagnose clinically relevant allergic sensitizations?

Can pretreatment component specific sensitizations predict IgG4 responses during updosing of AIT/SCIT?

**Materials and methods**: Twenty-four subjects with grass pollen allergic rhino-conjunctivitis were randomized 3:1 to receive AIT/SCIT (Alutard SQ) or to an open control group. sIgE and specific IgG4 concentrations were determined for eight grass pollen molecules and for additional sensitizations by ISAC before treatment and after updosing.

**Results**: The absence of pretreatment component sIgE had in general a good negative predictive value (58.3–100 %) for clinically relevant allergies reported by the patients in a standardized questionnaire, while sIgE sensitizations only predicted clinical relevant allergies to a lesser extent. sIgE against eight grass allergen molecules decreased from a median 4.6 ISU before treatment to 2.14 ISU (p < 0.0001, n = 102) after updosing of AIT. The updosing phase induced a sIgG4 increase from a median 0 ISU pretreatment to 0.83 ISU after updosing (p < 0.0001, n = 102), but only for allergen molecules to which pretreatment sIgE was detected (Spearman’s σ = 0.72, p < 0.0001, n = 102).

**Conclusions**: Assessment of component resolved sIgE is useful to confirm or rule out clinical relevant allergies. Pretreatment allergen component sIgE appears to determine the induction of sIgG4 in the updosing phase, and may be useful to predict response to AIT.

**Keywords**: Molecular allergy; Grass pollen allergy; SCIT; Allergic rhino-conjunctivitis

#### P51 Usefulness of recombinant latex allergens in immunotherapy’s decision and follow-up

##### Inês Mota, Filipe Benito Garcia, Angela Gaspar, Mário Morais-Almeida

###### Immunoallergy Department, CUF Descobertas Hospital, Lisbon, Portugal

**Correspondence**: Inês Mota

*Clinical and Translational Allergy* 2016, **6(Suppl 2)**:P51

**Background**: Latex allergy affects mainly selected populations, called high-risk groups. Latex sensitization profile differs between these risk groups, but the existence of different profiles is not an aspect taken into consideration for the vaccine production. The commercially available immunotherapy extract is mostly constituted by Hev b 6 and Heb b 5 proteins. We aimed to describe the relevance of recombinant latex allergens for the specific immunotherapy’s decision and follow-up.

**Materials and methods**: We describe the clinical cases of 3 patients, who were successful desensitized with sublingual latex-immunotherapy (SLIT 100 % Latex, ALK-Abelló^®^). Before immunotherapy (T0) all patients were tested with skin prick tests to latex and foods with latex cross-reactivity responsible for latex-fruit syndrome (LFS), latex-specific IgE (LsIgE) and single recombinant latex allergens panel (rHev b 1, rHev b 3, rHev b 5, rHev b 6.01, rHev b 8, rHev b 9 and rHev b 11). This evaluation was repeated after a course of immunotherapy with at least 3 years duration.

**Results**: Case 1: 41-year-old woman with history of 15 surgeries and latex-induced anaphylaxis (LIA) and LFS (anaphylaxis with banana and passion fruit). Laboratorial evaluation T0/after 44 months of SLIT was: LsIgE 17.2/1.04 kU/L, rHev b 1 0.36/<0.10 kU/L, rHev b 3 <0.35 kU/L, rHev b 5 15.9/0.54 kU/L, rHev b 6.01 17.3/1.06 kU/L, rHev b 9 2.02/<0.10 kU/L, rHev b 11 1.36/0.97 kU/L. Symptomless ingestion of banana and only with rhinoconjunctivitis by accidental inhalation of latex. Case 2: 59-year-old man, surgeon, with LIA and LFS (anaphylaxis with avocado, banana and bell pepper). Laboratorial evaluation T0/after 42 months of SLIT: LsIgE 14.6/1.68 kU/L, rHev b 5 1.94/<0.10 kU/L; rHev b 6.01 2.73/2.01 kU/L, rHev b 1, rHev b 3, rHev b 8, rHev b 9, rHev b 11 <0.10 kU/L. Without symptoms on accidental latex contact and after chestnut and bell pepper ingestion. Case 3: 43 year-old woman, nurse, with LIA. Laboratorial evaluation T0/after 60 months of SLIT: LsIgE 10.5/<0.10 kU/L, rHev b 5 1.48/<0.10 kU/L; rHev b 6.01 1.20/<0.10 kU/L, rHev b 1, rHev b 3, rHev b 8, rHev b 9, rHev b 11 <0.10 kU/L. Asymptomatic latex contact.

**Conclusions**: Evaluation of latex sensitization profile should always be performed before latex immunotherapy’s decision. Recombinant latex allergens (rHev b 5 and rHev b 6.01) showed to be essential to decide latex immunotherapy, as well as to assess the achievement of a successful desensitization.

**Keywords**: Latex allergy; Recombinant latex allergens; Immunotherapy

#### P52 Omega-5-gliadin in the diagnosis of wheat-dependent anaphylaxis induced by ibuprofen but not by exercise

##### Joana Cosme^1^, Letícia Pestana^1^, Amélia Spínola Santos^1^, Manuel Pereira Barbosa^2^

###### ^1^Immunoallergy Department - Hospital de Santa Maria – Centro Hospitalar Lisboa Norte, Lisbon, Portugal; Faculdade de Medicina de Lisboa, Lisbon, Portugal; ^2^Immunoallergy Department - Hospital de Santa Maria – Centro Hospitalar Lisboa Norte, Lisbon, Portugal; Faculdade de Medicina de Lisboa; Faculdade de Medicina de Lisboa, Lisbon, Portugal

**Correspondence**: Joana Cosme

*Clinical and Translational Allergy* 2016, **6(Suppl 2)**:P52

**Background**: Nonsteroidal anti-inflammatory drugs (NSAIDs) can enhance the symptoms of wheat-dependent exercise-induced anaphylaxis (WDEIA). Only a few reports of wheat-dependent anaphylaxis induced by the combination of wheat and NSAIDs without exercise have been made.

**Materials and methods**: Demonstrate the role of *rTri a 19* (ω-5-gliadin) in the diagnosis of wheat-dependent anaphylaxis induced by NSAIDs without exercise as a cofactor.

**Results**: Case report of 36 years-old man with allergic (mites) rhinosinusitis, referred to our Immunoallergy department for an episode of generalized urticaria, itching, dyspnea and hypotension that was preceded, 30 minutes earlier, by the ingestion of wheat bread with butter followed by one ibuprofen tablet. By that time, he was in the eighth day of antibiotherapy with amoxicillin/clavulanic acid and in third day of ibuprofen—600 mg every 12hours, for a dental infection. The patient was admitted in the emergency department and the symptoms disappeared after administration of intramuscular adrenaline, intravenous antihistamines and corticosteroids. He refers no relation of this event with physical activity and had no previous complaints with wheat ingestion with or without associated exercise. SPT showed positive results to dust mite, shrimp, tropomyosin and wheat (wheal 4 mm) but negative to gliadin extract. Prick-prick reaction to wheat flour was positive (wheal 8 mm). The levels of serum total IgE was >2000 UI/mL and sIgE to wheat, gluten and *rTri a 19* (ω-5-gliadin) were 4.67, 7.7 and 21.60 KUA/L respectively. Using the ImmunoCAP ISAC (Thermo Fisher Scientific, Sweden) there were determined: *rTri a 19* (ω-5-gliadin) of 9.2 ISU-E and *nFag e 2, rTri a 14 and nTri a aA_TI* all negatives. Exercise challenge tests were performed without and with prior wheat ingestion were both negative. There were also performed an oral provocation test with meloxicam without wheat that was negative. The diagnosis of wheat-dependent anaphylaxis induced by NSAIDs was made and the patient was prescribed with an epinephrine auto-injector 0.3 mg. Recommendations to avoid taking NSAIDs 3 h before and after wheat ingestion were made.

**Conclusions**: NSAIDs are common triggers of WDEIA but its role as cofactor of wheat-dependent anaphylaxis is not well studied. We present a case of wheat-dependent anaphylaxis induced by NSAIDs where the determination of *rTri a 19* (ω-5-gliadin) was an important toll in the diagnosis.

**Keywords**: RTri A 19; Omega-5-gliadin; Wheat, Anaphylaxis; Nonsteroidal anti-inflammatory drugs

#### P53 Food dependent exercise-induced anaphylaxis: a component-resolved and in vitro depletion approach to access IgE cross-reactivity

##### Diana Silva^1^, Teresa Vieira^2^, Ana Maria Pereira^3^, André Moreira^4^, Luís Delgado^4^

###### ^1^Serviço de Imunoalergologia, Centro Hospitalar São João, Porto, Portugal; ^2^Allergy and Clinical Immunology Department, Unidade Local de Saúde do Alto Minho, Viana Do Castelo, Portugal; ^3^Immunology Lab, Department of Clinical Pathology, Centro Hospitalar São João, Porto, Portugal; ^4^Laboratory of Immunology, Basic and Clinical Immunology Unit, Faculty of Medicine Porto University, Porto, Portugal

**Correspondence**: Diana Silva

*Clinical and Translational Allergy* 2016, **6(Suppl 2)**:P53

**Background**: Provocation tests for food dependent exercise induced anaphylaxis (FDEIA) evaluation are not deprived of side effects and have a high rate of false negatives. This is particularly challenging in individuals sensitized to panallergens.

**Aim**: To assess IgE cross-reactivities using *in-vitro* depletion in patients sensitized to LTP with an history of FDEIA.

**Materials and methods**: Three patients with history of FDEIA, sensitized to LTP were studied. A 25-year-old man, with cholinergic urticaria and mild oral allergy syndrome to peach, who suffered an episode of generalized urticaria and angioedema during a recreational soccer match, preceded by peach-based soft drink ingestion; a 19-year-old woman, sensitized to house dust mite and pollens with previous history of allergic rhinitis and controlled asthma, suffered an anaphylactic shock while playing soccer, having ingested walnuts in the previous 90 min and a 57-year-old man with bakers-asthma with four episodes of anaphylaxis during exercise after ingesting wheat-containing food. All individuals performed diagnostic work-up with skin prick tests (SPTs) and specific IgE (sIgE) for baseline characterization and LTP sensitization confirmation. For in vitro immune-depletion procedure, duplicate samples of each patient serum were pre-incubated with the suspected native allergen (peach, walnut or wheat, respectively) in a solid phase (ImmunoCAP^®^). The eluted serum, containing unbound IgE, was collected and samples were re-tested and compared using ImmunoCAP ISAC^®^.

**Results**: The first patient showed LTP sensitization to Prup-3, Cor-a-8, Jug-r-3, Arah-9; after pre-incubation with peach there was 100 % depletion of sIgE to all LTPs. The second patient had SPTs and sIgE positive to *Rosaceaes*, non *Rosaceae* fruits and nuts, including mango, orange and walnuts; ISAC detected LTP sensitization (Prup-3, Cor-a-8, Jug-r-3, Arah-9); walnut depleted sIgE by 60 % to Ara-h-9, 67 % to Prup-3 and Pla-a-3, 75 % to Artv3, 88 % to Jugr-3 and 100 % to Cor-a-8. The patient with previous baker-asthma, SPTs and sIgE were positive to wheat, although ω-5-gliadin was negative; ISAC showed sensitization to Prup-3, Jug-r-3, Arah-9, Tri-a-14; wheat immunodepletion occurred by 100 % only to Tri-a-14.

**Conclusions**: *In-vitro* immunodepletion may be an useful assay to guide the diagnostic study, deciding which allergens to test, and also eviction recommendations in patients with FDEIA sensitized to a panallergen like LTP.

#### P54 Olive pollen allergens: what are we missing?

##### Sara Prates, Cátia Alves, Elena Finelli, Paula Leiria Pinto

###### Hospital Dona Estefânia - Centro Hospitalar Lisboa Central, Lisbon, Portugal

**Correspondence**: Sara Prates

*Clinical and Translational Allergy* 2016, **6(Suppl 2)**:P54

**Background**: In the south of Portugal, grass and olive tree are the most common pollen allergens. In a previous study in pollen polysensitized patients, when comparing the results of SPT (skin prick tests) and ISAC (ImmunoCAP-ISAC) we observed an excellent concordance between the two tests for grass but not for olive pollen. The aim of this study was to characterize the pattern of sensitization to molecular allergens in patients with positive SPT to olive and pollen polysensitization.

**Materials and methods**: We did a retrospective analysis on a sample of 42 patients with asthma and/or rhinitis, with positive SPT to olive and at least one more pollen, who had specific IgE detection by ISAC. We analized the results of ISAC, focusing on olive pollen specific allergens (Ole e 1, Ole e 7 and Ole e 9) and crossreactive allergens.

**Results**: Mean age was 22 years (ranging from 4 to 52 years) and 52 % were females. Besides olive, SPT was positive to grass in 95.2 %, plantain in 45.2 %, birch in 35.9 %, plane tree in 35.9 % and to other pollens in less than 30 % each. In ISAC, 50 % (n = 21) were positive to Ole e 1 and 7 % (n = 3) reacted to Ole e 7, corresponding to 22 olive positive patients (Olea+). No patient reacted to Ole e 9. Overall, 48 % (n = 20) had no detectable IgE to the olive pollen allergens present in ISAC (Olea−). We found co-sensitization to grass allergens in the majority of our sample: 82 % of Olea+ patients and 100 % of Olea− patients. Olea+ patients were sensitized to profillins and/or polcalcins in 36 % of cases; Olea− patients in 50 % of cases. We found no sensitization to any crossreactive allergen (profillins, polcalcins, PR-10 or LTP) in 41 % of Olea+ and in 45 % of Olea− patients. In this sample of 42 patients with positive SPT to olive pollen, ISAC detected no IgE, neither to olive species specific nor to crossreactive molecules in 21 % (n = 9).

**Conclusions**: In polysensitized patients, positive SPT in the absence of species specific molecules is attributed to the presence of sensitization to panallergens and not to the allergen source. In our sample of patients apparently sensitized to olive the percentage of sensitization to olive species specific allergens was low and in almost half of these there was also no reactivity to panallergens which could explain the positive SPT. These results suggest that we might be missing, in ISAC, an olive species specific allergen rellevant to our population.

**Keywords**: Olive pollen; Mollecular allergens; Panallergens; Diagnosis

#### P55 Purified Alt a 1 extract in *Alternaria alternata* allergy diagnosis

##### Bárbara Kong Cardoso, Cíntia Cruz, Filipa Semedo, Elza Tomaz, Filipe Inácio

###### Centro Hospitalar de Setúbal, Setúbal, Portugal

**Correspondence**: Bárbara Kong Cardoso

*Clinical and Translational Allergy* 2016, **6(Suppl 2)**:P55

**Background**: *Alternaria alternata* (Aa) has been considered a relevant allergen in airways atopic disease and specific immunotherapy may be indicated. Alt a 1 is the major allergen and immunotherapy extracts standardization is based on it. On the other hand sensitization to minor allergens may compromise the efficacy and/or the safety of the treatment. Our aim was to estimate the prevalence of Aa sensitization among atopic patients in Portugal, as well as compare the use of a total Aa extract to a purified Alt a 1 extract in the diagnosis.

**Materials and methods**: We performed skin prick tests in 102 consecutive patients referred to our outpatient allergy clinic using our standard battery of aeroallergen extracts and also included Aa total and Alt a 1 Diater^®^ extracts. Papules with medium diameter 3 mm or more were considered as positive tests. We characterized Aa sensitive patients and analysed differences between Aa total and Alt a 1 results.

**Results**: Sixty patients had at least one positive test, thus being atopic. Twenty one (35 %) atopic patients were positive to Aa total extract and 17 (28 %) to Alt a 1 extract. Aa sensitive patients were 13 female, 8 male, aged 7 to 66 years, mean 23.2 ± 16.0 and 43 % of them had asthma; Aa non-sensitive atopic patients were 15 female, 24 male, aged 2–64 years, mean 20.6 ± 17.0, 39 % having asthma. Relating Alt a 1 to Aa results 13 patients showed differences between the two, variation of medium diameter of papules ranging from −100 to +50 %.

**Conclusions**: Prevalence of Aa sensitivity was fairly high in the studied group −35 %. We found no statistical significant differences between Aa sensitive *versus* non-sensitive patients concerning age, sex and pathologies. Seventeen (81 %) Aa sensitive patients had positive test to Alt a 1. Differences in skin prick test results between Aa and Alt a 1 extracts support a frequent sensitivity to Aa minor allergens and point to the advantage of using purified Alt a 1 extracts in the diagnosis and in immunotherapy for Aa allergy.

**Keywords**: *Alternaria alternata*; Alt A 1

#### P56 Use of specific IgE Bos d8 (casein) to aid early introduction of dietary baked milk in children with cows’ milk allergy

##### James Gardner^1^, Santanu Maity^2^, Giuseppina Rotiroti^3^, Minal Gandhi^2^

###### ^1^Great North Children’s Hospital, Newcastle, United Kingdom; ^2^Royal Free Hospital, London, United Kingdom; ^3^Royal National Throat Nose and Ear Hospital, London, United Kingdom

**Correspondence**: James Gardner

*Clinical and Translational Allergy* 2016, **6(Suppl 2)**:P56

**Background**: Casein (Bos d8) is identified as major allergen in cow’s milk and in high levels often indicates allergy to both fresh and baked milk. It may be used to predict and aid the management of acute cow’s milk allergy in children, with low levels indicating a tolerance to baked milk products

Casein is a heat stable protein and the proteins remain stable even after 120 mins boiling at 100 °C. This is unlike α-lactalbumin which disappears after 30 mins, β-lactoglobulin after 15 mins and Lactoferrin after 10 mins.

A large study suggested that 75 % of children with a recent diagnosis of cow’s milk protein allergy tolerated baked milk. Tolerance of baked milk is a good indicator for outgrowing allergy and with the addition of baked milk into the diet appears to accelerate tolerance. Although there is a paucity of published evidence to support the practice, home reintroduction of baked milk products has become routine practice through experience in allergy services in the UK. There are limited cut-off levels for casein published in literature.

We have audited the use of casein spIgE in managing children with cows’ milk allergy in our UK based population.

**Materials and methods**: Casein spIgE testing was incorporated into our evaluation of children with cow’s milk allergy. Supervised baked milk challenges were performed on selected children using ‘malted milk’ biscuits.

**Results**

- A total of 98 tests were requested over a period of 7 years.
- 42 children with confirmed cows’ milk allergy were challenged.
- 29 of them had eczema.
- Their mean age was 4.7 years, median 3 years (Range 1–16 years).
- SPT was performed to fresh and cows’ milk reagent (Diagenics)
- 41 children passed and successfully introduced baked milk and remain symptom free with regular exposure.
- 9 of these children have outgrown their milk allergy and tolerate fresh milk.
- See Tables 5 and 6 of results

**Table 5:** Result values for children passing baked milk challenge

	Mean	Median	Range
Fresh Milk SPT	11.25 mm	10 mm	3–33 mm
Cow’s Milk reagents SPT	3.68 mm	3.04 mm	0–10.8 mm
SpIgE Cow’s milk	5.61 iu	2.80 iu	0–37.2 iu
SpIgE Casein	2.20 iu	0.55 iu	0.02–29.8 iu

**Table 6:** Casein values with delayed reaction result excluded

	Mean	Median	Range
SpIgE Casein	1.51 iu	0.53 iu	0.02–12.9 iu

**Conclusions**: Low levels of casein specific IgE give a good indicator of outcome to challenge and aids early selection of patients for baked milk challenges. This is especially important in clinical settings with no on site intensive care where careful challenge risk stratification is important. Early inclusion of dietary baked milk accelerates the resolution of cows’ milk allergy and widens the choice of foods for these children.

The child with casein of 29.8 passed their in-hospital food challenge. When baked milk was introduced at home, she became symptomatic and currently remains milk free. Discarding this result we get the following results

#### P57 Molecular characterisation and immunoreactivity of a peanut ingredient for use in oral food challenges

##### Ivona Baricevic-Jones^1^, Justin T. Marsh^1^, Phil E. Johnson^1^, Anuradha Balasundaram^1^, Anya-May Hope^1^, Aafke Taekema^1^, Angela Simpson^2^, Aida Semic-Jusufagic^2^, E.N. Clare Mills^1^

###### ^1^Institute of Inflammation and Repair, Manchester, United Kingdom; ^2^University Hospital of South Manchester, Manchester, United Kingdom

**Correspondence**: Ivona Baricevic-Jones

*Clinical and Translational Allergy* 2016, **6(Suppl 2)**:P57

**Background**: Although double-blind placebo-controlled food challenge (DBPCFC) is established as the current “gold standard” for food allergy diagnosis, the criteria by which food challenge ingredients should be evaluated for suitability have not been standardised. Furthermore, the amount of allergic material in food challenges should be comparable to commonly consumed materials. We aimed to characterise the peanut flour in a DBPCFC with respect to peanut allergen content and IgE reactivity to determine suitability for use.

**Materials and methods**: Roasted peanut flour (four batches) and raw peanut flour were used. Sera from peanut allergic individuals were obtained from the Manchester Allergy and Respiratory Tissue Bank. Peanut flours were characterised using 2-dimensional (2D) SDS-PAGE, immunoblots, IgE immunoblots, IgE ELISA and mass spectrometry. The peanut flour-incurred challenge meal was used to determine its reactivity with sera from allergic individuals.

**Results**: Peanut flour was characterised by immunoblotting to show the presence of the major allergens (Ara h 1, Ara h 2, Ara h 6 and Ara h 3). Protein aggregation was observed in all the immunoblots as a consequence of roasting (most prominent was aggregation of Ara h 1 and Ara h 3 acidic subunit). Allergens were profiled using untargeted mass spectrometry. IgE immunoblots and immunoassay using sera from allergic patients confirmed consistent patterns of reactivity of peanut flour between batches. The IgE immunoblots performed with extracts of the peanut-incurred dessert challenge meals demonstrated little or no difference in the reactivity compared to the roasted peanut flour itself.

**Conclusions**: Variation between batches of peanut flour with regards to peanut allergen profiles and IgE reactivity was minimal. IgE reactivity of peanut was maintained after being incurred into the chocolate dessert. These data show the suitability of the peanut flour as an active ingredient in oral food challenges.

**Keywords**: Peanut; Allergy; DBPCFC; IgE immunoreactivity

#### P58 Specific IgE to recombinant allergens of hazelnut and oral food challenge in children

##### Gourdon Dubois Nelly^1^, Sellam Laetitia^2^, Pereira Bruno^1^, Michaud Elodie^1^, Messaoudi Khaled^1^, Evrard Bertrand^1^, Fauquert Jean-Luc^1^

###### ^1^CHU, Clermont-Ferrand, France; ^2^CH, Montlucon, France

**Correspondence**: Gourdon Dubois Nelly

*Clinical and Translational Allergy* 2016, **6(Suppl 2)**:P58

**Background**: **Rationale:** Oral Food challenge (OFC) is the milestone to access to a definitive diagnosis of hazelnut allergy (HA). We investigated the relevance of Specific IgE to recombinants allergens of hazelnut available in daily practice.

**Materials and methods**: 31 children (20 M/11F) aged 10.5 ± 4.9 years, were recruited because referred to the allergy outpatient unit of the University Hospital Clermont-Ferrand, France, to practice OFC to hazelnut. All underwent a reaction after ingestion and were sensitized to hazelnut (positive SPT or specific IgE measurements over 0.10 IU/mL). Specific IgE dosages (ImmunoCAP Thermo Fisher^®^) were performed versus cor a1, cor a8, cor a9 and cor a14 and expressed in IU/mL (median, [Q25-Q75]). Single blind placebo controlled OFC was performed the same day according to usual recommendations, as well as skin prick-tests (SPT, positive when the wheel reached 3 mm) and IgE dosages to hazelnut. HA was assessed when an objective reaction occurred after OFC. Comparisons between the groups were performed using the Chi-square tests for categorical variables, and by the Student t-test or Kruskal–Wallis test for quantitative parameters.

**Results**: At baseline, groups with positive OFC (n = 8 OFC+) and negative OFC (n = 23 OFC−) were similar in terms of sex, age, and initial reaction. Specific IgE to hazelnuts recombinant fractions did not differ significantly for cor a1 (5.0 [1.1–18.2] v. 1.2 [0.1–14.2]), cor a8 (0.1 [0.1–0.5] v. 0.1 [0.1–0.2]). Cor a9 specific IgE levels were significantly higher (p = 0.01) in OFC+ patients than in OFC− patients (0.9 [0.3–2.4] v. 0.1 [0.1–0.2]). Cor a14 levels were very significantly higher (p < 0.001) in OFC+ (5.4 [2.2–8.4]) patients than in OFC− patients (0.1 [0.1–0.1]). Specific IgE to hazelnut did not differ significantly in both groups: 3.4 [2.0–20.7] in OFC+ patients, and 1.9 [0.9–11.1] in OFC− patients. SPT to hazelnut were positive in 7 of the 8 OFC+ patients (87.5 %) and 6 of the 23 OFC-patients (26.1 %) (p = 0.004) and the wheel of SPT larger in OFC+ population (9.0 [5.5–11.0]) than in OFC− population (0.5 [0.0–4.0]) (p = 0.004).

**Conclusions**: We concluded to the interest in this population of specific IgE measurements to recombinant fractions cor a9 and cor a14 in addition to SPT in order to predict OFC results on children suspected of HA.

**Keywords**: Hazelnut; Oral food challenge; Recombinant fractions; Specific IgE

### Poster session 7/8: miscellaneous

#### P59 What defines a protein as an allergen? A discussion of sources and sufficiency

##### Richard E. Goodman

###### Dept. of Food Science & Technology, University of Nebraska-Lincoln, Lincoln, United States

**Correspondence**: Richard E. Goodman

*Clinical and Translational Allergy* 2016, **6(Suppl 2)**:P59

**Background**: Reports that proteins are allergens are increasingly common in peer-reviewed journals, open access articles of varied review qualities or by automated computer annotation. The impact of reporting a protein as an allergen can have significant consequences related to diagnostic accuracy and food risk assessment processes. But there is no standard definition accepted for proof of allergenicity. How should we define allergens? Who should define a protein as an allergen? The intent of this discussion is to consider sources of information and definitions focuses on allergens causing IgE mediated hypersensitivities.

**Materials and methods**: Allergens are defined primarily based on IgE binding tests with proteins purified from allergenic sources, proteins produced as clones from allergenic sources or simply by computer sequence comparisons to those of known allergens. While clinical responses to the pure protein could prove that a protein is an allergen, such tests are never conducted due to ethical considerations or lack of appropriately identified, voluntary allergic subjects. Publications typically report predicted (cDNA) translated protein sequences or partially characterized determined amino acid sequences. Subjects are often declared as allergic or sensitized with little or no clinical symptoms reported and without background information related to responses to other allergenic sources. This brief review is intended to stimulate researcher’s efforts.

**Results**: Sources defining allergens have different criteria and purposes. The WHO/IUIS database provides a nomenclature system with a short-hand name for allergenic proteins (genus, species and number), typically prior to publication, based on assurances from the submitter. Criteria are that a minimal number of allergic subjects (typically 5), produce IgE that binds to the protein. The AllergenOnline.org database expert panel reviews published information of IgE binding to the taxonomic-protein group with a minimal requirement of specific IgE binding with preference for demonstration of allergenic activity (skin prick test, basophil activation) for risk assessment. Sequence sources (NCBI/UniProt) are increasingly auto-annotated based on keywords or low identity matches. Cross-reactive carbohydrate determinants can skew results

**Conclusions**: The burden of proof is on you, the allergen researcher, to clearly report objective characteristics and results!

#### P60 Cat allergy: relationship between clinical and molecular diagnostic

##### María Cecilia Martín Fernández De Basoa, Antón Fernández Ferreiro, Elena Rodríguez Plata

###### University Hospital Nuestra Señora de Candelaria, Santa Cruz De Tenerife, Spain

**Correspondence**: María Cecilia Martín Fernández De Basoa

*Clinical and Translational Allergy* 2016, **6(Suppl 2)**:P60

**Background**: Allergy to cat is a frequent cause of rhinitis, asthma or contact urticaria. The prevalence of sensitization to different cat allergens is not well known but it seems that different patterns of sensitization to cat allergens in patients with cat allergy can help us to predict the severity and persistence of symptoms.

**Materials and methods**: We select 39 sensitized patients to cat (51 % female, median age 28 years). Specific IgE measurement to cat allergens Fel d1, Fel d2 and Fel d4 was performed by microarray ISAC^®^ (ThermoFisher Scientific, Sweden), a value >0.3 ISU was considered as positive. Skin prick test was performed with an ALK-Abelló extract (Denmark). Association of Specific IgE measurements and presence and type of rhinitis or asthma was studied . Statistical analysis was performed using Fisher test and chisquared test.

**Results**: 95 % of patients had specific IgE to Fel d1, 10 % to Fel d2, and 10 % to Fel d4. 82 % were monosensitized to Fel d1, 3 % to Fel d2 and 3 % to Fel d4. 75 % of sensitized patients to Fel d4 were also sensitized to Fel d1. Fel d2 was associated with severity of rhinitis and asthma (p < 0.01, p < 0.01, respectively). Fel d4 was associated with presence of asthma symptoms (p < 0.04). Direct contact with cats was associated both to persistence and severity of rhinitis (p < 0.01, p < 0.01, respectively). A positive skin prick test to cat was associated with rhinitis and asthma symptoms (p < 0.01, p < 0.01, respectively).

**Conclusions**: The relationship between clinical and molecular diagnostic in our population was similar to those of other Mediterranean-based studies.

**Keywords**: Cat allergy

#### P61 Anaphylaxis to rabbit: the cat came in last

##### Luis Amaral^1^, Borja Bartolomé^2^, Alice Coimbra^1^, Jose L Placido^1^

###### ^1^Serviço de Imunoalergologia, Centro Hospitalar São João, Porto, Portugal; ^2^Bial Aristegui, Bilbao, Spain

**Correspondence**: Luis Amaral

*Clinical and Translational Allergy* 2016, **6(Suppl 2)**:P61

**Background**: Although rabbits are common domestic pets, severe allergic reactions to rabbits at home are rarely reported. There are some cases of mild to moderate allergic rhinitis, conjunctivitis, pruritus and/or asthma in laboratory animal caretakers with frequent exposure. Moreover, few studies have evaluated the allergenic relationship between rabbits and other furry animals.

We present the case of a 52 year-old woman, who presented with anaphylaxis minutes after inhalant exposure to rabbits in September 2013. She had a history of asthma and rhinitis. She worked as a cook since 1987 and some of her tasks involved, occasionally, preparing rabbit for meals, including skinning.

**Materials and methods**: Skin prick tests were positive to house dust mites and cat epithelium. Prick-to-prick test was positive to raw rabbit meat. ImmunoCAP^®^ test revealed high values of total IgE (1072 kU/L) as well as specific IgE to rabbit epithelium (28.2 kU/L) and rabbit meat (14.4 kU/L). Sodium dodecye sulfate polyacrylamide gel electrophoresis (SDS-PAGE) immunoblotting with extracts from rabbit (meat, epithelium, urine) and cat (epithelium, albumin) was performed.

**Results**: Two IgE-binding bands with molecular masses of 50–60 kDa were identified. SDS-PAGE immunoblotting-inhibition was carried out using cat epithelium extract as solid phase. All rabbit extracts assayed (meat, epithelium, urine) were able to induce a total IgE binding inhibition as well as a sample of cat albumin.

**Conclusions**: Sensitization to cat albumin and rabbit proteins (50–60 kDa) were detected, which were probably different isoforms of rabbit albumin. These results suggest that rabbit was probably the primary sensitizer, contrary to what was expected and highlight the importance of molecular diagnosis.

#### P62 Dog allergy: relationship between clinical and molecular diagnostic

##### María Cecilia Martín Fernández De Basoa, Antón Fernández Ferreiro, Elena Rodríguez Plata

###### University Hospital Nuestra Señora de Candelaria, Santa Cruz De Tenerife, Spain

**Correspondence**: María Cecilia Martín Fernández De Basoa

*Clinical and Translational Allergy* 2016, **6(Suppl 2)**:P62

**Background**: Allergy to dog is a frequent cause of rhinitis, asthma or contact urticaria. The prevalence of sensitization to different dog allergens is not well known but it seems that different patterns of sensitization to dog allergens in patients with dog allergy can help us to predict the severity and persistence of symptoms.

**Materials and methods**: We select 41 sensitized patients to dog (61 % female, median age 31 years). Specific IgE measurement to dog allergens Can f1, Can f2, Can f3 and Can f5 was performed by microarray ISAC^®^ (ThermoFisher Scientific, Sweden), a value >0.3 ISU was considered as positive. Skin prick test was performed with an ALK-Abelló extract (Denmark). Association of Specific IgE measurements and presence and type of rhinitis or asthma was studied. Statistical analysis was performed using Fisher test and chisquared test.

**Results**: 44 % of patients had specific IgE to Can f1, 17 % to Can f2, 12 % to Can f3, and 66 % to Can f5. 20 % were monosensitized to Can f 1, 2 % to Can f2, 5 % to Can f3 and 44 % to Can f5. Can f1 was associated with persistent rhinitis (p 0.01), Can f3 with severity of rhinitis and asthma (p < 0.01, p 0.01, respectively), and Can f5 to both persistence and severity of rhinitis (p 0.02, p < 0.01, respectively). Sensitization to several allergens in patients (1, 2, 3 or 4) was associated with persistent asthma or rhinitis (p 0.02, p 0.01, respectively), and with moderate severity (p 0.04). Direct contact with dogs was associated with both, persistency and severity of rhinitis (p 0.02, p 0.03, respectively). The wheal diameters of skin test with commercial extract of dog were smaller in patients monosensitized to Can f 5.

**Conclusions**: The relationship between clinical and molecular diagnostic in our population was similar to those of other Mediterranean-based studies.

**Keywords**: Dog allergy

#### P63 Correlation of serum timothy grass-pollen specific IgE levels determined by two immunoblot test systems

##### Mariana Vieru^1^, Florin-Dan Popescu^1^, Florin-Adrian Secureanu^2^, Carmen Saviana Ganea^2^

###### ^1^Carol Davila University of Medicine and Pharmacy, Bucharest, Romania; ^2^Nicolae Malaxa Clinical Hospital, Bucharest, Romania

**Correspondence**: Mariana Vieru

*Clinical and Translational Allergy* 2016, **6(Suppl 2)**:P63

**Background**: Timothy grass (*Phleum pratense*) belongs to the *Pooideae* subfamily and it is one of the most significant source of grass pollen allergens in temperate regions.

**Materials and methods**: We compared the levels of grass pollen-specific IgE measured in non-haemolytic, non-lipaemic, non-icteric serum samples from patients with genuine sensitization to Timothy grass pollen, evidenced *in vitro* by *Poaceae* family-specific IgE biomarker (beta-expansin rPhl p 1) and/or *Pooideae* subfamily-specific IgE biomarker (ribonuclease rPhl p 5), using two immunoblot test systems: a blot line assay for IgE antibodies against eleven raw aeroallergen extracts and a multi-parameter test system for nine pollen single purified allergen components and two whole pollen allergen extracts. Both immunoblot test procedures are based on line blot system technology, with allergens in parallel lines at defined positions on membrane strips, and consist of three basic steps: serum incubation, enzyme conjugate (alkaline phosphatase-labelled anti-human IgE, monoclonal) incubation and chromogenic substrate (nitroblue tetrazolium chloride/5-bromo-4-chloro-3-indolylphosphate) incubation.

**Results**: Twenty serum samples analyzed with the single purified allergen components test kit revealed IgE sensitization to rPhl p 1 and rPhl p 5 in 60 % of cases, the rest presenting specific IgE only to rPhl p 1 (35 %) or only to rPhl p 5 (5 %). The differences that we found between the values of *Phleum pratense* pollen-specific IgE determined with this multiparameter test system (47.38 ± 27.41 kU/L) and the values measured with the blot line inhalation test system (41.53 ± 28.26 kU/L) in all serum samples were not practically significant, and the enzyme allergosorbent test/EAST class correlation was really acceptable.

**Conclusions**: In patients with suspected grass pollen allergy, the component-resolved multiparameter immunoblot test system provides a reliable and costly efficient *in vitro* assessment of Timothy grass pollen-specific IgE with a very good correlation with the measurement using the line blot test system with raw allergen extracts, besides allowing an in-depth characterisation of sensitization to disease-causing (Phl p1, Phl p5) and cross-reacting (Phl p 7, Phl p 12) allergen components from grass pollen on one test strip.

#### P64 Development of oral food challenge formulations for diagnosis of fish allergy using powdered fish ingredients

##### Carol Ann Costello^1^, Ivona Baricevic-Jones^1^, Martin Sorensen^2^, Clare Mills^1^, Adrian Rogers^3^, Aage Otherhals^4^

###### ^1^University of Manchester, Manchester, United Kingdom; ^2^University Hospital of North Norway, Tromsoe, Norway; ^3^Romer Labs, Runcorn, United Kingdom; ^4^Nofima, Tromsoe, Norway

**Correspondence**: Carol Ann Costello

*Clinical and Translational Allergy* 2016, **6(Suppl 2)**:P64

**Background**: The “gold standard” of food allergy diagnosis is double blind placebo controlled food challenge. There has been little standardisation of challenge materials over the years and the EU labelling requirements specify for “fish” to be labelled rather than specific fishes. As part of a study on fish allergy standardised oral food challenge meals for the diagnosis of allergy to different fishes (cod, salmon and mackerel) have been developed.

**Materials and methods**: A set of powdered fish ingredients were obtained and assessed with regards protein profile and immunoreactivity using an antibody specific for cod parvalbumin. Oral food challenge meals were developed based on those used in the EuroPrevall project using powdered cod, salmon and mackerel. Blinding was evaluated using trained sensory panellists (n = 9). The homogeneity and dose verification of the challenge meals were assessed using commercially available ELISA kit utilising a cod parvalbumin antibody.

**Results**: Fish powder protein profiles were evaluated compared to samples of fish flesh. The content of parvalbumin evaluated and demonstrated that mackerel had the lowest content and cod the highest content of parvalbumin allergen. The protein content of fish powders varied widely reflecting different fishes and manufacturing procedures. Once incorporated into the challenge matrix there was no noticeable variation between placebo and fish-containing challenges for salmon and cod. Blinding at a higher dose was not possible for the mackerel due to its’ strong characteristics and lower protein content.

**Conclusions**: Challenge meals can be prepared for a range of fishes but quality of blinding is dependent on access to quality powdered fish ingredients with a high protein content. Parvalbumin content of fish powders was ranked cod > salmon > mackerel suggesting that both IgE-reactivity determined in vitro and clinical reactivity would be lower for mackerel than cod challenges. Further studies in vitro and in vivo will test this proposition.

**Keywords**: Fish double blind placebo controlled food challenge

#### P65 Fish and peanut allergens interact with plasma membranes of intestinal and bronchial epithelial cells and induce differential gene expression of cytokines and chemokines

##### Tanja Kalic^1^, Isabella Ellinger^1^, Chiara Palladino^1^, Barbara Gepp^1^, Eva Waltl^2^, Verena Niederberger-Leppin^2^, Heimo Breiteneder^1^

###### ^1^Department of Pathophysiology and Allergy Research, Medical University of Vienna, Vienna, Austria; ^2^Department of Otorhinolaryngology, Medical University of Vienna, Vienna, Austria

**Correspondence**: Tanja Kalic

*Clinical and Translational Allergy* 2016, **6(Suppl 2)**:P65

**Background**: Fish and peanut allergies are among the most dangerous food allergies. Symptoms occur by allergen exposure via gastrointestinal or respiratory tracts. Food matrix components may contribute to the immune response. We explored interactions of intestinal and bronchial epithelial cells with the major Atlantic cod allergen Gad m 1, or a peanut allergen Ara h 1, with or without codfish-derived food matrix or peanut lipids.

**Materials and methods**: Caco-2 and 16HBE14o- cells were used as *in vitro* models for human intestinal and bronchial epithelial cells, respectively. To explore the type of interaction between the allergens and the cells, confluent cells were treated with fluorescently labelled Gad m 1 with or without codfish matrix, or with Ara h 1 with or without peanut lipids. Labelled allergens were detected by confocal microscopy. To explore cytokine and chemokine gene expression in treated cells, total RNA was isolated and mRNA levels of CCL20, MCP-1, TSLP, IL-6 and IL-8 were determined by qRT-PCR.

**Results**: In Caco-2 cells, both allergens bound to the apical plasma membrane. In 16HBE14o- cells, Gad m 1 localized to the lateral membrane domain (bellow ZO-1 level), while Ara h 1 interacted with the apical and lateral (above ZO-1 level) membrane domains. Co-treatment with Ara h 1 and peanut lipids reduced the fluorescent Ara h 1 signal in 16HBE14o-cells. In both cell lines, treatments with the allergens and codfish matrix/peanut lipids induced differential gene expression of cytokines and chemokines. Both allergens when applied alone reduced CCL20 mRNA levels in Caco-2 cells by 25 % and mRNA levels of all the explored genes in 16HBE14o-cells by 30–70 %. Gad m 1 also decreased MCP-1 gene expression in Caco-2 cells by 30 %. Codfish matrix increased IL-6 mRNA levels in both cell lines by 1.6–3-fold. Peanut lipids decreased MCP-1 mRNA levels by 20 % in Caco-2 cells and by 30 % in 16HBE14o-cells. TSLP mRNA levels were increased by all treatments in Caco-2 cells (1.5–2.5-fold), while in 16HBE14o- cells all the treatments downregulated TSLP gene expression by 1.5–2.5-fold.

**Conclusions**: Fish and peanut allergens interact with plasma membranes of intestinal and bronchial epithelial cells, but are not internalized. This interaction induces signalling in the cells, which is further modulated by food matrix components and may contribute to allergic sensitization and reaction.

Supported by the Austrian Science Fund grants SFB 4608 and 4613 and the doctoral program W1248-B1.

**Keywords**: Fish allergy; Peanut allergy; Food matrix; Epithelial cells

#### P66 Interleukin 4 affects fat tissue metabolism and expression of pro-inflammatory factors in isolated rat adipocytes

##### Dawid Szczepankiewicz^1^, Ewa Pruszynska-Oszmalek^1^, Marek Skrzypski^1^, Krzysztof W. Nowak^1^, Aleksandra Szczepankiewicz^2^

###### ^1^Poznan University of Life Sciences, Poznan, Poland; ^2^Poznan University of Medical Sciences, Poznan, Poland

**Correspondence**: Aleksandra Szczepankiewicz

*Clinical and Translational Allergy* 2016, **6(Suppl 2)**:P66

**Background**: Interleukin 4 is an inflammatory cytokine that initiate conversion of T cells towards Th2 population and IgE production specific for allergy and asthma. It also stimulates monocytes and macrophages to produce other pro-inflammatory cytokines thus enhancing inflammation. Recent studies showed that IL-4 influences metabolism of adipocytes indicating the relationship between allergic inflammation and obesity.

The aim of this study was to investigate the effect of IL-4 exposure on the metabolism of rat adipocytes and the expression of pro-inflammatory factors in these cells.

**Materials and methods**: Adipocytes were isolated from epididymal fat pad. After isolation, adipocytes were incubated with IL-4 at different doses (1, 10, 100 nM) for 2 h. After incubation, lipogenesis (both basal and insulin-stimulated) and lipolysis (basal and isoproterenol-stimulated) were measured. Perilipin, GLUT4 and phosphorylation of HSL were measured by Western blot following 8 h of incubation with IL-4. Pro-inflammatory (leptin, resistin) and anti-inflammatory (adiponectin) hormones concentration was measured in cell medium using ELISA kits. Gene expression of *Tnfα, Crp, IL-6* and *IL-4R* was measured by real-time PCR.

**Results**: Incubation with IL-4 resulted in increased basal lipogenesis and decreased isoproterenol-stimulated lipolysis in rat adipocytes. We also observed significant increase of GLUT4 and decrease of phospho-HSL protein levels. Expression of perilipin decreased however, this change was not significant. Moreover, we found increased concentration of leptin and resistin and decreased concentration of adiponectin in cell medium. Gene expression analysis showed increased expression for all studied factors.

**Conclusions**: Results obtained in our study indicate that IL-4 may play a role in increased fat deposition and represents a link between obesity and other inflammatory conditions such as asthma.

The study was supported by the Polish National Science Centre, grant no. 2011/01/D/NZ5/02771.

#### P67 Ozone induced airway hyperreactivity in PD-L2−/− mice model

##### Gwang-Cheon Jang

###### NHIS ILSAN Hospital, Ilsan, Korea

**Correspondence**: Gwang-Cheon Jang

*Clinical and Translational Allergy* 2016, **6(Suppl 2)**:P67

**Background**: Exposure to ozone, which is a major component of air pollution, induces a form of asthma that occurs in the absence of adaptive immunity. Although ozone-induced asthma is characterized by airway neutrophilia, and not eosinophilia, it is nevertheless associated with airway hyperreactivity (AHR), which is a cardinal feature of asthma. Programmed death-1 (PD-1) with its ligands, programmed death ligand B7DC (PD-L2) and was shown to regulate T-cell activation and tolerance. PD-1 has been characterized as a negative regulator of conventional CD4^+^ T cells. Recent studies have demonstrated that PD-L2 have important roles in modulating and polarizing T-cell functions in allergen induced AHR

**Materials and methods**: Airway inflammation and AHR was measured in mice 24 h after the final ozone exposure. Mice was divided 4 groups; 1. Air exposure BALB/c (WT air), 2. Air-PDL2−/− mice (PDL2KO air), 3. ozone exposure BALB/c (WT O3), and 4. Ozone-PDL2−/− mice (PDL2KO O3) on day 6. Mice were placed awake in individual wire mesh cages inside a stainless steel and Plexiglas exposure chamber and exposed to ozone (1 ppm) for 3 h on day 1, day 3 and day 5. For room air exposure, a separate and identical exposure chamber was used. 

**Results**: The severity of AHR and airway inflammation is significantly reduced in PDL2KO O3 group of mice compared with WT O3 group of mice. 

**Conclusions**: This study indicate that PD-L2 have important roles in the regulation of ozone-induced AHR and airway inflammation as well as allergen-induced AHR and airway inflammation

**Keywords**: Ozone; Airway hyperreactivity; PDL2−/− mice

#### P68 Thymic stromal lymphopoietin (TSLP) and its receptor as targets for the development of anti-inflammatory inhibitory agents

##### Iva Markovic^1^, Andreas Borowski^1^, Tina Vetter^1^, Andreas Wohlmann^1^, Michael Kuepper^2^, Karlheinz Friedrich^1^

###### ^1^Institute for Biochemistry II, University Hospital Jena, Jena, Germany; ^2^Department of Pneumology, University of Rostock, Rostock, Germany

**Correspondence**: Iva Markovic

*Clinical and Translational Allergy* 2016, **6(Suppl 2)**:P68

The published version of this abstract can be found at [1].

**Reference**

(2015), Poster Sessions. FEBS J, 282: 55–422. doi:10.1111/febs.13339

#### P69 The mononuclear phagocyte system in experimentally-induced allergic rhinitis

##### Ibon Eguiluz Gracia^1^, Anthony Bosco^2^, Ralph Dollner^3^, Guro Reinholt Melum^1^, Anya C Jones^2^, Maria Lexberg^1^, Patrick G Holt^2^, Espen Sønderaal Bækkevold^1^, Frode Lars Jahnsen^1^

###### ^1^Department of Pathology and Center for Immune Regulation. Oslo University Hospital-Rikshospitalet and University of Oslo, Oslo, Norway; ^2^Telethon Kids Institute. The University of Western Australia., Perth Wa, Australia; ^3^Department of Otorhinolaringology, Head and Neck Surgery. Oslo University Hospital-Rikshospitalet., Oslo, Norway

**Correspondence**: Ibon Eguiluz Gracia

*Clinical and Translational Allergy* 2016, **6(Suppl 2)**:P69

**Background**: Activated Th2 cells and eosinophils are hallmarks of the allergic inflammation in allergic rhinitis. However, which cells activate and attract T cells and eosinophils to the inflammatory lesion has not been determined. The aim of this study was to assess the role of mucosal mononuclear phagocytes, consisting of monocytes, macrophages and dendritic cells, in the local allergic inflammatory reaction.

**Materials and methods**: Allergic rhinitis patients and non-atopic controls were challenged with pollen extract and nasal symptoms were recorded. Mucosal biopsies, obtained at different time points before and after challenge, were used for immunostaining in situ and flow-cytometric cell sorting. Sorted mononuclear phagocytes were subjected to RNA extraction and gene expression profiling.

**Results**: In an in vivo model of allergic rhinitis we found that CD14+ monocytes were recruited to the nasal mucosa within hours after local allergen-challenge, whereas conventional dendritic cells accumulated after several days of continued provocation. On the other hand the number of resident macrophages and plasmacytoid dendritic cells remained unchanged the first hours after allergen challenge. Transcriptomic profiling of mucosal mononuclear phagocytes sorted after one week of continued allergen-provocation showed an activated phenotype, at least partially driven by IL-4 and/or IL-13 signalling. Importantly, gene expression of several Th2-related chemokines was significantly upregulated by the mononuclear phagocyte population concomitant with an increased recruitment of CD4+ T-cells and eosinophils. Moreover the number of mucosal GATA3+ T cells increased 48 h after allergen dose compared to baseline.

**Conclusions**: Our findings suggest that the mononuclear phagocyte population is directly involved in the production of proinflammatory chemokines that attract other immune cells. Rapid recruitment of CD14+ monocytes to the challenged site indicates that these proinflammatory mononuclear phagocytes have a central role in orchestrating the allergic inflammation locally.

**Keywords**: Monocytes; Dendritic cells; Grass; Birch

#### P70 Expression of histamine metabolizing enzymes is increased in allergic children

##### Aleksandra Szczepankiewicz^1^, Paulina Sobkowiak^1^, Marta Rachel^2^, Beata Narozna^1^, Dorota Jenerowicz^1^, Witold Swiatowy^1^, Anna Breborowicz^1^

###### ^1^Poznan University of Medical Sciences, Poznan, Poland; ^2^Department of Allergy, Provincial Hospital No 2, Rzeszów, Poland

**Correspondence**: Aleksandra Szczepankiewicz

*Clinical and Translational Allergy* 2016, **6(Suppl 2)**:P70

**Background**: Neuroimmunologic factors are important in the pathogenesis and the course of allergic diseases such as allergic rhinitis and atopic dermatitis. Neuropeptides and neurotrophins released in the airways and skin upon allergic response also mediate neurogenic inflammation, thus modifying disease symptoms and severity.

Therefore, we investigated if neurogenic inflammation genes are involved in the pathogenesis of allergic diseases (atopic dermatitis, allergic rhinitis) in paediatric population.

**Materials and methods**: In the analysis we included 23 patients, 11 patients with AR and 12 patients with AD aged between 6 and 18 years. Diagnosis of allergic AR was made according to ARIA guidelines and AD was diagnosed based on diagnostic criteria defined by the American Academy of Dermatology. Diagnosis was made at least 6 months before inclusion in the study. The control group consisted of 21 healthy children without past and current symptoms of allergy and asthma. The expression of 31 genes associated with neurogenic inflammation from peripheral blood leukocytes was analyzed with use of TaqMan Low Density Array method. As an endogenous control we used an assay specific for 18S rRNA gene. The target genes involved neurotrophins and their receptors, tyrosine kinases, neuropeptides, histamine metabolism pathway, neurokines and receptors of ion channels.

**Results**: In allergic patients we observed significant increase in expression of genes encoding two histamine metabolizing enzymes (*HNMT, ABP1*) as compared to the healthy children. For atopic dermatitis significantly increased expression was found for *ABP1* (RQ = 35, p = 0.037) and *HNMT* (RQ = 8.5, p = 0.041), for allergic rhinitis patients we observed increased expression of *ABP1* (RQ = 26.4, p = 0.043). Moreover, when comparing AD to AR patients, we found that expression of 3 genes was increased in AR (*IL4Ra, MAPK1, MME*) as compared to AD, but the difference was not statistically significant.

**Conclusions**: Increased expression of histamine metabolizing enzymes (*HNMT*, *ABP1*) in allergic diseases (AR, AD) suggest that induction of histamine metabolizing enzymes might be an important control mechanism for the inflammation induced by histamine during allergic inflammation.

The study was supported by the Polish National Science Centre, grant no. 2011/01/D/NZ5/02771.

**Keywords**: Allergic rhinitis; Atopic dermatitis; Neurogenic inflammation; Children

#### P71 Modifying the glycosylation of human IgE towards oligomannosidic structures does not affect its biological activity

##### Melanie Plum^1^, Sara Wolf^1^, Frank Bantleon^1^, Henning Seismann^2^, Frederic Jabs^1^, Michaela Miehe^1^, Thilo Jakob^3^, Edzard Spillner^1^

###### ^1^Aarhus University, Aarhus, Denmark; ^2^Euroimmun AG, Lübeck, Germany; ^3^University Medical Center, Freiburg, Germany

**Correspondence**: Melanie Plum

*Clinical and Translational Allergy* 2016, **6(Suppl 2)**:P71

**Background**: IgE is a mechanistically and structurally outstanding isotype and carries a pronounced glycosylation. Most glycans are of complex type, only one glycan is an oligomannosidic structure. Mutational analyses have shown that the oligomannosidic glycan is indispensable for activity. The role of the complex type glycans however remains unclear.

**Materials and methods**: In order to engineer the glycosylation of human IgE we established the recombinant production of IgE in insect cells that do not attach complex type glycans. IgE Fc variants were produced in mammalian cells. The proteins were purified via affinity chromatography and characterised by ELISA, immunoblotting and SPR. RBL assays were used to prove biological function and the glycosylation was assessed by mass spectrometry.

**Results**: Upon expression in Sf9 insect cells recombinant human IgE (rIgE) was efficiently assembled and secreted into culture supernatant with yields of >30 mg/L. The rIgE exhibited a highly specific interaction with its antigen, anti-IgE antibodies and IgE receptors. Glycoproteomic analysis revealed the presence of a prototypic oligomannosidic *N*-glycosylation at several positions of the epsilon heavy chain. Complex type glycans were not found. While mutation of the oligomannose structure in the IgE Fc broadly abrogated effector cell activation mediator release assays demonstrated a biological activity of the glycomodified IgE comparable to that of IgE containing complex type glycans.

**Conclusions**: In summary the engineering of IgE with a fully oligomannosidic glycosylation does not affect the molecular characteristics as high affinity receptor and antigen binding and biological activity. Our data will contribute to the understanding and potential use of this important antibody isotype.

#### P72 Flying Labs: an educational initiative to transfer allergy research into high-school settings

##### Michael Wallner^1^, Heidi Hofer^1^, Fatima Ferreira^1^, Reinhard Nestelbacher^2^

###### ^1^University of Salzburg, Salzburg, Austria; ^2^DNA-Consult Sciencetainment, Ostermiething, Austria

**Correspondence**: Michael Wallner

*Clinical and Translational Allergy* 2016, **6(Suppl 2)**:P72

**Background**: Science communication is an integral part of modern research and has been since more than a decade an important activity of the allergology research teams in Salzburg. The Flying Labs are one of these initiatives. Within this concept, we are able to bring a molecular biology lab into a school setting where, according to well-established protocols, high-school students can perform experimental work directly in the classroom using up to date equipment. The course schedule is designed in way that the students themselves can elaborate simple research questions, which are usually inspired by their everyday life. Besides a gene-analysis-lab, since 2009, a special Flying Lab has been dedicated to immunological and allergy-related topics.

**Materials and methods**: The Sparkling Science Project Bio KoSMoS (www.biokosmos.org), funded by the Austrian Ministry of Science, Research, and Economy (BMWFW), allowed us to further develop the Flying lab initiative and to test educational concepts such as “open innovation”, mentoring systems, and “citizen science”.

**Results**: We elaborated a novel course in which students get basic knowledge and experimental training in recombinant protein production at their schools, using fluorescent proteins as models. These students were invited to participate in “science days” at the University of Salzburg for additional training in basic techniques such as SDS-PAGE and Western blots with allergens. After this initial training program, we tested a cell-free expression platform for allergenic molecules and first experiments together with the students were already performed.

**Conclusions**: We demonstrated that initiatives such as Flying Labs can be used to successfully transfer modern science ideas and principles to schools. In order to contribute to scientific questions, interested individuals, i.e. high school students, need to acquire a certain level of know-how and training. Thereafter, scientific questions can be addressed in a truly collaborative manner. We are convinced that this open innovation initiative will not only inspire young people to think and work scientifically, but will also help researchers to establish new methods for allergen expression and analysis.

The work was supported by the Sparkling Science project SPA 05-193 and the priority program of the University of Salzburg (Allergy-Cancer-BioNano Research Centre).

#### P73 Clinical significance of antihistamines and Kujin, an anti-allergic Kampo medicine

##### Hiroyuki Fukui

###### Tokushima University, Tokushima, Japan

**Correspondence**: Hiroyuki Fukui

*Clinical and Translational Allergy* 2016, **6(Suppl 2)**:P73

**Background**: The target molecule of antihistamines is histamine H_1_ receptor. However, pathological mechanism targeted by antihistamines remains to be elucidated.

**Materials and methods**: Histamine H_1_ receptor mRNA was determined by real-time quantitative RT-PCR. Activation of protein kinase C-delta (PKCδ) was assessed by immunoblot analysis. Nasal hypersensitivity model rats sensitized with toluene 2,4-diisocyanate sensitized (TDI) were used. Nasal symptoms were scored by sneezing, rhinorrhea and nasal swelling. Clinical study was approved by the Ethical Committees of Tokushima Universith Hospital.

**Results**: The stimulation of H_1_ receptors up-regulated the level of histamine H_1_ receptors, consequently the H_1_ receptor mediated signalling propagated. The stimulation of H_1_ receptors phosphorylated protein kinase C-delta (PKCδ) and rottlerin, an inhibitor of PKCδ, suppressed H_1_ receptor gene expression. A positive correlation between nasal symptoms with sneezing and rhinorrhea, and the level of histamine H_1_ receptor mRNA was observed in the nasal mucosa of pollinosis patients. Antihistamines improved both nasal symptoms and the elevated level of H_1_ receptor mRNA either in the pollinosis patients or in the nasal hypersensitivity model rats.

*Kujin* is an anti-allergic *Kampo* medicine. Similar to antihistamines, Kujin extract improved both nasal symptoms and the elevated level of H_1_ receptor mRNA in nasal hypersensitivity model rats. (−)Maackiain was isolated from *Kujin* extract. (−)Maackiain suppressed histamine- or PMA-induced PKCδ phosphorylation. Heat-shock protein 90 (Hsp90) was reported to form a complex with PKCδ and (−)maackiain disrupted the complex. Hsp90 inhibitors suppressed histamine H_1_ receptor gene expression and improved nasal symptoms in allergic model rats.

**Conclusions**: Proliferation of histamine H_1_ receptor mediated signalling was thought to worsen allergic symptoms. All these data suggested that histamine H_1_ receptor gene is an allergic disease-sensitive gene, and antihistamines as well as Kujin improve symptoms by suppressing H_1_ receptor-mediated gene expression signalling at different steps.

**Keywords**: Histamine H1 receptor gene; Disease sensitive gene; Protein kinase C-delta; Heat shock protein 90

